# Inhibition of Triacylglycerol Accumulation and Oxidized Hydroperoxides in Hepatocytes by *Allium cepa* (Bulb)

**DOI:** 10.3390/antiox14060653

**Published:** 2025-05-29

**Authors:** Dya Fita Dibwe, Saki Oba, Satomi Monde, Shu-Ping Hui

**Affiliations:** 1 Faculty of Health Sciences, Hokkaido University, Kita-12, Nishi-5, Kita-Ku, Sapporo 060-0812, Japan; dibwedf@hs.hokudai.ac.jp; 2 Graduate School of Health Sciences, Hokkaido University, Kita-12, Nishi-5, Kita-Ku, Sapporo 060-0812, Japan; saki.oba.h@gmail.com (S.O.); monde.satomi.p3@elms.hokudai.ac.jp (S.M.)

**Keywords:** functional foods, nutraceutical, hepatosteatosis, neutral lipids, oxidative lipidomics, dereplication, ^13^C-NMR, metabolomics, metabolites

## Abstract

Recent studies have demonstrated that dietary plant extracts can inhibit the development of lipid droplets (LDs) and oxidized LDs (oxLDs) in hepatic cells. These findings suggest that such extracts may be beneficial in combating metabolic dysfunction-associated fatty liver disease (MAFLD) and its more advanced stage, metabolic dysfunction-associated steatohepatitis (MASH). We examined nine *Allium* extracts (ALs: AL1–9) to assess their capacity to decrease lipid droplet accumulation (LDA) and oxidative stress by suppressing lipid formation and oxidation in liver cells. Among the *Allium* extracts tested, AL6 exhibited significant inhibitory effects against LDA. Furthermore, we employed our lipidomic method to assess the accumulation and suppression of intracellular triacylglycerol (TAG) and oxidized TAG hydroperoxide [TG (OOH) n = 3] by AL6 in liver cells under oleic acid (OA) and linoleic acid (LA) loading conditions. These findings indicate that foods derived from *Allium* species prevent the formation of lipid droplets by decreasing intracellular lipids and lipid hydroperoxides in the hepatocytes. Analysis of the metabolome of bioactive lipid droplet accumulation inhibition (LDAI) AL6 using LC-MS/MS and 1D-NMR [^1^H, ^13^C, DEPT 90, and 135] techniques revealed that AL6 is primarily composed of carbohydrates, glucosidic metabolites, and organosulfur compounds, with small amounts of polyols, fatty acyls, small peptides, and amino acids. This implies that AL6 could be a valuable resource for developing functional foods and drug discovery targeting metabolic dysfunction-associated fatty liver disease (MAFLD)/metabolic dysfunction-associated steatohepatitis (MASH) and related disorders.

## 1. Introduction

Oleic acid and palmitic acid are the most common fatty acids in the human body. Various fatty acids are readily transformed into triacylglycerols (TAGs). Under certain oxidative stress conditions, TAGs are oxidized to form triacylglycerol hydroperoxide (TGOOH). Understanding how these compounds affect the absorption of intracellular components and their toxicity to cells is essential to understanding the mechanisms of MASH [1,2]. Numerous studies have demonstrated a link between excessive intracellular lipid droplet accumulation (LDA) and metabolic conditions such as obesity and diabetes [3,4]. Researchers believe that hepatic LDA plays a role in the early development of metabolic dysfunction-associated fatty liver disease (MAFLD) [5]. Accumulation of excessive free fatty acids in the liver, known as lipotoxicity, is a key factor in the advancement of MAFLD. Hepatocytes absorb large quantities of free fatty acids from the blood, resulting in the presence of both local and circulating fatty acids. These free fatty acids induce various negative effects, including insulin resistance, hyperlipidemia, inflammation, and hepatic lipidosis, as reported in several studies [6,7,8]. Oxidative stress caused by inflammation often leads to chronic diseases by promoting the accumulation of reactive oxygen species (ROS), which can damage cellular components, including DNA, proteins, and lipid membranes. The oxidation of lipid radicals’ results in lipid peroxidation, which leads to the accumulation of neutral lipids and their oxidized forms within the cells. This accumulation has been associated with various human metabolic conditions, including MAFLD/metabolic dysfunction-associated steatohepatitis (MASH) [9,10,11,12].

MAFLD is a global issue, recognized as the most prevalent chronic liver disease that can lead to fibrosis. The pathological mechanism is intricate and not well understood. The primary factors are insulin resistance and lipid buildup (lipotoxicity), leading to free radical reactions due to multiple factors, which result in lipid peroxidation, necroinflammation, fibrosis, and disease progression. The pathomechanism of MASLD is also not fully understood, and insights into the pathomechanical process are needed. The treatment of MASLD remains an unresolved problem, with lifestyle changes (diet and exercise) and addressing underlying metabolic issues (hyperglycemia and hyperlipidemia) being the mainstay of therapy [13,14,15]. Lifestyle modification is the only proven therapy for MAFLD, with the exception of resmetirom, which has recently been approved by the FDA. Natural products could be a promising therapy for MAFLD; therefore, antioxidants from natural origin may be an alternative treatment option [16]. Natural components found in functional foods and nutraceuticals can decrease the accumulation of lipid droplets (LDs) and oxidized lipid droplets (oxLDs) within liver cells by affecting the neutral lipids TAG and TGOOH. These natural substances are widely available and show significant promise in preventing MAFLD/MASH and drug development, as shown in earlier research [17,18,19,20,21,22,23]. Recent investigations into the effects of natural plant products on the formation of lipid droplets and lipid hydroperoxides in liver cells, along with metabolomic analysis, are emerging as a strong strategy for the prevention and discovery of drugs for MAFLD [24,25,26,27]. Substances with biological activity that inhibit LDA (LDAI) in liver cells have shown potential as candidates for treating MAFLD/MASH.

The primary approach to addressing hepatic steatosis involves the inhibition of LDA and lipid peroxidation. Recent studies have shown that extracts from plant-based foods and their secondary metabolites possess LDAI properties. These substances effectively controlled LD accumulation, thereby demonstrating significant LDAI activity. Extracts from plants and biologically active substances with LDAI characteristics decrease the accumulation of TAG species within cells, enhance the breakdown of fats, and suppress the formation of lipids. Through this process, we identified flazine, an alkaloid beta-carboline featuring a carboxyl group at the C-3 position and a furfuryl alcohol group at the C-1 position, along with its piperidine C-ring derivative, extracted from oysters. These results suggest that extracts from plant-based foods and β-carboline alkaloids could play a significant role in preventing MAFLD. This implies that potential LDAI candidates can be found in nutraceuticals and functional foods, offering benefits for managing chronic conditions such as MAFLD/MASH. Consequently, in our previous study, we examined common vegetable extracts, including those from beans and garlic cultivated in Hokkaido, as potential functional food sources due to their ability to inhibit lipid accumulation, which results in reduced lipid droplet (LD) formation in cellular studies. Experiments conducted on hepatocytes revealed that bean extracts affect lipid droplet accumulation (LDA) and enhance lipid oxidation [24]. Furthermore, native bioactive Allium species from Hokkaido, Japan, have demonstrated the capacity to inhibit lipid accumulation in HepG2 cells, particularly AL1, AL3, and AL6. Our ongoing lipidomic study indicates that AL1, AL3, and AL6 could potentially reduce lipid accumulation and oxidative species in hepatocytes. Metabolomic analysis of AL1, AL3, and AL6 revealed notable differences in their metabolomes, which may explain the variations in their LDAI activities. The current study also employed lipidomic investigation, TG assays, metabolomic fingerprinting, and rapid dereplication of the chemical components in the identified bioactive extracts AL1, AL3, and AL6 using both 1D-NMR [^1^H, ^13^C, DEPT (90 and 135)] and LC-MS/MS techniques.

## 2. Materials and Methods

### 2.1. Chemical and Extraction

The supplementary information provides a comprehensive list of the chemicals and equipment utilized in this research, as previously reported [22,25,26] (Supplementary File S1). Oleic and linoleic acids were used in this study. The following reagents were used in the analysis of triglycerides (TGs): RIPA buffer from Nacalai Tesque (Kyoto, Japan), LabAssay Triglyceride from Waco Pure Chemical (Osaka, Japan), and Pierce BCA Assay Kit from Life Technologies (Carlsbad, CA, USA). For lipidomics analysis, EquiSPLASH LIPIDOMIX^®^ quantitative mass spectrometry internal standard, obtained from Avanti Polar Lipids (Alabaster, AL, USA), was used as the internal standard. The mobile phase for liquid chromatography/mass spectrometry (LC/MS) was composed of ammonium acetate (Wako Pure Chemical, Osaka, Japan) and LC-grade methanol (Kanto Chemical, Tokyo, Japan).

This research employed nine Allium specimens collected by D.F.D. and S.O. from Sapporo’s marketplace in April 2020 and maintained at the Health Innovation Center of the University’s Health Science Faculty (Supplementary File S1, Table S1, Figures S1).

### 2.2. Antioxidant Activity Index, Evaluation of Cell Viability, Lipid Droplet Accumulation Inhibition Assay, and TG Assay

The Antioxidant Activity Index (AAI) was assessed as previously described [27,28], following Scherer and Godoy’s method, which employs the DPPH radical assay. To conduct the test, a working solution was prepared by mixing 10 mg DPPH with 100 mL ethanol. Various extract concentrations, ranging from 6.25 to 200 μg/mL, were prepared through a series of two-fold dilutions. In a 96-well plate, 100 μL of each diluted extract was mixed with an equal volume of DPPH working solution. After incubation for 30 min at room temperature in the dark, the absorbance was measured at 517 nm. Vitamin C (ascorbic acid) and chlorogenic acid were used as the reference standards. The DPPH radical scavenging ability was calculated using the formula %RSA = [(Acontrol − Asample)/Acontrol] × 100, where A is the absorbance at 517 nm. According to the criteria set by Scherer and Godoy, antioxidant activity was categorized as poor if AAI < 0.5, moderate if AAI was between 0.5 and 1.0, strong if AAI ranged from 1.0 to 2.0, and very strong if AAI was greater than 2.0.

Tests for cytotoxicity and lipocytotoxicity were performed on HepG2 cells acquired from the RIKEN BRC Cell Bank located in Ibaraki, Japan, following the manufacturer’s protocols, as previously reported [22,25,26,27]. LDAI activity was assessed using an Oil Red O assay conducted in 24-well plates with four replicate treatments, following established procedures. The LD staining process was implemented, with modifications, based on previously published methodologies [22,25,26,27] (Supplementary File S1).

### 2.3. Lipidomic Analysis of Neutral Lipids: Analysis of the Accumulation of Triacylglycerols and Oxidized Hydroperoxide Species Using LC-MS

HepG2 cells were seeded in 35 mm culture dishes at 2.0 × 10^5^ cells/dish and incubated for 24 h. OA (0.25 mM) and test samples (150 and 300 μg/mL) were then added. The following day, LDs were extracted from cells and analyzed using LC-MS, following previously published methodologies [22,25,26,27], with some modifications (Supplementary File S1).

### 2.4. Metabolite Profiles of AL1, AL3, and AL6 Based on NMR and LC-MS/MS Analysis

Analysis of the metabolite profiles of AL1, AL3, and AL6 was conducted using nuclear magnetic resonance (NMR) and liquid chromatography–tandem mass spectrometry (LC-MS/MS) techniques. Detailed information about the methods and technical aspects of metabolite profile analysis using NMR and LC-MS/MS can be found in the Supplementary Materials (Supplementary File S1).

### 2.5. Rapid Dereplication of AL1 and AL6 Using 1D-NMR

A JOEL NMR spectrometer (ECX400, JEOL, Tokyo, Japan) was used for 1D-NMR experiments (^1^H-NMR, ^13^C-NMR, DEPT-135, and DEPT-90). The spectra were processed using JOEL v6.3.0 software, and chemical shifts (δH and δC) were recorded in ppm. The methanol extract (30 mg) was solubilized in 600 μL of CDCl_3._ ^13^C-NMR spectra measurements were taken at 100 MHz. JOEL software v6.3 (Delta NMR Processing and software) was employed for data processing, with calibration performed using solvent peaks at δC 77.16 ppm (CDCl_3_). The process involved manual phasing and baseline correction, followed by the alignment of DEPT experiments with the ^13^C spectra, using a specified δC value.

The SONAS lab at Université Angers (France) has developed MixONat v1.0.1, a free software for dereplicating natural product mixtures using ^13^C NMR. This program compares the δC values of natural products in mixtures to those in a specified database, taking multiplicities into account. It can be downloaded from http://sourceforge.net/projects/mixonat (accessed on 1 July 2024) [29]. Peak list and intensity data from experimental spectra (^13^C-NMR, DEPT-90, and 135) were exported as a reference.csv file, using Microsoft Excel (Microsoft 16.45, Redmond, WA, USA), and utilized in MixONat v1.0.1. This file contains a list of δCs in descending order, with corresponding intensities on the same line, separated by commas. The software processes these data using any dataset that provides molecular structures in the in-house structural databases DB1-3. Allium DB1 (1080 molecules), Amaryllidaceae DB2 (2020 molecules), and Euphrobacaeae DB3 (6286 molecules) databases contain ^13^C-NMR data for natural products extracted from the scientific literature in LOTUS DB. MixONat v1.0.1 generates compound proposals with scores ranging from 0 to 1 (0–100%), where 1 indicates a perfect match and 0 represents no similarity for a given compound in the database. A score exceeding 0.70 is deemed acceptable for tentative identification. Subsequently, the experimental data for the natural products with the highest scores are compared with data from the literature (Supplementary Materials).

## 3. Results and Discussion

### 3.1. Bioactive Lipid Droplet Accumulation Inhibitor (LDAI) Allium Extracts

**Figure 1:**
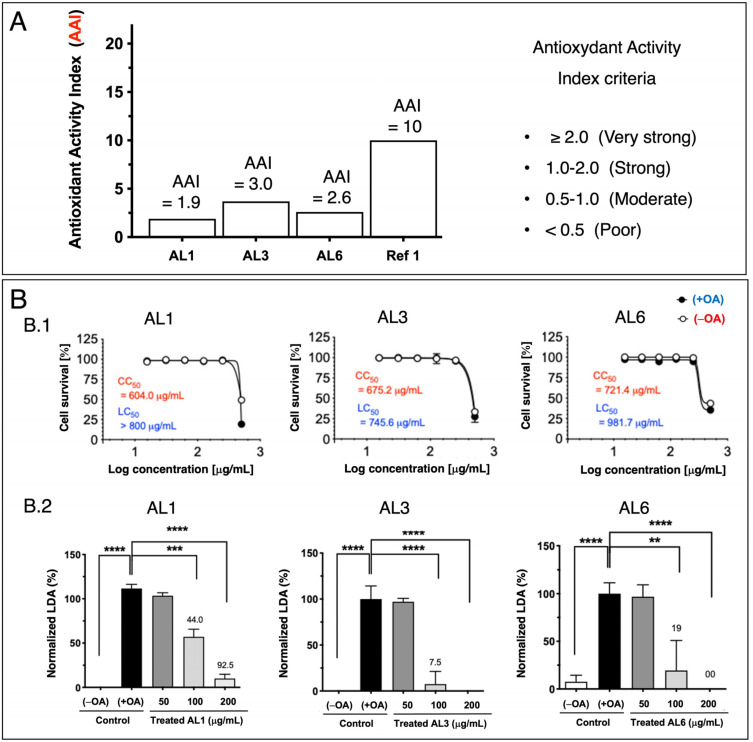
(**A**) Bioactive *Allium* extract (AL1, AL3, and AL6): Antioxidant activity index values for AL1, AL3, and AL6. A1. Graph showing AAI values. Individual AAI values for each sample. Activity criteria based on IC_50_ are classified as very strong, strong, moderate, and weak. (**B**) B.1: Cytotoxicity and lipocytotoxicity of AL1, AL3, and AL6 in HepG2 cells. B.2: The graph illustrates the mean LDAI values (four replicates) for AL1, AL3, and AL6 in OA-loaded HepG2 cells, demonstrating their LDAI activities. Statistical significance was assessed using one-way analysis of variance (ANOVA) with Tukey’s multiple comparison test against the untreated control, where **** *p* < 0.0001, *** *p* < 0.001, and ** *p* < 0.01.

**Figure 2:**
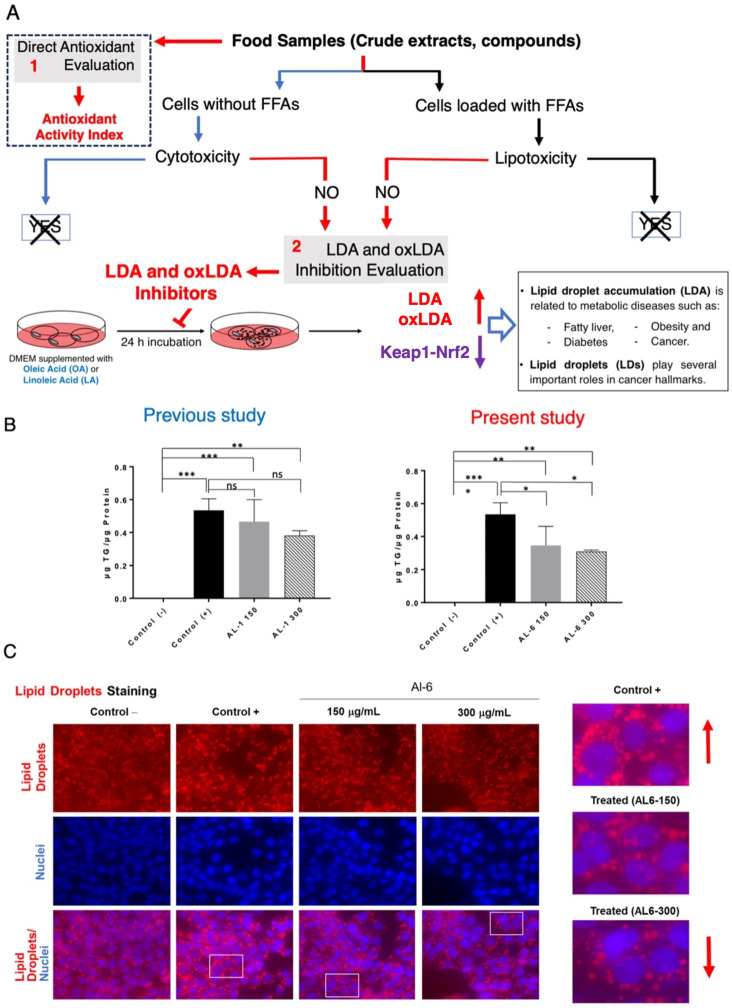
(**A**) Methodology for identifying food-derived antioxidants and substances that inhibit lipid droplet accumulation. (1) Evaluation of direct antioxidant properties and antioxidant activity index. (2) Assessment of LDAI/oxLDAI under conditions of fatty acid loading in relation to Keap1-Nrf2 pathway regulation. (**B**) Measurement of triglyceride levels in HepG2 cells after treatment with AL1 and AL6. Graph illustrating average LDA and LDAI values (six replicates). *** *p* < 0.001, ** *p* < 0.01, and * *p* < 0.05; one-way analysis of variance (ANOVA) with Tukey’s multiple comparisons test when contrasted with the control group without treatment (+OA). ns, not significant; TG, triglyceride; OA, oleic acid. (**C**) Visualization of cells treated with AL6 using fluorescence staining techniques. (Red arrow: in control indicated increased LD and in treated indicated decreased LD).

Previous studies have shown that nutraceuticals and functional foods, typically sourced from plants or their mixtures, can effectively combat metabolic disorders through LDAIs and antioxidants [22,25,26,27]. First, we report a non-cell assay for antioxidant activity that was measured using the DPPH assay. The antioxidant activity index (AAI) was categorized using the following criteria: IC_50_ ≥ 2.0, very high; 1.0–2.0, high; 0.5–0.1, moderate; and IC_50_ < 0.5, low. Among the AL samples tested (AL1–9), AL4 had the highest AAI, with AL3 and AL6 ranking second, followed by AL1, AL5, and AL7. Samples AL8 and AL9 exhibited weak antioxidant activities (Figure 1A). Cell viability was then assessed. The cytotoxic and lipocytotoxic effects of the ALs (AL1–9) on HepG2 cells were also assessed. The CC_50_ value, highlighted in red, indicates the concentration at which 50% of the cells died in DMEM without fatty acids (OA), reflecting cytotoxicity. Lipocytotoxicity is represented by the LC_50_ value, shown in blue, which signifies the concentration that resulted in 50% cell death in DMEM containing fatty acids (+OA). The CCK-8 assay was used to assess cytotoxicity and determine IC_50_ and LC_50_ values. Notably, none of the examined ALs (AL1–9) exhibited cytotoxicity or lipocytotoxicity [27]. Finally, this investigation explored LDAI and oxLDAI under nontoxic conditions, focusing specifically on the nontoxic levels of OA and LA. Research has shown that intracellular LD accumulation increases proportionally with oleic acid (OA) and linoleic acid (LA) concentrations [22,25,26,27]. Following these observations, the nine Allium extracts were simultaneously treated with OA or LA (0.25 mM) and incubated for a 24-h period. Notably, AL1, AL3, and AL6 showed marked reductions in LD, even at concentrations as low as 200 μg/mL (Figure 1(B.1,B.2)). Of the nine AL samples (AL1–9), three exhibited notable decreases in LD at 200 μg/mL, with LDAI values reaching 92.5, 93.5, and 81.0%, respectively, compared with the other extracts. A previous study reported the LDAI, oxidative lipidomics, and LC-MS/MS metabolome analysis of bioactive AL1 extracts [22,25,26,27]. The metabolomics analysis of LDAI of the bioactive extracts AL1, AL3, and AL6 suggested that AL1 and AL6 contain significantly different secondary metabolites that may impact the accumulation of neutral lipid TAGs and oxidized hydroperoxide TGOOH species. Consequently, this study was extended to AL6 extract for comprehensive metabolome analysis using a complementary NMR dereplication approach. Analysis of cell morphology changes induced by AL6 and AL1, TG assays, and lipidomic analysis of the accumulated neutral lipid TAG and oxidized TAG molecular species in LD were conducted. The AL6 extract-treated cells displayed a reduced size and number of lipid droplets compared with the control (those exposed to lipids alone) (Figure 2A,C). Dual staining of hepatocytes was performed using Oil Red O staining for lipid droplets and Hoechst staining for nuclei. The resulting images show lipid droplets in red, cell nuclei in blue, and a merged view at the bottom. At a concentration of 300 µg/mL, a dose-dependent inhibitory effect was observed, which was characterized by a decrease in the quantity and size of lipid droplets relative to the 150 µg/mL treatment. This finding indicated that AL6 hindered lipid droplet formation in a concentration-dependent manner during cellular fatty acid uptake (Figure 2A,C).

The concentration of triacylglycerol was determined by standardizing the TG kit measurements against the total protein content. Cells exposed to OA exhibited higher triacylglycerol levels than untreated cells. Furthermore, 150 μg/mL of extract induced a decrease in triacylglycerol content by approximately 13%, whereas 300 μg/mL of extract resulted in a 19% reduction. These results indicate that the decrease in triacylglycerol levels correlated with the concentration of the extract (Figure 2C).

### 3.2. Quantification of the Effects of AL1 and AL6 on Inhibition of the Accumulation of TAGs and TGOOH Species

Our recent study sought to address the shortcomings of traditional methods of examining oxidized lipids in LD lipidomes by investigating State-of-the-Art oxidative lipidomics techniques. Conventional approaches for studying oxidized lipids typically involve indirect detection of lipid oxidation products using colorimetric assays, immunological tests, electron spin resonance spectroscopy, or newer fluorescence-based techniques. Although they are frequently utilized to evaluate the extent of lipid oxidation, they may not always provide a comprehensive analysis of changes in lipid species [22,25,26,27]. Methods based on MS/MS for identifying oxidized molecular lipid species have demonstrated considerable promise [22,28,29,30] and were utilized in our current research. This transition to advanced MS techniques supplements the traditional imaging methods. MS/MS detection of oxidized molecular lipid species successfully tackles issues of sensitivity, specificity, accuracy, and dynamic range, while enabling rapid analysis. In comparison, alternative techniques may require modification and adaptation to assess lipid oxidation products. This approach is highly beneficial for the identification of oxidized lipids in complex mixtures with different concentrations and structural complexities, and enables efficient detection of the neutral oxidized lipid TG(OOH)n. The detection of oxidized lipids by MS is increasingly seen as a promising solution [22,25,26,27,30]. Nowadays, LC-MS techniques, especially LC-MS/MS, are used extensively in oxidative lipidomics applications due to their efficiency [22,25,26,27,30]. Consequently, our strategies employed LC-MS/MS analysis of the neutral lipidome in LD under OA and LA conditions.

MAFLD is characterized by the accumulation of lipids, mainly TAGs, in the liver. The progression of MAFLD, including MASH and related conditions, has been associated with oxidative stress, as evidenced by lipid oxidation byproducts [25,26,31,32,33,34,35,36]. This comparative investigation sought to analyze the changes in TAGs and oxidized TGOOH species in HepG2 cells exposed to AL1 and AL6, the active LDAI extracts, following treatment under two lipid loading scenarios: oleic acid (OA) and linoleic acid (LA). Lipidomic analysis of neutral lipid TAGs and oxidized TGOOH species was conducted using LC-MS/MS. The experiment was performed on HepG2 cells under both OA and LA conditions, followed by lipid droplet extraction. The results were normalized to the cell counts and internal standards.

In OA-treated control cells, 51 triacylglycerol molecular species were identified, whereas 53 were detected in LA-treated control cells. The research also noted triacylglycerol hydroperoxides at three distinct oxidation levels (TG (OOH) n = 3) (Supplementary File S1 and Tables S2–S5). In MASH liver models, significantly increased lipid peroxides are thought to play a role in MASH progression by impairing the structure and functionality of lipid-based biomembranes. LC-MS/MS analysis was employed to examine the differential effects of AL1 and AL6 on lipid hydroperoxides in HepG2 cells under OA and LA conditions. This method was used to assess changes in TAG-neutral lipids and TGOOH-oxidized species with the aim of elucidating how the chemical compositions of AL1 and AL6 affect specific aspects of lipid hydroperoxidation. A previously established oxidative lipidomic technique was used to conduct this comprehensive investigation [26,27].

#### 3.2.1. Comparative Analysis of AL1 and AL6 Inhibition of Accumulated TAGs and TGOOH Species Induced by Oleic Acid in Hepatocytes

**Figure 3:**
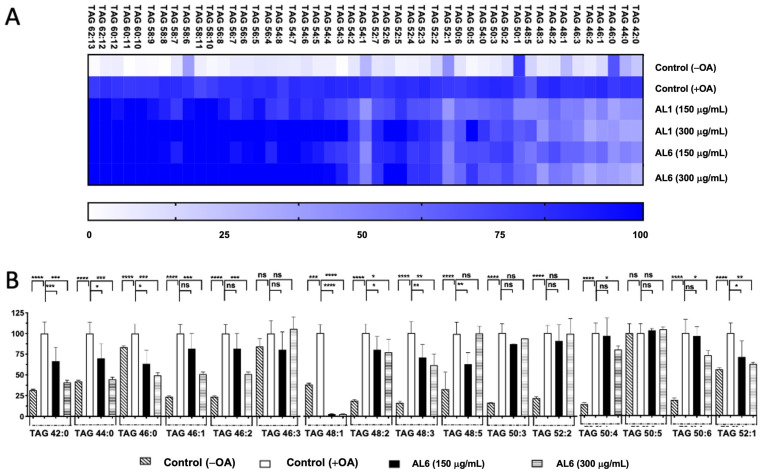
(**A**) Visualization of accumulated and suppressed TAG species in the cells using a heatmap. (**B**) Analysis of the variations in accumulated TAG species following OA administration. Examination of triacylglycerol molecular species after OA treatment with AL6. The graph displays the average values of LDA and LDAI (n = 6). Statistical significance: **** *p* < 0.0001, *** *p* < 0.001, ** *p* < 0.01, and * *p* < 0.05, determined by one-way ANOVA with Tukey’s post hoc test, comparing treated groups with the untreated control (+OA) group. ns indicates no significant difference; TAG, triacylglycerol; OA, oleic acid. *: Statistically significant and n.s.: not significant.

LC-MS metabolome analysis revealed both common metabolites and significant differences in certain compounds. Consequently, the roles of AL1 and AL6 in inhibiting the accumulation of TAGs and TGOOH species induced by oleic acid in liver cells were examined. As depicted in Figure 1(B.2), HepG2 cells exposed to OA showed a marked increase in LDA, with significant LDAI noted in the AL1, AL3, and AL6 samples. The impact of the LDAI bioactive Allium extracts AL1 and AL6 on TAG species accumulation in liver cells was examined using liquid chromatography–mass spectrometry (LC-MS). Orbitrap LC-MS was used to examine the profiles of neutral lipid TAG and TG(OOH) n = 3 species in the cells following a 24-h incubation period. This study identified all TAG and TGOOH species induced by OA (Supplementary File S1) Lipidomic analyses were conducted to determine the impact of OA loading on control cells and treated samples; we have previously reported the lipidomic analyses of AL1, and in this study, we extended our research to include AL6. Our findings revealed the presence of 51 triacylglycerol molecular species in OA loading condition with 9 TAGs reduced by AL1 and 11 TAGs reduced by AL6 (Figure 3, Table 1, and Supplementary File S1). The heatmap showed that the OA-treated cells contained numerous triacylglycerol molecular species, with TAG 42:0, TAG 44:0, TAG 46:0, TAG 46:1, and TAG 46:2 significantly inhibited by 1–50%. TAG 48:1 was completely suppressed by AL6, while TAG 48:2, TAG 48:3, TAG 50:4, TAG 50:4, TAG 50:6, and TAG 52:1 were significantly inhibited (51–75%).

**Figure 4:**
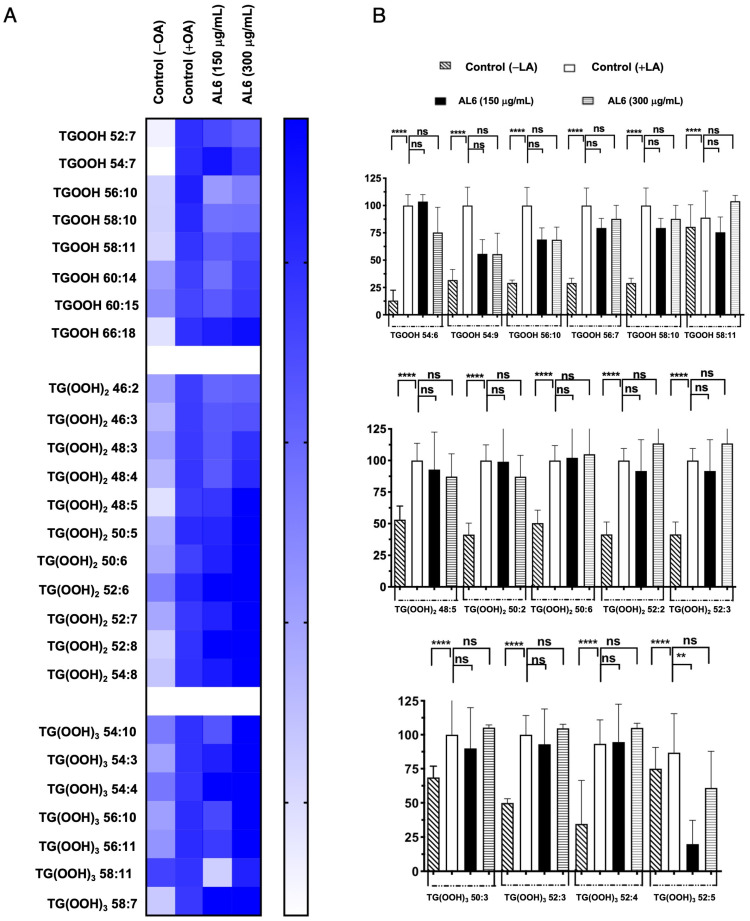
(**A**) Visualization of accumulated and inhibited TGOOH molecules within cells using a heatmap. (**B**) Measurement of fluctuations in the accumulated TGOOH molecules following OA exposure. Examination of TAG molecular species following OA treatment with AL6. Chart depicting average values of LDA and LDAI (six replicates): **** *p* < 0.0001 and ** *p* < 0.01, one-way analysis of variance (ANOVA) with Tukey’s multiple comparisons test, compared with the untreated control (+OA) group. ns, not significant; TGOOH, triacylglycerol hydroperoxide; OA, oleic acid. *: Statistically significant and n.s.: not significant.

Triacylglycerol hydroperoxides were also identified in addition to triacylglycerol molecules (Figure 4, Table 1, and Supplementary File S1). The accumulation rate in each cell group is depicted in a heatmap, with the accumulation in the fatty acid-treated control cells set as the baseline at 100%. Higher accumulation is indicated by darker colors. We detected eight TGOOH, eleven TG(-OOH)_2_, and seven TG(-OOH)_3_ species under OA-loaded conditions; among these, three TGOOH species (TGOOH 54:6, TGOOH 54:9, and TGOOH 56:10), two 2 TG(-OOH)_2_ species (TG(OOH)_2_ 48:5 and TG(OOH)_2_ 50:2), and one TG(-OOH)_3_ species (TG(OOH)_3_ 52:5) were suppressed by 51–75%, of 76–100%, and 51–75%, respectively, by AL6. The results demonstrated that treatment with AL-1 and AL6 under OA conditions led to a decrease in the accumulation of both triacylglycerol molecular species and triacylglycerol hydroperoxide, as well as a reduction of total accumulated TAG, with 17.6% for AL1 and 21.6% for AL6; total accumulated TGOOH, with 12.5% for AL1 and 37.5% for AL6; total accumulated TG(OOH)_2_, with 0% for AL1 and 18.1% for AL6; and total accumulated TG(OOH)_3_, with 28.6% for AL1 and 14.3% for AL6. These results indicate that AL6 reduced TAG 1.2 times more than AL1; AL6 reduced TGOOH 3 times more than AL1; and only AL6 reduced TG(OOH)_2_, with no reduction observed for AL1. Meanwhile, AL1 reduced TG(OOH)_3_ 2 times more than AL1, suggesting that AL6 reduced more triacylglycerol and triacylglycerol hydroperoxide TGOOH and TG(OOH)_2_ in OA condition than AL1 (Table 1). These findings confirmed that AL1 and AL6 treatments reduced the accumulation of both triacylglycerol molecular species and triacylglycerol hydroperoxides, with AL6 being more effective at reducing the accumulation of triacylglycerol hydroperoxide TGOOH and TG(-OOH)_2_ (Figure 3 and Figure 4).

#### 3.2.2. Comparative Analysis of AL1 and AL6 Inhibition of Accumulated TAG and TGOOH Species Induced by Linoleic Acid in Hepatocytes

**Figure 5:**
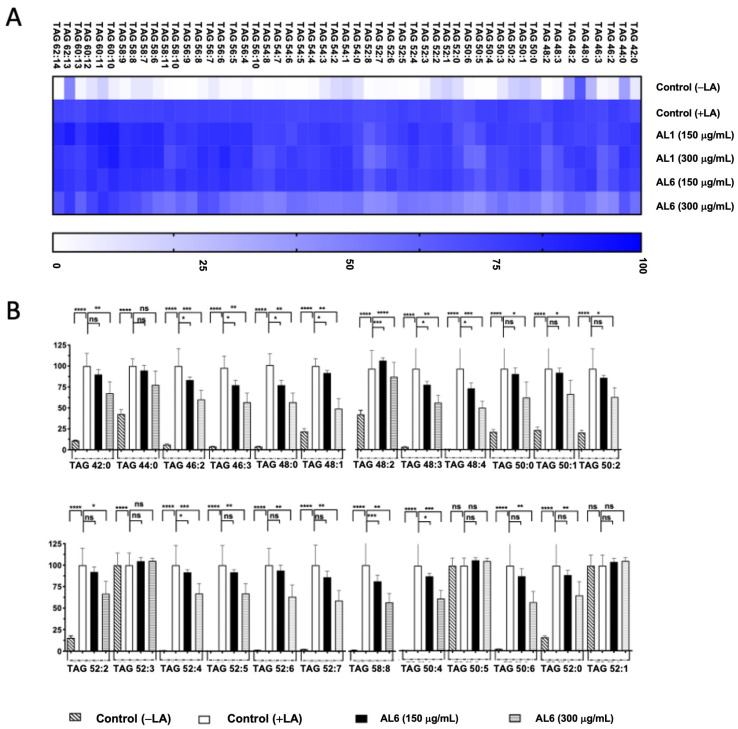
(**A**) Heatmap depicting accumulated and inhibited TAG species in cells. (**B**) Measurement of the variation in accumulated TAG species following LA treatment. Examination of triacylglycerol molecular species after LA treatment with AL6. The graph displays average values for LDA and LDAI (six replicates): **** *p* < 0.0001, *** *p* < 0.001, ** *p* < 0.01, and * *p* < 0.05, one-way ANOVA with Tukey’s multiple comparisons test. Comparisons were made against the untreated control (+LA) group. ns, not significant; TAG, triacylglycerol; LA, linoleic acid. *: Statistically significant and n.s.: not significant.

In our earlier study, we found that LA induced LDA and oxLDs in liver cells. Building on these results, we carried out a comparative study of HepG2 cells treated with both OA and LA. Our prior examination of AL1-LDAI extract indicated a decrease in several TAG and TGOOH molecules in the presence of LA. This investigation aimed to thoroughly explore the changes in TAG and TGOOH accumulation caused by the Allium AL6-LDAI extract, utilizing LC-MS techniques, and comparing them with previously reported AL1. The sophisticated Orbitrap LC-MS method revealed the accumulation of TAG and TGOOH molecules induced by LA. This advanced lipidomic approach was employed to assess and quantify the buildup and reduction of intracellular TAG and TGOOH. LC-MS analysis of liver cells exposed to LA revealed 53 accumulated triacylglycerol molecular species. Among the 53 TAGs accumulated under OA conditions, 6 TAGs were reduced by AL1, and 20 TAGs were reduced by AL6 (Figure 5; Table 2).

**Figure 6:**
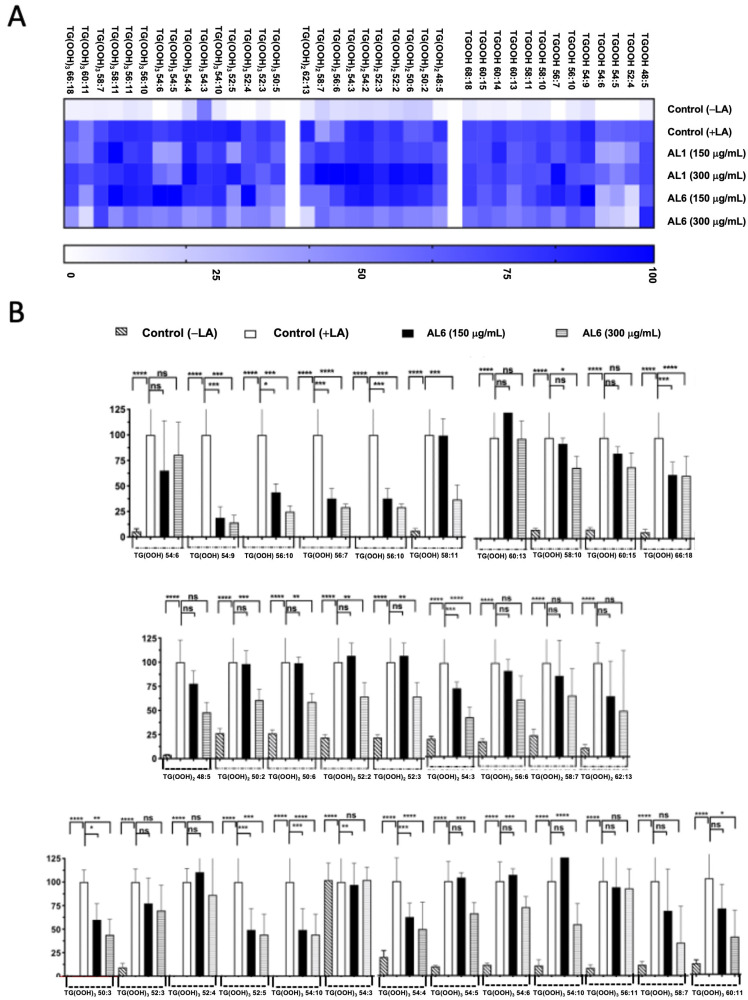
(**A**) Heatmap depicting accumulated and inhibited TGOOH species within cells. (**B**) Measurement of fluctuations in accumulated TG(OOH) n = 3 species when exposed to LA treatment. Examination of TG(OOH) n = 3 molecular species under LA treatment with AL6. The chart illustrates average values of LDA and LDAI (six replicates). **** *p* < 0.0001, *** *p* < 0.001, ** *p* < 0.01, and * *p* < 0.05, using one-way analysis of variance (ANOVA) with Tukey’s multiple comparisons test in relation to the untreated control (+OA) group. ns, not significant; TGOOH, triacylglycerol hydroperoxide; LA, oleic acid. *: Statistically significant and n.s.: not significant.

Triacylglycerol hydroperoxide was detected under LA conditions. To better understand the inhibition of the accumulated lipid species, omics quantification was performed. A heat map was created for further analysis, showing the accumulation rate for each cell group, with darker colors representing higher accumulation levels. The heatmap indicates that LA treatment led to the accumulation of the oxidized form, TAG hydroperoxide (TGOOH), in hepatocytes. This study further explored how TGOOH species change under LA conditions after exposure to the bioactive compound Allium AL6 compared to AL1. To provide a more detailed understanding of the hydroperoxide content of TAG molecular species, a heat map was generated (Figure 6). This visual representation shows the accumulation rate for each cell group, with darker shades indicating higher levels of accumulation. LC-MS-based analysis revealed that the cells treated with LA contained various triacylglycerol molecular species. Among these, TAG 42:0, TAG 46:2, TAG 46:3, TAG 48:0, TAG 48:3, TAG 50:1, and TAG 50:2 were significantly inhibited by 51–75%, whereas TAG 48:1 and TAG 48:4 were inhibited by 0–50% when exposed to AL6. Under LA loading conditions, this study clearly identified changes in both minor and major accumulated lipid molecular species of TAG and TGOOH following treatment with AL6 and AL1 LDAI bioactive food extracts. Approximately 38 accumulated TG(OOH)n species were detected under LA conditions. When LA was loaded, 13 TGOOH, ten TG(-OOH) 2, and 15 TG(-OOH) 3 species were observed (Figure 6 and Table 2). These observations were made possible by the presence of oxidized molecular lipid species. Under LA conditions, 13 TGOOHs were detected in LA instead of 8 TGOOHs in OA, with 6 TGOOHs reduced by AL1 and 10 TGOOHs reduced by AL6; 10 TG(OOH)_2_ species were detected in LA instead of 11 TG(OOH)_2_ species in OA, with 1 TG(OOH) reduced by AL1 and 8 TG(OOH)_2_ species reduced by AL6; and 15 TG(OOH)s were detected in LA instead of 7 TG(OOH)_2_ species in OA, with 6 TG(OOH)_3_ species reduced by AL1 and 9 TG(OOH)_3_ species reduced by AL6 (Table 2). Among these, ten TGOOHs, including TGOOH 54:9, TGOOH 56:10, TGOOH 56:7, TGOOH 58:10, and TGOOH 58:11, were inhibited by 0–50%; eight TG(-OOH)_2_ species, including TG(OOH)_2_ 54:3 and TG(OOH)_2_ 62:13, were inhibited by 0–50%, while TG(OOH)_2_ 50:2, TG(OOH)_2_ 50:6, TG(OOH)_2_ 52:2, TG(OOH)_2_ 52:3, TG(OOH)_2_ 56:6, and TG(OOH)_2_ 58:7 were inhibited by 50–75%; and nine TG(-OOH)_3_ species, including TG(OOH)_3_ 50:3, TG(OOH)_3_ 52:5, TG(OOH)_3_ 54:10, TG(OOH)_3_ 54:4, TG(OOH)_3_ 56:10, TG(OOH)_3_ 58:7, and TG(OOH)_3_ 60:11, were inhibited by 0–50%, while TG(OOH)_3_ 54:5 and TG(OOH)_3_ 54:6 were inhibited by 51–75% by AL6 treatment. The results demonstrated that treatment with AL-1 and AL6 under LA condition led to a decrease in the accumulation of both triacylglycerol molecular species and triacylglycerol hydroperoxide, as well as a reduction of total accumulated TAG, with 11.3% for AL1 and 37.7% for AL6; total accumulated TGOOH, with 46.1% for AL1 and 77.0% for AL6; total accumulated TG(OOH)_2_, with 10% for AL1 and 80% for AL6; and total accumulated TG(OOH)_3_, with 40% for AL1 and 60% for AL6. These results indicate that AL6 reduced TAG 3.3 times more than AL1, AL6 reduced TGOOH 1.7 times more than AL1, AL6 reduced TG(OOH)_2_ 8 times more than AL1, and AL6 reduced TG(OOH)_3_ 1.5 times more than AL1, suggesting that AL6 reduced more triacylglycerol and triacylglycerol hydroperoxide TGOOH, TG(OOH)_2_, and TG(OOH)_3_ in LA condition than AL1.

The results demonstrated that treatment with AL1 and AL6 led to a decrease in the accumulation of both triacylglycerol lipid species and triacylglycerol hydroperoxides, with AL6 reducing more triacylglycerol hydroperoxides (TGOOH, TG(OOH)_2_, and TG(OOH)_3_) under the LA condition (Table 2).

These findings suggest that the Allium antioxidant sample AL6 has the potential to control TAG and TGOOH accumulation in lipid droplets induced by free fatty acids.

#### 3.2.3. Comparative Analysis of Hydroperoxide Lipid Species Inhibited in OA and LA by AL6

Various fatty acids are readily transformed into TAGs. Under certain oxidative stress conditions, TAGs are oxidized to form triacylglycerol hydroperoxide (TGOOH). Hydroperoxidation can occur at different levels in unsaturated TAG molecules, such as oleic acid (C18:1) and linoleic acid (C18:2) [1,22,25,26,27]. In this study, OA and LA were used as unsaturated FFAs to produce TAGs in LD in HepG2 cells with C18:1 and C18:2 acyl chains, and to reduce the possibility of acyl group composition in the accumulated and oxidized lipid species produced in the lipid droplets. TAG and TG(OOH)n lipid profiles were investigated based on the basis of their sum composition of the total carbon and the number of double bonds on the species with their corresponding mass [22,25,26,27]. We then built and extended our TAG-DB (local database) using reported molecular lipid species from mainly LIPID MAPS and available sources to reach 6960 TAGs containing the composition of molecular lipid species by indicating the corresponding acyl chains and number of their isomers. The species filter for TAG and hydroperoxide lipid species in this study contained at least one C18:2 or C18:1 with an OOH group in the lipid composition of the inhibited species. Oxidation may occur in other molecular species that were filtered and were not considered in the present study. The comparison of TGOOH lipid species inhibited by AL6 in both OA and LA revealed that TGOOH 54:9, 56:7, 58:10, and 58:11 were significantly inhibited, whereas TGOOH 54:6, 54:9, and 56:10 (Supplementary File S2) suggested that one common TG(OOH)_2_ 54:9 lipid species may be inhibited for both OA- and LA-treated cells. However, the main differences could be attributed to their fatty acyl lipid chains. In the TGOOH 54:9 from OA, among the 27 known TAGs, only 3 contained at least one C18:1 acyl chain (TAG 18:1,18:4,18:4 with 3 isomers: LMGL03013034 and TAG 18:4,18:1,18:4: LMGL03016901), and in the TGOOH 54:9 from LA, among 27 known TAGs, only 2 contained at least one C18:1 acyl chain (TAG 14:1,18:2,22:6 and TAG 18:2,18:3,18:4). Comparison of TG (OOH)_2_ lipid species inhibited by AL6 in both OA and LA revealed that TG(OOH)_2_ 54:3 and 52:13 were significantly inhibited in OA, whereas TGOOH 48:5 and 50:2 in OA suggested that no common TG(OOH)_2_ may be inhibited. They have different fatty acyl lipid chains under both the conditions (Supplementary File S2).

A comparison of the TG (OOH)_3_ lipid species inhibited by AL6 in both OA and LA revealed that TG(OOH)_3_ 52:5 was significantly inhibited in OA, whereas TGOOH 52:5, 50:3, 54:10, 54:4, 56:10, 58:7, and 60:11 were significantly inhibited in LA, suggesting that TGOOH 52:5 may be present in both OA- and LA-treated cells. Nonetheless, the primary distinction likely stems from the composition of the fatty acyl lipid chains. Among the 72 known TAGs in OA, only 7 contained at least one C18:1 acyl chain (TAG 17:2_17:2_18:1, TAG 16:1_18:1_18:3, TAG 12:0_18:1_22:4, TAG 14:0_18:1_20:4, TAG 14:1_18:1_20:3, TAG 16:0_18:1_18:4, and TAG 16:1_18:1_18:3), all with 6 isomers each (LMGL03010140, LMGL03010168, LMGL03013479, LMGL03014372, LMGL03014777, and LMGL03015818) from lipid maps; and in TGOOH 52:5 from LA, among 60 known TAGs, only 7 contained at least one C18:2 acyl chain (TAG 17:1_17:2_18:2 and TAG 16:1_18:2_18:2 with 3 isomers; and TAG 16:0_18:2_18:3, TAG 12:0_18:2_22:3, TG 14:0_18:2_20:3, TAG 14:1_18:2_20:2, and TAG 14:1_18:2_20:2 with 6 isomers, with the following LD codes from lipid maps, respectively: LMGL03010144, LMGL03010163, LMGL03010167, LMGL03013497, LMGL03014390, LMGL03014795 and LMGL03015731) (Supplementary File S2). The analysis indicated that the potential molecular species might be completely different, suggesting that FFA loading conditions play a crucial role and are an essential target strategy for obtaining a complete picture of deregulation and inhibition. To gain a more comprehensive understanding of the lipid species involved in accumulation and inhibition under different FFA conditions, further investigation is needed into the current strategy and outcomes by introducing a load-labeled FFA for the synthesis of labeled TAG molecular species in the LD of cells for more precise identification, characterization, and investigation of OOH location in multi-oxidation patterns.

This study indicates that foods derived from Allium may inhibit LD formation by decreasing intracellular lipids and lipid hydroperoxides within hepatocytes. This approach enables sensitive and precise quantification, clearly distinguishing between OA and LA loading conditions. Expanding these findings to include various FFAs and their combinations may provide further understanding of oxidative stress mechanisms and diagnosis through LOOH molecular species. This could potentially enhance our knowledge of MAFLD/MASH prevention and identification of drug targets for related metabolic disorders. Therefore, this study has extended the examination of bioactive AL6 not only by assessing changes in TAG and TGOOH molecules under OA and LA conditions, but also by investigating the metabolite search and rapid dereplication of chemical constituents in this bioactive extract using MS/MS and NMR approaches.

### 3.3. Metabolite Fingerprinting and Rapid Dereplication of Key Compounds in Bioactive Extracts

Metabolomic analysis has enabled the rapid identification of complex mixtures of organic compounds, including plant extracts. In the last few years, LC-MS, GC-MS, and NMR have become crucial techniques for directly identifying natural products within complex plant matrices. Over the past ten years, automated dereplication processes utilizing ^13^C-NMR have emerged as powerful tools for pinpointing key compounds in mixtures. These processes use moderate-field instruments (400 MHz), readily available automation procedures, and specialized software. Although MS offers greater sensitivity, ^13^C-NMR and DEPT (135 and 90) are particularly useful for differentiating overlapping diastereomers and metabolites in ^1^H-NMR spectra. This study combined LC-MS/MS techniques with [^1^H, ^13^C, DEPT (135 and 90)] NMR and 1D-NMR methodologies to rapidly profile and identify potential chemical components in natural extract mixtures.

#### 3.3.1. LC-MS/MS Analysis and Global Natural Product Social-Assisted Dereplication of AL1, AL3, and AL6 Constituents

Analysis and identification of AL1, AL3, and AL6 were conducted using Global Natural Product social-assisted LC-MS/MS in the positive ionization mode. High-resolution mass spectrometry was used to generate tandem mass spectra, which were then examined and clustered to establish molecular networks and compound annotation databases. In the molecular network, the nodes represent metabolites with similar chemical properties. The network displayed nodes indicating the parent ion of each analyzed AL sample (Supplementary Figure S2). LC-MS Orbitrap was utilized for metabolite screening of the LC-MS/MS data from AL1, AL3, and AL6, with the results presented as 3D images obtained through DFF (Supplementary File S1 and Figures S3–S5).

Analysis of bioactive AL6 identified 10 parent ions, with carbohydrates and glucosides as the primary compounds, as evidenced by the LC-MS/MS and 3D data (Supplementary Figures S3–S5). Further investigation revealed minor components, including organic acids, amino acids, and organosulfur compounds (Supplementary Figures S3–S5). Several metabolites were identified in the AL extracts (130), with 18 components (118) present in AL1, AL3, and AL6. The GNPS molecular clusters generated from the AL1 and AL6 extracts exhibited shared ionic parents **4**, **7**, **8**, and **9**. The unique ionic parent 1518 in AL6 was identified using LC-MSMS data and MN analysis.

Four ion parents—360.1461, MS1 (**4**); 104.1058, MS1 (**7**); 356.3483, MS1 (**9**); and 391.2800, MS1 (**10**)—were identified in all three bioactive extracts; in contrast, ion parent 282.2759, MS1 (**13**), was detected in AL3 and AL6 only. Each extract also showed distinct parent ions: AL1 contained 435.4147, MS1 (1); 424.4102, MS1 (**2**); 291.0977, MS1 (**3**); 116.0693, MS1 (**5**); 258.1073, MS1 (**6**); 433.3988, MS1 (**11**); and 452.4411, MS1 (**12**). AL3 contained 338.3378, MS1 (**14**), while AL6 contained 198.0951, MS1 (**15**); 180.0847, MS1 (**16**); 203.0504, MS1 (**17**); and 219.0242 MS1 (**18**) (Supplementary File S1 and Figures S2–S5). The bioactive extracts AL1 and AL6 were subjected to 1D-NMR analyses as complementary techniques.

#### 3.3.2. Comparison of 1D Nuclear Magnetic Resonance Profiling and Fingerprinting of AL1 and AL6

**Figure 7:**
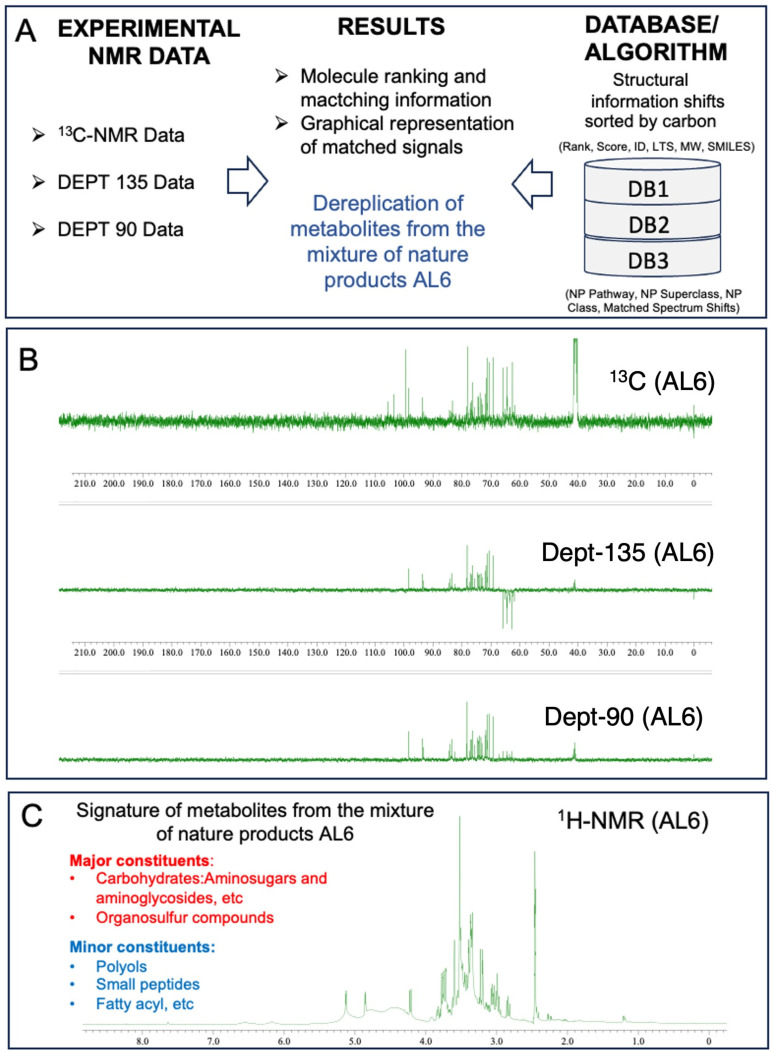
Fingerprinting and profiling of potential metabolites in AL6 food extract, using 1D-NMR. (**A**) Overall operation of the MixONat program. This program matches the chemical shifts of the AL6 experiment (13C, optionally DEPT-135 and DEPT-90) with those of the DB1-3 databases (*Allium* DB1 (1080 molecules), *Amaryllidaceae* DB2 (2020 molecules), and *Euphrobacaeae* DB3 (6286 molecules), which contain molecular δC selected from the LOTUS database. (**B**) ^13^C-NMR and DEPT (135 and 90) spectra of AL6 in DMSO, with *δ*_C_ ranging from 0 to 190 ppm. (**C**) ^1^H-NMR profile of AL6 in DMSO, with *δ*_H_ ranging from 0 to 12.0 ppm. The spectra were processed using JOEL software, and the chemical shift (*δ*) values are expressed in ppm.

Analysis of AL1 and AL6 was conducted using 1D nuclear magnetic resonance profiling and fingerprinting techniques. This study employed ^1^H-NMR profiling and ^13^C-NMR metabolite dereplication to analyze AL1. The ^1^H-NMR spectra are shown in Figure 7A. AL1′s ^1^H-NMR spectrum exhibited a notable chemical shift between 6 and 9 ppm, indicating the presence of carbohydrate components. For AL6, ^1^H-NMR metabolite profiling and fingerprinting revealed signatures linked to carbohydrates and glucosidic metabolites, with small amounts of polyols, small peptides, amino acids, and organosulfur compounds (Figure 7). The ^1^H-NMR profiles revealed carbohydrate groups with chemical shifts in the range of 2.5 to 6 ppm. Previous research has shown that *Allium* species contain various components, with saponin derivatives being prominent, primarily consisting of organosulfur compound derivatives. The presence of carbohydrates and organosulfur compounds in the extracts was confirmed using ^13^C-NMR data. Metabolome analysis for chemical composition screening of the AL6 methanolic extract was performed using ^13^C-NMR and DEPT (135 and 90) (Figure 7B). The in-house structural databases—*Allium* DB1 (1080 molecules), *Amaryllidaceae* DB2 (2020 molecules), and *Euphrobacaeae* DB3 (6286 molecules)—containing ^13^C-NMR data for natural products extracted from the scientific literature in LOTUS DB were analyzed using MixONat (Table 3). MixONat generates compound matches with scores ranging from 0 to 1 (0–100%), where 1 indicates a perfect match and 0 represents an absence of similarity for a given compound in the database. A score above 0.70 is considered acceptable for tentative identification. Next, experimental data from natural products with the highest scores were compared with data from the literature. The initial 50 compounds of each DB (DB1, rank1–50 (S**1**–**50**); DB2, rank1–50 (S**51**–**100**); and DB3, rank1–50 (S**101**–**150**) were identified based on ^13^C-NMR chemical shifts and intensities, which indicate metabolite abundance (Table 3). These substances were categorized by comparing them to the 1080 natural products in *Allium* DB1, the 1080 natural products in Amaryllidaceae DB2, and their spectroscopic data in the databases. The dereplication process revealed various fundamental organosulfur compounds, carbohydrates, and glucosidic metabolites, with small amounts of organic acids and amino acids with different scores (Supplementary File S1 and Figures S6–S22).

Dereplication from *Allium* DB1 (ID: 1-1080 molecules) identified six molecules with a score of 1.0: (S**1**): Rank: 1, ID: 580, LTS0155285, MW: 92.09, Score: 1.0 (3/3 carbons); (S**2**): Rank: 2, LTS0272557, MW: 342.3, Score: 1.0 (12/12 carbons); (S**3**): Rank: 3, ID: 269, LTS0013597, MW: 180.16, Score: 1.0 (6/6 carbons); (S**4**): Rank: 4, ID: 585, LTS0199986, MW: 182.17, Score: 1.0 (6/6 carbons); (S**5**): Rank: 5, ID: 535, LTS0150163, MW: 122.12, Score: 1.0 (4/4 carbons); (S**6**): Rank: 6, ID: 292, LTS0113066, MW: 504.44, Score: 1.0 (18/18 carbons). Dereplication from *Allium* DB1 (1080 molecules) identified six molecules with scores ranging from 0.70 to 0.99 (Supplementary File S1 and Figures S6–S22, Table 3).

Dereplication from *Amaryllidaceae* DB2 (ID: 1-2020 molecules) identified six molecules with a score of 1.0: (S**51**): Rank: 1, ID: 946, LTS0155285, MW: 92.09, Score: 1.0 (3/3 carbons); (S**52**): Rank: 2, ID: 902, LTS0272557, MW: 342.3, Score: 1.0 (12/12 carbons); (S**53**): Rank: 3, ID: 1677, LTS0013597, MW: 180.16, Score: 1.0 (6/6 carbons; (S54): Rank: 4, ID: 954, LTS0199986, MW: 182.17, Score: 1.0 (6/6 carbons); (S55): Rank: 5, ID: 845, LTS0150163, MW: 122.12, Score: 1.0 (4/4 carbons); (S**56**): Rank: 6, ID: 1866, LTS0113066, MW: 504.44, Score: 1.0 (18/18 carbons). Dereplication from *Allium* DB1 (1080 molecules) identified six molecules with scores ranging from 0.70 to 0.99 (Supplementary File S1 and Figures S6–S22, Table 3).

Dereplication from *Euphrobacaeae* DB3 (ID: 1-6286 molecules) identified twenty three molecules with a score of 1.0; the top ten are presented as follows: (S**101**): Rank: 1, ID: 1024, LTS0087699, MW: 152.15, Score: 1.0 (5/5 carbons); (S**102**): Rank: 2, ID: 5220, LTS0023185, MW: 152.15 Score: 1.0 (5/5 carbons); (S1033): Rank: 3, ID: 4101, LTS0231470, MW: 193.2, Score: 1.0 (7/7 carbons); (S**104**): Rank: 4, ID: 2371, LTS0161003, MW: 193.2, Score: 1.0 (7/7 carbons); (S105): Rank: 5, ID: 2608, LTS0087238, MW: 193.2, Score: 1.0 (7/7 carbons); (S**106**): Rank: 6, ID: 3875, LTS0263530, MW: 193.2, Score: 1.0 (7/7 carbons); (S107): Rank: 7, ID: 1408, LTS0107522, MW: 180.16, Score: 1.0 (6/6 carbons); (S**108**): Rank: 8, ID: 1787, LTS0204783, MW: 193.2, Score: 1.0 (7/7 carbons); (S**109**): Rank: 9, ID: 122, LTS0132398, MW: 180.16, Score: 1.0 (6/6 carbons); (S**110**): Rank: 10, ID: 1831, LTS0272557, MW: 342.3, Score: 1.0 (12/12 carbons). Dereplication from *Allium* DB1 (1080 molecules) identified six molecules, with scores ranging from 0.70 to 0.99 (Supplementary File S1 and Figures S6–S22, Table 3).

Next, experimental data from natural products with the highest scores are compared with data from the literature. The initial 50 compounds in each DB (DB1: rank1–50 (S**1**–**50**), DB2: rank1–50 (S**51**–**100**), and DB3: rank1–50 (S**101**–**150**)) were identified based on ^13^C-NMR chemical shifts and intensities, which indicate metabolite abundance. These substances were categorized by comparing them to the 1080 natural products in *Allium* DB1, the 1080 natural products in *Amaryllidaceae* DB2, and their spectroscopic data in the databases.

In addition, to identify metabolites not previously reported in DB1-3, the dereplication process was extended to the newly built *in-house* NP Superclass DBs of carbohydrates (DB-4, ID: 2685 molecules), identified as one of the major metabolites, and small peptides (DB-5, ID: 1913 molecules), identified as one of the minor metabolites. The dereplication process for D4 and D5 revealed several carbohydrates and small peptides, respectively, not reported in DB1-3. Dereplication from carbohydrates DB-4 (ID: 2685 molecules) identified 135 molecules with a score of 1.0. Seven replicated molecules chosen at random are provided here: (1) Rank: 1, ID: 1043, LTS0087699, MW: 152.15, Score: 1.0 (5/5 carbons), Matched ^13^C spectrum shifts, *δ*_C_: 63.49 63.59 73.80 74.30 74.51; (2) Rank: 7, ID: 2269, LTS0224479, MW: 179.17, Score: 1.0 (6/6 carbons), Matched ^13^C spectrum shifts, *δ*_C_ 61.91 63.49 71.71 72.01 73.08 84.35; (3) Rank: 38, ID: 1138, LTS0107522, MW: 180.16, Score: 1.0 (6/6 carbons), Matched ^13^C spectrum shifts, *δ*_C_: 61.91 70.63 72.01 73.08 73.41 93.67, Rank: 53, ID: 357, LTS0271376, MW: 254.23, Score: 1.0 (9/9 carbons), Matched ^13^C spectrum shifts, *δ*_C_: 62.64 63.49 69.21 71.71 72.01 73.80 76.25 77.22 103.43; (5) Rank: 57, ID: 792, LTS0132398, MW: 180.16, Score: 1.0 (6/6 carbons), Matched ^13^C spectrum shifts, *δ*_C_: 61.91 70.63 72.01 73.08 73.41 93.67; (6) Rank: 73, ID: 915, LTS0084687, MW: 344.31, Score: 1.0 (12/12 carbons), Matched ^13^C spectrum shifts, *δ*_C_: 61.91 62.64 64.35 70.63 71.26 73.08 73.41 74.30 76.25 76.75 77.11 103.43; (7) Rank: 106, ID: 1731, LTS0269408, MW: 355.34, Score: 1.0 (13/13 carbons), Matched ^13^C spectrum shifts, *δ*_C_: 61.91 62.64 63.49 63.59 69.21 71.71 72.01 73.08 73.41 73.80 77.22 78.46 103.43. A total of 125 molecules were observed with a score of 1.0–0.90, and 250 molecules were observed with a score of 0.9–0.80. Dereplication from small peptides DB-5 (ID: 1913 molecules) identified seven molecules with a score of 1.0–0.90, 10 molecules with a score of 0.9–0.80, and 43 molecules with a score of 0.8–0.70. This result suggests that several metabolites identified in the newly built *in-house* NP Superclasses DB4 and DB5 have not been reported in the family DB1-3. The investigation using Superclass DB NPs could therefore enable the in-depth discovery of several secondary metabolites in bioactive matrices.

Consistent with the NMR findings, distinct peaks were identified in the HPLC chromatogram of AL6. Following this, HPLC and LC-MS analyses were conducted on the chosen *allium* samples, revealing notable peaks for AL6 compared with AL1–5 and 7–9. Consequently, a comprehensive LC-MS/MS examination of AL6 components was performed using diagnostic fragmentation filtering (DFF) and Global Natural Product Social-aided dereplication, which indicated the presence of unidentified ion parents (1–4, 11, and 12). The MS fragmentation characteristics of these unidentified ion parents suggest the presence of organosulfur compounds, carbohydrates, and glucosidic metabolites in AL6. The metabolome of the bioactive AL6 extract was analyzed using NMR, LC-LTQ-MS-MS, and MN techniques, resulting in tentative identification and rapid profiling of organosulfur compounds, carbohydrates, and glucosidic metabolites, with small amounts of organic acids and amino acids with different scores. This profile is believed to represent the chemical metabolite signature of the bioactive AL6 extract (Supplementary Figures S6–S25).

## 4. Conclusions

This study investigated the effects of *Allium* extracts on lipid droplet formation in HepG2 cells exposed to free fatty acids. The results demonstrated that AL1, AL3, and AL6 decreased lipid droplet buildup in liver cells. Based on comparative lipidomic analysis, AL1 and AL6 were shown to suppress the accumulation of TAG and TGOOH in hepatocytes, with this suppression measured under both OA and LA conditions. AL1 primarily consists of carbohydrates and iridoid glucosides, with smaller amounts of organic acids, amino acids, and organosulfur compounds, while AL6 consists of carbohydrates, glucosidic metabolites, and organosulfur compounds, with small amounts of polyols, fatty acyls, small peptides, and amino acids with different scores. These components are promising candidates for the development of regulators of lipid droplet accumulation and oxidized lipid droplets. Dietary compounds may serve as therapeutic options, but additional in vivo and human research is necessary. The findings of this study indicate that Allium might be an effective dietary option for preventing excessive lipid droplet buildup, and could potentially be a valuable resource for pharmaceutical research and development

## Figures and Tables

**Table 1:** Comparison of accumulated TAG and TGOOH species under OA conditions and the inhibiting effect induced by AL1 and AL6.

OA	Neutral Lipid Species							
	AL1	AL6	AL1	AL6	AL1	AL6	AL1	AL6
	TAG	TAG	TGOOH	TGOOH	TG(OOH)_2_	TG(OOH)_2_	TG(OOH)_3_	TG(OOH)_3_
Number of accumulated	51	51	8	8	11	11	7	7
Number of inhibited	9	11	1	3	0	2	2	1

**Table 2:** Comparison of accumulated TAG and TGOOH species under LA conditions and the inhibiting effect induced by AL1 and AL6.

LA	Neutral Lipid Species							
	AL1	AL6	AL1	AL6	AL1	AL6	AL1	AL6
	TAG	TAG	TGOOH	TGOOH	TG(OOH)_2_	TG(OOH)_2_	TG(OOH)_3_	TG(OOH)_3_
Number of accumulated	53	53	(8)13	(8)13	(11)10	(11)10	(7)15	(7)15
Number of inhibited	6	20	6	10	1	8	6	9

**Table 3:** Dereplicated metabolites from AL6 using DB1-3: Number of major dereplicated compounds scored.

Databases	Scores				Profile Metabolites	Top Dereplicated
	1	1–0.90	0.89–0.80	0.79–0.70	1–0.70	Compounds
DB1	6	3	16	3	24	**50** in 1080
DB2	6	1	16	6	28	**50** in 2020
DB3	23	1	23	20	67	**70** in 6286

## Data Availability

Data are contained within the article and Supplementary Materials files.

## Supplementary Materials

The following supporting information can be downloaded at https://www.mdpi.com/article/10.3390/antiox14060653/s1, File S1: Materials and Methods: (1) Instruments and chemicals. (2) Extraction. (3) Evaluation of cell viability, lipid droplet accumulation inhibition assay, and TG assay. (4) Lipidomic analysis of neutral lipid: analysis of accumulation of triacylglycerols and oxidized hydroperoxide species using LC-MS/MS. (5) Metabolite profiles of AL1, AL3, and AL6 determined using NMR and LC-MS/MS analyses. (6) Rapid dereplication of AL1 and AL6 using 1D-NMR. (7) Lipid droplet accumulation inhibition assay. (8) LC-MS instrument conditions. (9) Metabolite profiling of AL extracts using DFF and 1H-NMR analyses; Table S1: List of selected Allium extracts used in this study; Table S2: LC/MS data of detected triacylglycerol species (treated with OA); Table S3: LC/MS data of detected triacylglycerol species (treated with LA); Table S4: Detected hydroperoxide of triacylglycerol species (treated with OA); Table S5: Detected hydroperoxides of triacylglycerol species (treated with LA); Figure S1. List and pictures of parts of Allium AL1–9 used in the study; Figure S2: Molecular net-working of AL1–AL9; Figure S3: LC-MS profiling of bioactive AL1 extract. (A) Diagnostic Frag-mentation Filtering (DFF) plot for metabolite analysis. (B) m/z list MS (n = 3) of AL1; (C) 3D visualization of MS data of AL1; Figure S4: LC-MS profiling of bioactive AL3 extract. (A) Diagnostic Fragmentation Filtering (DFF) plot for metabolite analysis; (B) m/z list MS (n = 3) of AL3; (C) 3D visualization of MS data of AL3; Figure S5: LC-MS profiling of bioactive AL6 extract. (A) Diagnostic Fragmentation Filtering (DFF) plot for metabolite analysis. (B) m/z list MS (n = 3) of AL6; (C) 3D visualization of MS data of AL6; Figure S6: Dereplication analysis using MixONat, structure of top 50 metabolites: compounds S**1**–S**10** from DB1; Figure S7: Dereplication analysis using MixONat, structure of top 50 metabolites: compounds S**11**–S**20** from DB1; Figure S8: Dereplication analysis using MixONat, structure of top 50 metabolites: compounds S**21**–S**30** from DB1; Figure S9: Dereplication analysis using MixONat, structure of top 50 metabolites: compounds S**31**–S**40** from DB1; Figure S10: Dereplication analysis using MixONat, structure of top 50 metabolites: compounds S**41**–S**50** from DB1; Figure S11: Dereplication analysis using MixONat, structure of top 50 metabolites: compounds S**51**–S**60** from DB2; Figure S12: Dereplication analysis using MixONat, structure of top 50 metabolites: compounds S**61**–S**70** from DB2; Figure S13: Dereplication analysis using MixONat, structure of top 50 metabolites: compounds S**71**–S**80** from DB2; Figure S14: Dereplication analysis using MixONat, structure of top 50 metabolites: compounds S**81**–S**90** from DB2; Figure S15: Dereplication analysis using MixONat, structure of top 50 metabolites: compounds S**91**–S**100** from DB2; Figure S16: Dereplication analysis using MixONat, structure of top 50 metabolites: compounds S**101**–S**110** from DB3; Figure S17: Dereplication analysis using MixONat, structure of top 50 metabolites: compounds S**111**–S**120** from DB3; Figure S18: Dereplication analysis using MixONat, structure of top 50 metabolites: compounds S**121**–S**130** from DB3; Figure S19: Dereplication analysis using MixONat, structure of top 50 metabolites: compounds S**131**–S**140** from DB3; Figure S20: Dereplication analysis using MixONat, structure of top 50 metabolites: compounds S**141**–S**150** from DB3; Figure S21: Dereplication analysis using MixONat, structure of top 50 metabolites: compounds S**151**–S**160** from DB3; Figure S22: Dereplication analysis using MixONat, structure of top 50 metabolites: compounds S**161**–S**170** from DB3; Figure S23: HPLC spectra of AL1 and AL6 (left, AL1; right, AL6); Figure S24: NMR profile of AL1 and AL6; Figure S25: Mixture analysis LC-MS/MS experimental flow; File S2: Supporting information for comparison of inhibited TG(OOH)n lipid species in OA and LA by AL6 and their proposed molecular lipid species with C18: 1 and C18:2; File S3: Supporting information for score 1 dereplicated compounds (Rank, Score, ID, LTS, MW, SMILES, NP Pathway, NP Superclass, NP Class, and Matched Spectrum Shifts, SDF Shifts).

## Abbreviations

LDA, lipid droplet accumulation; LDAI, lipid droplet accumulation inhibition; MAFLD, metabolic dysfunction-associated fatty liver disease; MASH, metabolic dysfunction-associated steatohepatitis; oxLD, oxidized lipid droplet; OA, oleic acid; LA, linoleic acid; EB, ethidium bromide; AO, acridine orange; LD, lipid droplet; TAGs, triacylglycerols; TGOOH, triacylglycerol hydroperoxide.; FFA, free fatty acid; LC/MS, liquid chromatography/mass spectrometry; NMR, nuclear magnetic resonance; HPLC, high-performance liquid chromatography; LTS, lotus.
