# Supplementary material for: Inhibition of Triacylglycerol Accumulation and Oxidized Hydroperoxides in Hepatocytes by Allium cepa (Bulb)
Source: Antioxidants (Basel). 2025 May 29;14(6):653. doi: 10.3390/antiox14060653 (PMC12190010; doi:10.3390/antiox14060653)
Supplement: Supplementary file 1 [file antioxidants-14-00653-s001.zip › File S1-S3/File S1_Antioxidant-Supplementary Information-2024-[Dibwe DF-Allium AL1]-Prof Hui-Dibwe For Submission 20250227-v2-Final 20250305 v6 Final approval.pdf]

Supporting Information

Inhibition of triacylglycerol accumulation and oxidized hydroperoxides in hepatocytes by *Allium cepa* (bulb)

Dya Fita Dibwe [1], Saki Oba [2], Satomi Monde [2], and Shu-Ping Hui [1,*]

[1] Faculty of Health Sciences, Hokkaido University, Kita-12, Nishi-5, Kita-Ku, Sapporo 060-0812, Japan;
[2] Graduate School of Health Sciences, Hokkaido University, Kita-12, Nishi-5, Kita-Ku, Sapporo 060-0812, Japan;

* Correspondences: keino@hs.hokudai.ac.jp; Tel./Fax: +81-11-706-36

Supporting Information

Table of contents:

Materials and Methods P5

1. Instruments and Chemicals P5
2. Extraction P6
3. Evaluation of cell viability, Lipid droplet accumulation inhibition assay and TG assay P6
4. Lipidomic analysis of neutral lipid: Analysis of accumulation on triacylglycerols oxidized hydroperoxides species by LC-MS/MS P7
5. Metabolite profile by NMR and LC-MS/MS analysis of AL1, AL3 and AL6 P9
6. Rapid dereplication using 1D-NMR of AL1 and AL6. P10
7. Lipid droplet accumulation inhibition assay P11
8. LC-MS instrument conditions P11
9. Metabolite profiling via DFF and, ^{1}H-NMR analyses of AL extracts P12

Table S1. List of selected *Allium* used in this study. P13

Figure S1. List and pictures of part of Allium AL1-9 used in the study. P14

Table S2. LC/MS data of detected triacylglycerol species (treated with OA). P15

Table S3. LC/MS data of detected triacylglycerol species (treated with LA). P16

Table S4. Detected hydroperoxide of triacylglycerol species (treated with OA). P17

Table S5. Detected hydroperoxide of triacylglycerol species (treated with LA). P19

Figure S2. Molecular networking of AL1−AL9 P21

Figure S3. LC-MS profiling of bioactive AL1 extract. (A) Diagnostic Fragmentation Filtering (DFF) plot for metabolites analysis. (B) *m/z* list MS(n=3) of AL-1 (B) 3D visualization of MS data of AL1. P22

Figure S4. LC-MS profiling of bioactive AL3 extract. (A) Diagnostic Fragmentation Filtering (DFF) plot for metabolites analysis. (B) *m/z* list MS(n=3) of AL3 (B) 3D visualization of MS data of AL3. P23

Figure S5. LC-MS profiling of bioactive AL6 extract. (A) Diagnostic Fragmentation Filtering (DFF) plot for metabolites analysis. (B) *m/z* list MS(n=3) of AL6 (B) 3D visualization of MS data of AL6. P25

Figure S6. Dereplication analysis from MixONat, structure of top 50 metabolites: compounds S**1**–**10** from DB1 P25

Figure S7. Dereplication analysis from MixONat, structure of top 50 metabolites: compounds S**11**–2**0** from DB1 P26

Figure S8. Dereplication analysis from MixONat, structure of top 50 metabolites: compounds S2**1**–3**0** from DB1 P27

Figure S9. Dereplication analysis from MixONat, structure of top 50 metabolites: compounds S3**1**–4**0** from DB1 P28

Figure S10. Dereplication analysis from MixONat, structure of top 50 metabolites: compounds S4**1**–5**0** from DB1 P29

Figure S11. Dereplication analysis from MixONat, structure of top 50 metabolites: compounds S5**1**–6**0** from DB2 P30

Figure S12. Dereplication analysis from MixONat, structure of top 50 metabolites: compounds S6**1**–7**0** from DB2 P31

Figure S13. Dereplication analysis from MixONat, structure of top 50 metabolites: compounds S7**1**–8**0** from DB2 P32

Figure S14. Dereplication analysis from MixONat, structure of top 50 metabolites: compounds S8**1**–9**0** from DB2 P33

Figure S15. Dereplication analysis from MixONat, structure of top 50 metabolites: compounds S9**1**–10**0** from DB2 P34

Figure S16. Dereplication analysis from MixONat, structure of top 50 metabolites: compounds S10**1**–110 from DB3 P35

Figure S17. Dereplication analysis from MixONat, structure of top 50 metabolites: compounds S11**1**–120 from DB3 P36

Figure S18. Dereplication analysis from MixONat, structure of top 50 metabolites: compounds S12**1**–13**0** from DB3 P37

Figure S19. Dereplication analysis from MixONat, structure of top 50 metabolites: compounds S13**1**–14**0** from DB3 P38

Figure S20. Dereplication analysis from MixONat, structure of top 50 metabolites: compounds S14**1**–15**0** from DB3 P39

Figure S21. Dereplication analysis from MixONat, structure of top 50 metabolites: compounds S15**1**–16**0** from DB3 P40

Figure S22. Dereplication analysis from MixONat, structure of top 50 metabolites: compounds S16**1**–17**0** from DB3 P41

Figure S23. HPLC spectra of AL1 and AL6 (Left: AL1, Right: AL6). P42

Figure S24. NMR profile of AL1 and AL6. P43

Figure S25. Mixture analysis LC-MS/MS experimental flow. P44

A. **Results of MixONat from DB1** P45

B. **Results of MixONat from DB2** P74

C. **Results of MixONat from DB3** P101

Materials and Methods

1. Instrument and chemicals

General Experimental Procedures: Nuclear magnetic resonance (NMR) analysis was conducted using a JEOL ECX400 Delta instrument, with tetramethylsilane (TMS) serving as the internal reference and chemical shifts denoted by δ values. High-resolution electrospray ionization mass spectrometry (HR-ESI-MS) was performed using an LTQ Orbitrap XL platform (Thermo Fisher Scientific Inc., San Jose, CA, USA). Methanol was sourced from Wako Chemicals. Cell culture supplies, including high-glucose Dulbecco's modified Eagle's medium (DMEM), Dulbecco's Phosphate-Buffered Saline (DPBS), trypsin EDTA, fetal bovine serum (FBS), and penicillin-streptomycin (100 U/mL), were procured from Gibco (Life Technologies, Carlsbad, CA, USA). Supplementary cell culture material was obtained from Corning (Corning, NY, USA). NMR data were acquired on a 400 MHz JNM-ECX400P system (JOEL, Japan). Data processing was performed using JOEL software, with chemical shifts (δ) reported in parts per million (ppm). Oleic acid (OA) was acquired from Cayman Chemical (Ann Arbor, MI, USA), and absorbance was measured using ARVO-MX (Perkin Elmer, Waltham, MA, USA) following established protocols. Oleic and linoleic acids were used in the present study. For triglyceride (TG) analysis, the following reagents were employed: RIPA Buffer (Nacalai Tesque, Kyoto, Japan), LabAssay Triglyceride (Waco Pure Chemical, Osaka, Japan), and Pierce BCA Assay Kit (Life Technologies, Carlsbad, CA, USA). Lipidomics analysis incorporated the EquiSPLASH LIPIDOMIX® quantitative mass spectrometry internal standard (Avanti Polar Lipids, Alabaster, AL, USA) as the reference. The liquid chromatography/mass spectrometry (LC/MS) mobile phase consisted of Ammonium Acetate (Wako Pure Chemical, Osaka, Japan) and LC-grade methanol (Kanto Chemical, Tokyo, Japan).

2. Extraction

The study utilized nine Allium specimens gathered by D.F.D and S.O from Sapporo's market in April 2020 and stored at the Health Innovation Center of the University’s Health Science Faculty (Table S1). The specimens were categorized as follows (Code, Scientific name, used part): AL1, Allium sativum (bulb), Ninniku; AL2, Allium victorialis (leaves), Gyoujaninnniku; AL3, Allium victorialis (stem), Gyoujaninnniku; AL4, Allium schoenoprasum var. foliosum (leaves), Asatuki, AL5, Allium schoenoprasum var. foliosum (stem), Asatuki; AL6, Allium cepa (bulb), Kitamanegi; AL7, Allium cepa (bulb), Sinntamanegi; AL8, Allium cepa (bulb), Sinntamanegi; and AL9, Allium cepa (bulb), Sinntamanegi. Nine distinct Allium food samples (AL1–AL9) were examined to identify the bioactive extracts and metabolites from food sources. The extraction procedure was as follows. Each food item was procured from a Sapporo market or grocery store in spring 2020 (Table S1). Initially, 10 g of each sample was crushed using a mortar and pestle and the resulting material was placed in a beaker. Subsequently, 100 mL methanol was added to the mixture for one hour. The mixture was sonicated twice for 30 min to obtain an extract. Methanol was eliminated by decompression and dried to produce the methanol extract. The biological activities of the extracts were assessed using the lipid droplet accumulation inhibition test.

3. Evaluation of cell viability, Lipid droplet accumulation inhibition assay and TG assay

Cytotoxicity and lipocytotoxicity evaluations were conducted using HepG2 cells obtained from the RIKEN BRC Cell Bank in Ibaraki, Japan, in accordance with the manufacturer's guidelines. LDAI activity was measured using an Oil Red O assay in 24-well plates with four replicate treatments following established methods. The LD staining procedure was implemented with adjustments based on previously published techniques. HepG2 cells were seeded in 12-well plates at 3.0×105 cells/well.

After 24 h of incubation, the cells were exposed to 0.25 mM oleic acid (OA) and test samples at concentrations of 150 μg/mL and 300 μg/mL for an additional 24 h. Next, triacylglycerol and protein levels were analyzed. Triacylglycerol and protein extraction were performed using modified versions of the Poudel and ATTO EzRIPA Lysis Kit protocols. The culture medium was removed and the cells were detached using 150 μL trypsin for 15 min. The cells were collected and centrifuged at 500×g for 5 min at 4 °C. The resulting cell pellet was washed twice with 400 μL of PBS, followed by centrifugation and removal of the supernatant. The washed pellet was resuspended in 500 μL RIPA buffer and maintained on ice for 15 min, with intermittent mixing every 1-2 minutes. The lysate was centrifuged at 14,000 × g and the supernatant was transferred to a new tube for storage at -80 °C. Triacylglycerol and protein levels were quantified according to the manufacturer's instructions using an xMark plate reader (Bio-Rad, Hercules, CA, USA). Triacylglycerol levels were normalized to the protein content. For the TG assay, total triacylglycerol concentration was determined by adjusting the concentration obtained from the TG kit to the total protein amount.

4. Lipidomic analysis of neutral lipid: Analysis of accumulation on triacylglycerols oxidized hydroperoxides species by LC-MS/MS

HepG2 cells were plated in 35 mm culture dishes at a density of 2.0×105 cells/dish and left to grow for 24 h. Subsequently, OA and test samples were introduced, with OA (0.25 mM and samples at concentrations of 150 μg/mL and 300 μg/mL. The next day, cellular lipids were extracted and examined using LC-MS. The extraction process began by removing the culture medium and rinsing the cells twice with 300 μL PBS. The cells were then treated with 300 μL of trypsin and incubated for 10 min at 37°C in a 5% CO2 environment. Following this, 300 μL of DMEM was added, and the cells were transferred to Eppendorf tubes. Two tubes, each containing 550 μL of cell suspension, were centrifuged at 13,000 rpm for 1 min at 4°C. After

discarding the supernatant, 400 μL of chloroform, 150 μL of methanol, and 10 μL of internal standard were added to each tube. The tubes were vortexed at 3,500 rpm for 10 min and centrifuged at 15,000 rpm at 4°C for 15 min. The resulting supernatant was collected in a separate tube, and the remaining cell mass was further treated with 400 μL chloroform and 150 μL methanol, followed by vortexing and centrifugation under the same conditions. The supernatant from the second extraction was then combined with the supernatant. After evaporating the solvent, 100 μL of methanol was added to the LC, vortexed at 3,500 rpm for 3 min, and centrifuged at 15,000 rpm at 4°C for 10 min. The final supernatant was analyzed by LC-MS in the positive mode. The analytical system consisted of a Shimadzu Prominence HPLC system (Shimadzu Corporation, Kyoto, Japan), LTQ Orbitrap mass spectrometer (Thermo Fisher Scientific Inc., San Jose, CA, USA), and electrospray ionization (ESI). Sample separation was performed on an Atlantis T3 C18 column (2.1 × 150 mm, 3 μm, Waters, Milford, MA, USA) at a flow rate of 200 μL/min and a column temperature of 40°C.

The mobile phase for liquid chromatography (LC) consisted of a mixture of 10 mM ammonium acetate solution, isopropanol, and methanol. High-resolution mass spectrometry (MS) data were collected using the Fourier transform mode. This research focused on triacylglycerols (TAG) and their oxidized variants as specific lipid molecules. Data were normalized based on cell counts. Following an established protocol [22,25,26], oleic acid-loaded HepG2 cells were exposed to crude extract (200 M) and incubated for 24 h at 37 °C. The samples were then centrifuged and analyzed using an LC-MS Orbitrap. Chromatographic separation was performed using a Shimadzu Prominence UHPLC system equipped with a binary solvent delivery system and standard autosampler. An Atlantis T3 column (2.1 × 150 mm, 3 μm, Waters) was used for separation at a flow rate of 200 μL/min. The mobile phase comprised a 10 mM ammonium acetate solution () in isopropanol (B) and methanol (C). For positive mode, the solvent ratios were: 0–1 min, 6% B and 90% C; 1–10 min, 83% B and 15% C; 10–19 min, 83% B and 15% C;

19–19.5 min, 6% B and 90% C; and 19.5–22 min, 6% B and 90% C. The injection volume was set at 10 μL and the column temperature was maintained at 40 °C. LC-MS parameters were consistent with those previously described [22,25,26].

5. Metabolite profile by NMR and LC-MS/MS analysis of AL1, AL3 and AL6

The mobile phase utilized was LC-grade methanol, obtained from Kanto Chemical in Tokyo, Japan. A CAPCELL PAK C18 UG120 Å 5 μm column, produced by Osaka Soda, Osaka, Japan, was employed. Methanol-d4 and DMSO-d6 for NMR analysis were procured from Sigma-Aldrich (St. Louis, MO, USA). For HPLC analysis, AL1, AL3, and AL6 extracts were prepared by dissolving in LC-grade methanol to achieve a 100 mg/mL concentration, then filtering through a 0.45 μm 13 mm syringe filter from Hawac Scientific (Xi'an, China). A Shimadzu Prominence HPLC system (Shimadzu Corporation, Kyoto, Japan) equipped with an Osaka Soda column (CAPCELL PAK C18 UG120 Å, 25×0.1 cm, 5 μm particle size) was used for sample analysis. The mobile phase consisted of Milli-Q water (A) and HPLC-grade methanol (B) with a flow rate of 5.0 mL/min. The solvent composition was programmed as: 0-1 min, 20% B; 1-20 min, 100% B; 20-23 min, 100% B; followed by a 2-minute post-run period. Chromatograms were recorded at 200 nm, 275 nm, and 343 nm wavelengths.

For NMR analysis, the extract was prepared at 10 mg/mL concentration in methanol-d4. An NMR spectrometer (ECX400, JEOL) was used to obtain 1H, 13C, and Dept (135 and 90) spectra. For LC-MS analysis, the extract was diluted to 100 μg/mL in methanol and filtered. Analysis was conducted using a Shimadzu Prominence HPLC system (Shimadzu Corporation, Kyoto, Japan) and an LTQ Orbitrap mass spectrometer (Thermo Fisher Scientific Inc., San Jose, CA, USA). Sample separation was performed on an Atlantis T3 C18 column (2.1 × 150 mm, 3 μm, Waters, Milford, MA) with a 200 μL/min flow rate. The column temperature was maintained at 40 °C, while the sample tray was kept at 4 °C. For measurements in positive mode,

the mobile phases comprised 10 mM ammonium acetate solution, isopropanol, and methanol. MS/MS data acquisition was carried out in ion trap mode, operating in a data-dependent manner.

6. Rapid dereplication using 1D-NMR of AL1 and AL6.

For 1D-NMR experiments (^{1}H-NMR, ^{13}C-NMR, DEPT-135, DEPT-90), a JOEL NMR spectrometer (ECX400, JEOL) was employed. Chemical shifts (δH and δC) are expressed in ppm. The methanol extract (30 mg) was dissolved in 600 µL CDCl3. ^{13}C-NMR spectra were recorded at 100 MHz. Data processing was conducted using JOEL software v6.3 (Delta NMR Processing and software), with calibration based on solvent peaks at δC 77.16 ppm (CDCl3). The procedure included manual phasing and baseline correction, followed by alignment of the DEPT experiments with the 13C spectra using a specific δC value.

MixONat, a free software developed by the SONAS lab at Université Angers (France), was designed for dereplication of natural product mixtures using ^{13}C NMR. This program compares the δC values of natural products in mixtures against a specified database considering multiplicities. It can be obtained from http://sourceforge.net/projects/mixonat. To use MixONat, users exported the peak list and intensity data from the experimental spectra (^{13}C-NMR, DEPT-90, and 135) as a reference.csv file using Microsoft Excel (Microsoft 16.45, Redmond, WA, USA). This file contains the δCs listed in descending order, with corresponding intensities on the same line separated by commas. The software processes these data using any dataset that provides molecular structures described SDF (c-type DB1-3 file format). MixONat generates compound proposals with scores ranging from 0 to 1 (0-100%), where 1 indicates a perfect match and 0 signifies no similarity for a given compound in the database. A score above 0.70 is considered acceptable for tentative identification. Following this, the experimental data of the natural products with the highest scores were compared with literature data.

7. Lipid droplet accumulation inhibition assay

To assess LDAI activity, Oil Red O assay was performed in 24-well plates (n = 4 for each treatment). This method involved staining LDs in cultured hepatocytes according to the manufacturer's instructions. HepG2 cells (1.5×104/well) were cultivated in media with 10% FBS, transferred to 35 mm dishes, and subjected to test samples after 24 h. Oil Red O, a fat-soluble dye, is typically used to visualize neutral lipids in LDs as previously described. The degree of LD inhibition for the studied ALs was determined by comparing them to the untreated control group (+OA) and normalizing the LDA absorbance values (%) in accordance with established methods. LD staining was performed as previously reported with minor adjustments.

8. LC-MS instrument conditions

An Atlantis T3 C18 column (2.1×150 mm, 3 μm, 155 Waters, Milford, MA, USA) was used for the separation of ALs samples, with a flow rate of 200 μL/min. The LC gradient elution employed a mobile phase comprising a 10 mM ammonium acetate solution, isopropanol, and methanol. Measurements were conducted in the positive mode, with the MS capillary voltage set to 4.04 kV. Both the sheath and auxiliary gases were nitrogen flowing at 50 and 20 psi, respectively. High-resolution MS data were collected within the m/z range of 150–1100. To obtain MS/MS spectra of low-resolution masses, data-dependent acquisition was employed using collision-induced dissociation (CID) in ion-trap mode. Xcalibur 2.2 (Thermo Fisher Scientific Inc., San Jose, CA, USA) was used to process the raw data as previously described.

9. Metabolite profiling via DFF and, ^{1}H-NMR analyses of AL extracts

Complex mixtures of structurally related compounds are often used to characterize natural products rather than isolated substances. Owing to their structural similarities, compounds within the same class frequently exhibit comparable MS/MS fragmentation patterns, resulting in shared product ions and/or neutral losses. To accurately identify all compounds of a specific class in the complex extracts, differential fragmentation filtering (DFF) was employed, and non-targeted LC-MS/MS datasets were examined for class-specific MS/MS spectra. A 3D LC-MS plot illustrates the LC-MS profile of the methanolic extract, displaying the retention time and MS values. LC-MS analysis of AL6 primarily revealed organosulfur compounds, carbohydrates and glucosidic metabolites, with small amounts of organic acids, and amino acids with diferrent scores

Comparison with in-house standards identified the main chemical components of the extract as major carbohydrates and iridoid glucosides, accompanied by minor organic acids, amino acids, and organosulfur compounds. DFF offers a rapid and efficient method for detecting entire compound classes in natural food product mixtures, which is particularly valuable for natural product dereplication and discovery. This technique was utilized to analyze metabolites from AL6's LC-MS/MS datasets, with the results presented as 3D images and their corresponding DFF. A graph displays the 3D LC-MS comparison of AL1, AL3, and AL6 using the DFF approach, showing the characteristic ion products and precursors from LC-MS/MS analysis.

GraphPad Prism (V7.0/10.1.2 was used for all statistical analyses. One-way ANOVA with Tukey's multiple comparison test was used for multiple comparisons. The significance level was set at 5 %. Results are expressed as the mean ± standard deviation (SD).

Table S1. List of selected *Allium* used in this study.

No	Species	abbr.	Family	Local name	Consumed/used as	The part used in this study
**1**	*Allium sativum*	AL1	*Allium*	Ninniku	cooked, raw	bulb
**2**	*Allium victorialis*	AL2	*Allium*	Gyoujaninnniku	cooked, raw	leaves
**3**	*Allium victorialis*	AL3	*Allium*	Gyoujaninnniku	cooked, raw	stem
**4**	*Allium schoenoprasum var. foliosum*	AL4	*Allium*	Asatuki	cooked, raw	leaves
**5**	*Allium schoenoprasum var. foliosum*	AL5	*Allium*	Asatuki	cooked, raw	stem
**6**	*Allium cepa*	AL6	*Allium*	Kitamanegi	cooked, raw	bulb
**7**	*Allium cepa*	AL7	*Allium*	Sinntamanegi	cooked, raw	bulb
**8**	*Allium cepa*	AL8	*Allium*	Sirotamanegi	cooked, raw	bulb
**9**	*Allium cepa*	AL9	*Allium*	Akatamanegi	cooked, raw	bulb

No	Species	Family	Local name	Consumed/used as	The part used in this study
1	*Allium sativum*	*Allium*	Ninniku	cooked, raw	bulb
2	*Allium victorialis*	*Allium*	Gyoujaninnniku	cooked, raw	leaves
3	*Allium victorialis*	*Allium*	Gyoujaninnniku	cooked, raw	stem
4	*Allium schoenoprasum var. foliosum*	*Allium*	Asatuki	cooked, raw	leaves
5	*Allium schoenoprasum var. foliosum*	*Allium*	Asatuki	cooked, raw	stem
6	*Allium cepa*	*Allium*	Kitamanegi	cooked, raw	bulb
7	*Allium cepa*	*Allium*	Sinntamanegi	cooked, raw	bulb
8	*Allium cepa*	*Allium*	Sirotamanegi	cooked, raw	bulb
9	*Allium cepa*	*Allium*	Akatamanegi	cooked, raw	bulb

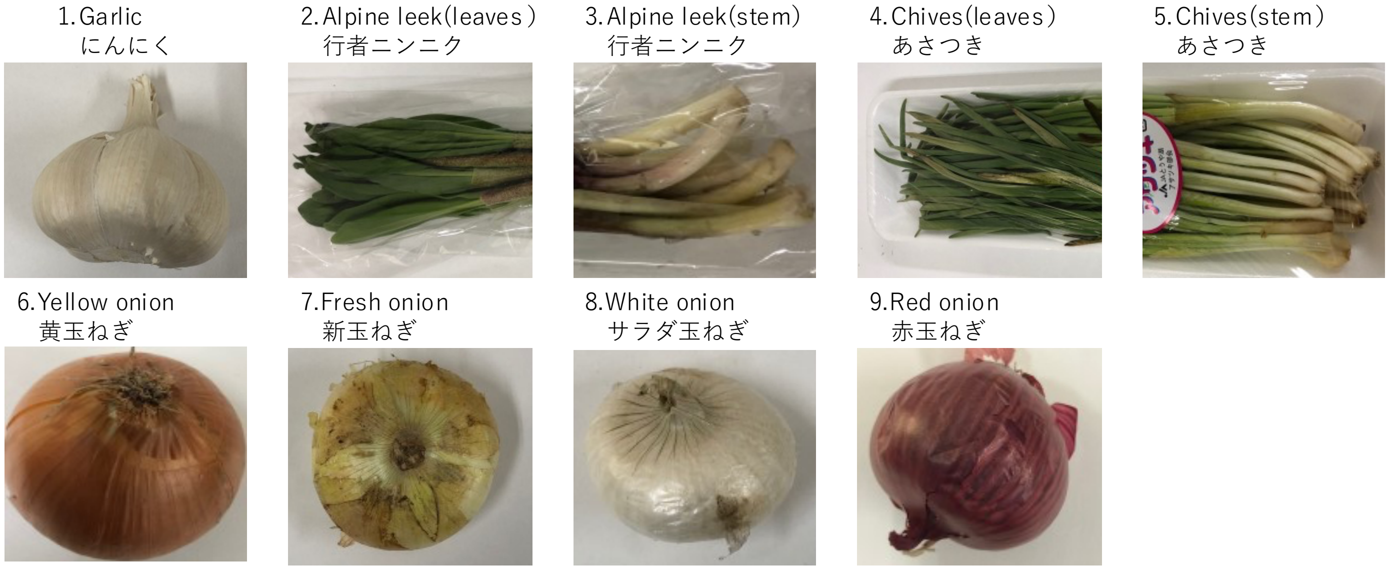

Figure S1. List and pictures of part of Allium AL1–9 used in the study

Table S2. LC/MS data of detected triacylglycerol species (treated with OA).

Lipid species	RT	Ion	calc. *m/z*	exptl. *m/z*	ppm	Lipid species	RT	Ion	calc. *m/z*	exptl. *m/z*	ppm
TG42:0	13.42	$[M+NH_4]^+$	740.6763	740.6774	1.49	TG54:0	15.82	$[M+NH_4]^+$	908.8641	908.8655	1.54
TG44:0	13.85	$[M+NH_4]^+$	768.7076	768.7087	1.43	TG54:1	15.39	$[M+NH_4]^+$	906.8484	906.8477	–0.77
TG46:0	14.20	$[M+NH_4]^+$	796.7389	796.7398	1.13	TG54:2	15.10	$[M+NH_4]^+$	904.8328	904.8336	0.88
TG46:1	13.90	$[M+NH_4]^+$	794.7232	794.7244	1.51	TG54:3	14.90	$[M+NH_4]^+$	902.8171	902.8178	0.78
TG46:2	13.60	$[M+NH_4]^+$	792.7076	792.7081	0.63	TG54:4	14.53	$[M+NH_4]^+$	900.8015	900.8016	0.11
TG46:3	13.31	$[M+NH_4]^+$	790.6919	790.6923	0.51	TG54:5	14.26	$[M+NH_4]^+$	898.7858	898.7858	0.00
TG48:0	14.58	$[M+NH_4]^+$	824.7702	824.7717	1.82	TG54:6	14.13	$[M+NH_4]^+$	896.7702	896.7698	–0.45
TG48:1	14.26	$[M+NH_4]^+$	822.7545	822.7558	1.58	TG54:7	13.94	$[M+NH_4]^+$	894.7545	894.7548	0.34
TG48:2	13.99	$[M+NH_4]^+$	820.7389	820.7396	0.85	TG54:8	13.63	$[M+NH_4]^+$	892.7389	892.7395	0.67
TG48:3	13.70	$[M+NH_4]^+$	818.7232	818.7234	0.24	TG56:4	14.93	$[M+NH_4]^+$	928.8328	928.8315	–1.40
TG48:5	11.27	$[M+NH_4]^+$	814.6169	814.6190	2.58	TG56:5	14.58	$[M+NH_4]^+$	926.8171	926.8160	–1.19
TG50:0	14.99	$[M+NH_4]^+$	852.8015	852.8041	3.05	TG56:6	14.47	$[M+NH_4]^+$	924.8015	924.8008	–0.76
TG50:1	14.60	$[M+NH_4]^+$	850.7858	850.7871	1.53	TG56:7	14.24	$[M+NH_4]^+$	922.7858	922.7859	0.11
TG50:2	14.33	$[M+NH_4]^+$	848.7702	848.7712	1.18	TG56:8	14.03	$[M+NH_4]^+$	920.7702	920.7707	0.54
TG50:3	14.10	$[M+NH_4]^+$	846.7545	846.7550	0.59	TG58:10	13.89	$[M+NH_4]^+$	944.7702	944.7710	0.85
TG50:4	13.83	$[M+NH_4]^+$	844.7389	844.7390	0.12	TG58:11	13.60	$[M+NH_4]^+$	942.7545	942.7537	–0.85
TG50:5	13.65	$[M+NH_4]^+$	842.7232	842.7231	–0.12	TG58:6	14.90	$[M+NH_4]^+$	952.8328	952.8326	–0.21
TG50:6	13.45	$[M+NH_4]^+$	840.7076	840.7075	–0.12	TG58:7	14.51	$[M+NH_4]^+$	950.8171	950.8157	–1.47
TG52:0	15.39	$[M+NH_4]^+$	880.8328	880.8357	3.29	TG58:8	14.36	$[M+NH_4]^+$	948.8015	948.8016	0.11
TG52:1	15.02	$[M+NH_4]^+$	878.8171	878.8176	0.57	TG58:9	14.13	$[M+NH_4]^+$	946.7858	946.7860	0.21
TG52:2	14.63	$[M+NH_4]^+$	876.8015	876.8026	1.25	TG60:10	14.20	$[M+NH_4]^+$	972.8015	972.8021	0.62
TG52:3	14.44	$[M+NH_4]^+$	874.7858	874.7862	0.46	TG60:11	14.06	$[M+NH_4]^+$	970.7858	970.7865	0.72
TG52:4	14.17	$[M+NH_4]^+$	872.7702	872.7702	0.00	TG60:12	13.83	$[M+NH_4]^+$	968.7702	968.7744	4.34
TG52:5	14.03	$[M+NH_4]^+$	870.7545	870.7544	–0.11	TG62:12	14.12	$[M+NH_4]^+$	996.8015	996.8027	1.20
TG52:6	13.81	$[M+NH_4]^+$	868.7389	868.7390	0.12	TG62:13	13.94	$[M+NH_4]^+$	994.7858	994.7869	1.11
TG52:7	13.54	$[M+NH_4]^+$	866.7232	866.7240	0.92						

Table S3. LC/MS data of detected triacylglycerol species (treated with LA).

Lipid species	RT	Ion	calc. *m/z*	exptl. *m/z*	ppm	Lipid species	RT	Ion	calc. *m/z*	exptl. *m/z*	ppm
TG42:0	13.46	$[M+NH_4]^+$	740.6763	740.6771	1.08	TG54:1	15.46	$[M+NH_4]^+$	906.8484	906.8491	0.77
TG44:0	13.84	$[M+NH_4]^+$	768.7076	768.7084	1.04	TG54:2	15.17	$[M+NH_4]^+$	904.8328	904.8333	0.55
TG46:0	14.34	$[M+NH_4]^+$	796.7389	796.7397	1.00	TG54:3	14.90	$[M+NH_4]^+$	902.8171	902.8173	0.22
TG46:1	13.91	$[M+NH_4]^+$	794.7232	794.7240	1.01	TG54:4	14.63	$[M+NH_4]^+$	900.8015	900.8019	0.44
TG46:2	13.64	$[M+NH_4]^+$	792.7076	792.7084	1.01	TG54:5	14.34	$[M+NH_4]^+$	898.7858	898.7865	0.78
TG46:3	13.33	$[M+NH_4]^+$	790.6919	790.6922	0.38	TG54:6	14.02	$[M+NH_4]^+$	896.7702	896.7710	0.89
TG48:0	14.70	$[M+NH_4]^+$	824.7702	824.7715	1.58	TG54:7	13.75	$[M+NH_4]^+$	894.7545	894.7553	0.89
TG48:1	14.40	$[M+NH_4]^+$	822.7545	822.7558	1.58	TG54:8	13.53	$[M+NH_4]^+$	892.7389	892.7394	0.56
TG48:2	14.09	$[M+NH_4]^+$	820.7389	820.7398	1.10	TG56:10	13.46	$[M+NH_4]^+$	916.7389	916.7396	0.76
TG48:3	13.72	$[M+NH_4]^+$	818.7232	818.7242	1.22	TG56:4	14.99	$[M+NH_4]^+$	928.8328	928.8325	◎0.32
TG48:4	13.44	$[M+NH_4]^+$	816.7076	816.7078	0.24	TG56:5	14.66	$[M+NH_4]^+$	926.8171	926.8163	◎0.86
TG50:0	15.06	$[M+NH_4]^+$	852.8015	852.8031	1.88	TG56:6	14.40	$[M+NH_4]^+$	924.8015	924.8008	◎0.76
TG50:1	14.74	$[M+NH_4]^+$	850.7858	850.7874	1.88	TG56:7	14.16	$[M+NH_4]^+$	922.7858	922.7846	◎1.30
TG50:2	14.45	$[M+NH_4]^+$	848.7702	848.7719	2.00	TG56:8	13.89	$[M+NH_4]^+$	920.7702	920.7698	◎0.43
TG50:3	14.16	$[M+NH_4]^+$	846.7545	846.7562	2.01	TG56:9	13.68	$[M+NH_4]^+$	918.7545	918.7543	◎0.22
TG50:4	13.82	$[M+NH_4]^+$	844.7389	844.7404	1.78	TG58:10	13.84	$[M+NH_4]^+$	944.7702	944.7706	0.42
TG50:5	13.57	$[M+NH_4]^+$	842.7232	842.7241	1.07	TG58:11	13.63	$[M+NH_4]^+$	942.7545	942.7546	0.11
TG50:6	13.31	$[M+NH_4]^+$	840.7076	840.7082	0.71	TG58:6	14.81	$[M+NH_4]^+$	952.8328	952.8318	◎1.05
TG52:0	15.46	$[M+NH_4]^+$	880.8328	880.8342	1.59	TG58:7	14.56	$[M+NH_4]^+$	950.8171	950.8163	◎0.84
TG52:1	15.12	$[M+NH_4]^+$	878.8171	878.8181	1.14	TG58:8	14.40	$[M+NH_4]^+$	948.8015	948.8013	◎0.21
TG52:2	14.81	$[M+NH_4]^+$	876.8015	876.8026	1.25	TG58:9	14.16	$[M+NH_4]^+$	946.7858	946.7856	◎0.21
TG52:3	14.56	$[M+NH_4]^+$	874.7858	874.7872	1.60	TG60:10	14.16	$[M+NH_4]^+$	972.8015	972.8019	0.41
TG52:4	14.23	$[M+NH_4]^+$	872.7702	872.7722	2.29	TG60:11	13.91	$[M+NH_4]^+$	970.7858	970.7863	0.52
TG52:5	13.86	$[M+NH_4]^+$	870.7545	870.7554	1.03	TG60:12	13.75	$[M+NH_4]^+$	968.7702	968.7708	0.62
TG52:6	13.63	$[M+NH_4]^+$	868.7389	868.7396	0.81	TG60:13	13.53	$[M+NH_4]^+$	966.7545	966.7554	0.93
TG52:7	13.46	$[M+NH_4]^+$	866.7232	866.7241	1.04	TG62:12	14.20	$[M+NH_4]^+$	996.8015	996.8010	◎0.50
TG52:8	13.08	$[M+NH_4]^+$	864.7076	864.7084	0.93	TG62:13	13.84	$[M+NH_4]^+$	994.7858	994.7875	1.71
TG54:0	15.83	$[M+NH_4]^+$	908.8641	908.8653	1.32	TG62:14	13.70	$[M+NH_4]^+$	992.7702	992.7709	0.71

Table S4. Detected hydroperoxide of triacylglycerol species (treated with OA).

Lipid species	RT	Ion	calc. *m/z*	exptl. *m/z*	ppm
TGOOH52:7	1.98	$[M+NH_4]^+$	898.7130	898.7126	◎0.45
TGOOH54:7	2.05	$[M+NH_4]^+$	926.7443	926.7438	◎0.54
TGOOH56:10	10.21	$[M+NH_4]^+$	948.7287	948.7275	◎1.26
TGOOH58:10	10.86	$[M+NH_4]^+$	976.7600	976.7565	◎3.58
TGOOH58:11	10.23	$[M+NH_4]^+$	974.7443	974.7411	◎3.28
TGOOH60:14	10.82	$[M+NH_4]^+$	996.7287	996.7333	4.62
TGOOH60:15	10.21	$[M+NH_4]^+$	994.7130	994.7175	4.52
TGOOH66:18	13.18	$[M+NH_4]^+$	1072.7600	1072.7618	1.68

Lipid species	RT	Ion	calc. *m/z*	exptl. *m/z*	ppm
$TG(-OOH)_2$ 46:2	14.15	$[M+NH_4]^+$	856.6872	856.6878	0.70
$TG(-OOH)_2$ 46:3	13.81	$[M+NH_4]^+$	854.6716	854.6718	0.23
$TG(-OOH)_2$ 48:2	14.54	$[M+NH_4]^+$	884.7185	884.7195	1.13
$TG(-OOH)_2$ 48:3	14.17	$[M+NH_4]^+$	882.7029	882.7031	0.23
$TG(-OOH)_2$ 48:4	14.03	$[M+NH_4]^+$	880.6872	880.6877	0.57
$TG(-OOH)_2$ 48:5	13.78	$[M+NH_4]^+$	878.6716	878.6724	0.91
$TG(-OOH)_2$ 50:2	14.97	$[M+NH_4]^+$	912.7498	912.7491	◎0.77
$TG(-OOH)_2$ 50:5	14.15	$[M+NH_4]^+$	906.7029	906.7034	0.55
$TG(-OOH)_2$ 50:6	13.87	$[M+NH_4]^+$	904.6872	904.6877	0.55
$TG(-OOH)_2$ 52:2	15.29	$[M+NH_4]^+$	940.7811	940.7808	◎0.32
$TG(-OOH)_2$ 52:3	14.97	$[M+NH_4]^+$	938.7655	938.7665	1.07
$TG(-OOH)_2$ 52:6	14.22	$[M+NH_4]^+$	932.7185	932.7189	0.43
$TG(-OOH)_2$ 52:7	14.12	$[M+NH_4]^+$	930.7029	930.7034	0.54
$TG(-OOH)_2$ 52:8	13.81	$[M+NH_4]^+$	928.6872	928.6873	0.11
$TG(-OOH)_2$ 54:2	15.66	$[M+NH_4]^+$	968.8124	968.8128	0.41
$TG(-OOH)_2$ 54:3	15.33	$[M+NH_4]^+$	966.7968	966.7961	◎0.72

TG(-OOH)$_2$ 54:8	14.15	$[M+NH_4]^+$	956.7185	956.7189	0.42

**Lipid species**	**RT**	**Ion**	**calc. *m/z***	**exptl. *m/z***	**ppm**
TG(-OOH)$_3$ 54:10	10.18	$[M+NH_4]^+$	984.6771	984.6758	◎1.32
TG(-OOH)$_3$ 54:3	13.70	$[M+NH_4]^+$	998.7866	998.7878	1.20
TG(-OOH)$_3$ 54:4	13.43	$[M+NH_4]^+$	996.7710	996.7726	1.61
TG(-OOH)$_3$ 56:10	10.81	$[M+NH_4]^+$	1012.7084	1012.7070	◎1.38
TG(-OOH)$_3$ 56:11	10.19	$[M+NH_4]^+$	1010.6927	1010.6912	◎1.48
TG(-OOH)$_3$ 58:11	10.88	$[M+NH_4]^+$	1038.7240	1038.7227	◎1.25
TG(-OOH)$_3$ 58:7	13.42	$[M+NH_4]^+$	1046.7866	1046.7837	◎2.77

Table S5. Detected hydroperoxide of triacylglycerol species (treated with LA).

Lipid species	RT	Ion	calc. *m/z*	exptl. *m/z*	ppm
TGOOH48:5	11.40	$[M+NH_4]^+$	846.6817	846.6847	3.54
TGOOH52:4	11.84	$[M+NH_4]^+$	904.7600	904.7596	◎0.44
TGOOH54:5	11.41	$[M+NH_4]^+$	930.7756	930.7767	1.18
TGOOH54:6	11.48	$[M+NH_4]^+$	928.7600	928.7604	0.43
TGOOH54:9	6.82	$[M+NH_4]^+$	922.7130	922.7124	◎0.65
TGOOH56:10	10.17	$[M+NH_4]^+$	948.7287	948.7265	◎2.32
TGOOH56:7	13.93	$[M+NH_4]^+$	954.7756	954.7752	◎0.42
TGOOH58:10	10.84	$[M+NH_4]^+$	976.7600	976.7582	◎1.84
TGOOH58:11	10.24	$[M+NH_4]^+$	974.7443	974.7421	◎2.26
TGOOH60:13	10.82	$[M+NH_4]^+$	998.7443	998.7407	◎3.60
TGOOH60:14	10.85	$[M+NH_4]^+$	996.7287	996.7334	4.72
TGOOH60:15	10.21	$[M+NH_4]^+$	994.7130	994.7176	4.62
TGOOH66:18	13.22	$[M+NH_4]^+$	1072.7600	1072.7616	1.49

Lipid species	RT	Ion	calc. *m/z*	exptl. *m/z*	ppm
$TG(-OOH)_2$ 48:5	13.67	$[M+NH_4]^+$	878.6716	878.6732	1.82
$TG(-OOH)_2$ 50:2	14.99	$[M+NH_4]^+$	912.7498	912.7510	1.31
$TG(-OOH)_2$ 50:6	13.80	$[M+NH_4]^+$	904.6872	904.6870	◎0.22
$TG(-OOH)_2$ 52:2	15.30	$[M+NH_4]^+$	940.7811	940.7806	◎0.53
$TG(-OOH)_2$ 52:3	15.06	$[M+NH_4]^+$	938.7655	938.7649	◎0.64
$TG(-OOH)_2$ 54:2	15.78	$[M+NH_4]^+$	968.8124	968.8132	0.83
$TG(-OOH)_2$ 54:3	15.35	$[M+NH_4]^+$	966.7968	966.7972	0.41
$TG(-OOH)_2$ 56:6	14.99	$[M+NH_4]^+$	988.7811	988.7799	◎1.21
$TG(-OOH)_2$ 58:7	15.02	$[M+NH_4]^+$	1014.7968	1014.7980	1.18

TG(-OOH)$_2$ 62:13	6.95	$[M+NH_4]^+$	1058.7655	1058.7658	0.28

**Lipid species**	**RT**	**Ion**	**calc. *m/z***	**exptl. *m/z***	**ppm**
TG(-OOH)$_3$ 50:3	12.59	$[M+NH_4]^+$	942.7240	942.7233	◎0.74
TG(-OOH)$_3$ 52:3	13.07	$[M+NH_4]^+$	970.7553	970.7550	◎0.31
TG(-OOH)$_3$ 52:4	12.69	$[M+NH_4]^+$	968.7397	968.7404	0.72
TG(-OOH)$_3$ 52:5	12.48	$[M+NH_4]^+$	966.7240	966.7243	0.31
TG(-OOH)$_3$ 54:10	10.22	$[M+NH_4]^+$	984.6771	984.6765	◎0.61
TG(-OOH)$_3$ 54:3	13.77	$[M+NH_4]^+$	998.7866	998.7881	1.50
TG(-OOH)$_3$ 54:4	13.52	$[M+NH_4]^+$	996.7710	996.7707	◎0.30
TG(-OOH)$_3$ 54:5	12.71	$[M+NH_4]^+$	994.7553	994.7553	0.00
TG(-OOH)$_3$ 54:6	12.41	$[M+NH_4]^+$	992.7397	992.7394	◎0.30
TG(-OOH)$_3$ 56:10	10.85	$[M+NH_4]^+$	1012.7084	1012.7072	◎1.18
TG(-OOH)$_3$ 56:11	10.22	$[M+NH_4]^+$	1010.6927	1010.6913	◎1.39
TG(-OOH)$_3$ 58:11	10.84	$[M+NH_4]^+$	1038.7240	1038.7235	◎0.48
TG(-OOH)$_3$ 58:7	13.44	$[M+NH_4]^+$	1046.7866	1046.7822	◎4.20
TG(-OOH)$_3$ 60:11	13.42	$[M+NH_4]^+$	1066.7553	1066.7515	◎3.56
TG(-OOH)$_3$ 66:18	12.25	$[M+NH_4]^+$	1136.7397	1136.7347	◎4.40

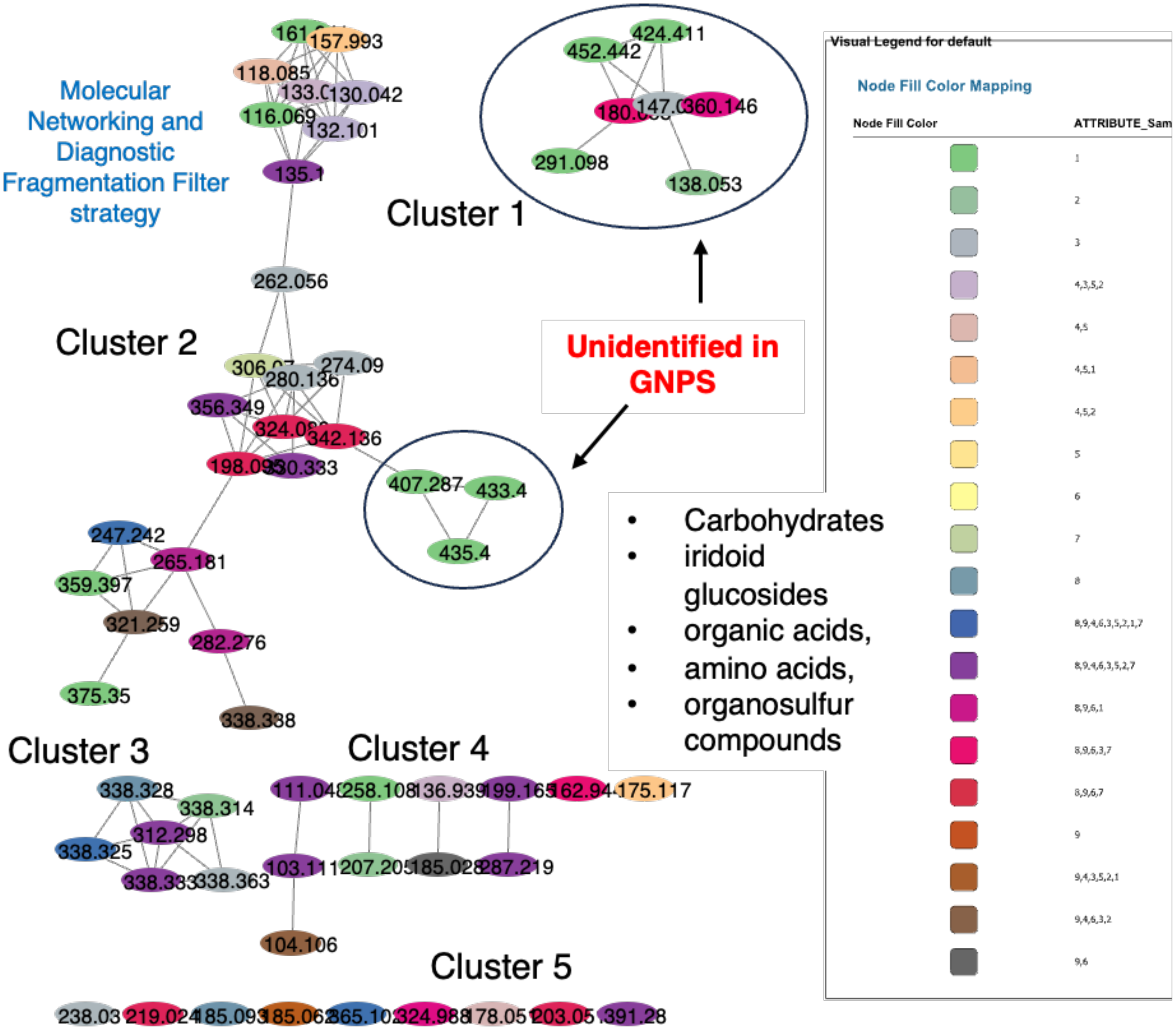

Figure S2. Molecular networking of AL1–AL9.

A

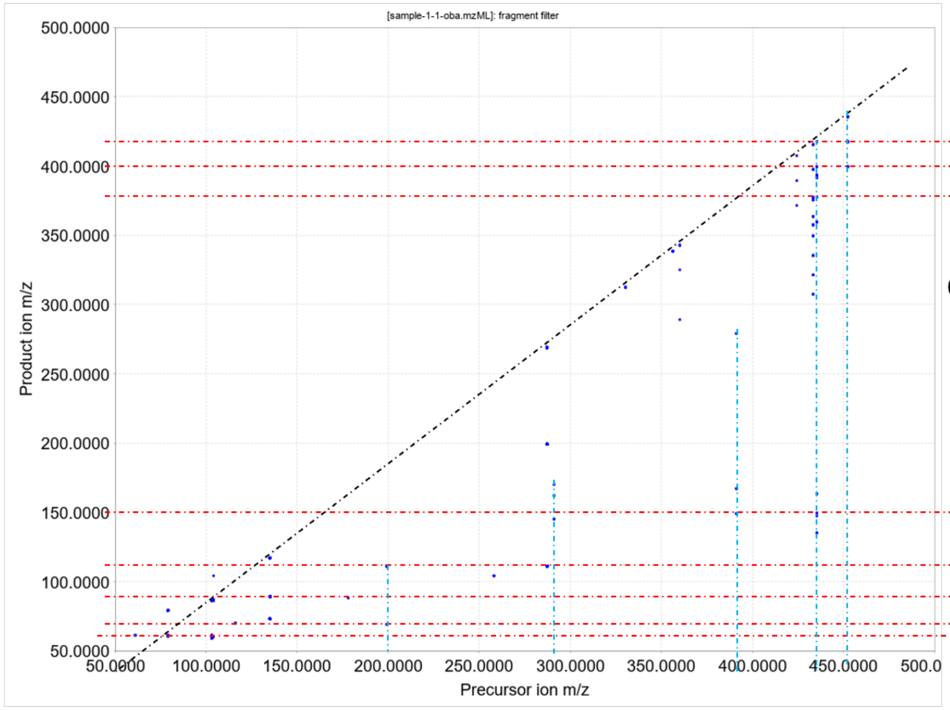

Represent the diagnostic product ions that are required to be considered a putative metabolites x

Represent the [M + H]+ precursor ions.

B

	Allium 1			
	RT (min)	MS1 (m/z)	MS2 (m/z)	MS3 (m/z)
1	0.01	435.4147	359.3890	148.9953<br>134.9374
2	0.01	424.4102	407.2867	371.3444
3	1.80	291.0977	161.9223	144.9228
4	1.88	360.1461	324.9067	289.0067
5	1.93	116.0693	69.9151	can't detect
6	1.96	258.1073	103.9791	59.9807
7	2.26	104.1058	60.0242	can't detect
8	10.41	330.3329	312.3188	102.0371
9	10.90	356.3483	338.3411	101.8994
10	12.51	391.2800	148.8773	121.0314
11	12.96	433.3988	375.3741	357.3410
12	13.12	452.4411	435.3180	399.4792

C

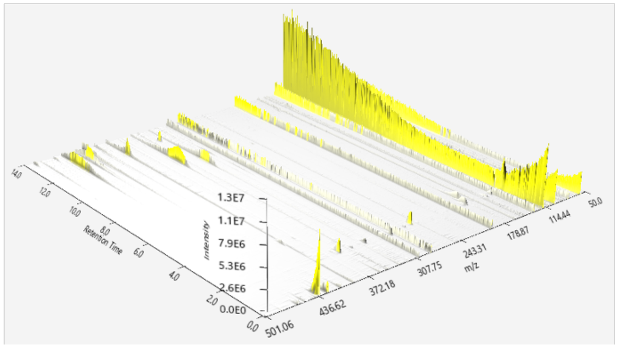

Figure S3. LC-MS profiling of bioactive AL1 extract. (A) Diagnostic Fragmentation Filtering (DFF) plot for metabolites analysis. (B) *m/z* list MS(n=3) of AL1 (B) 3D visualization of MS data of AL1.

A

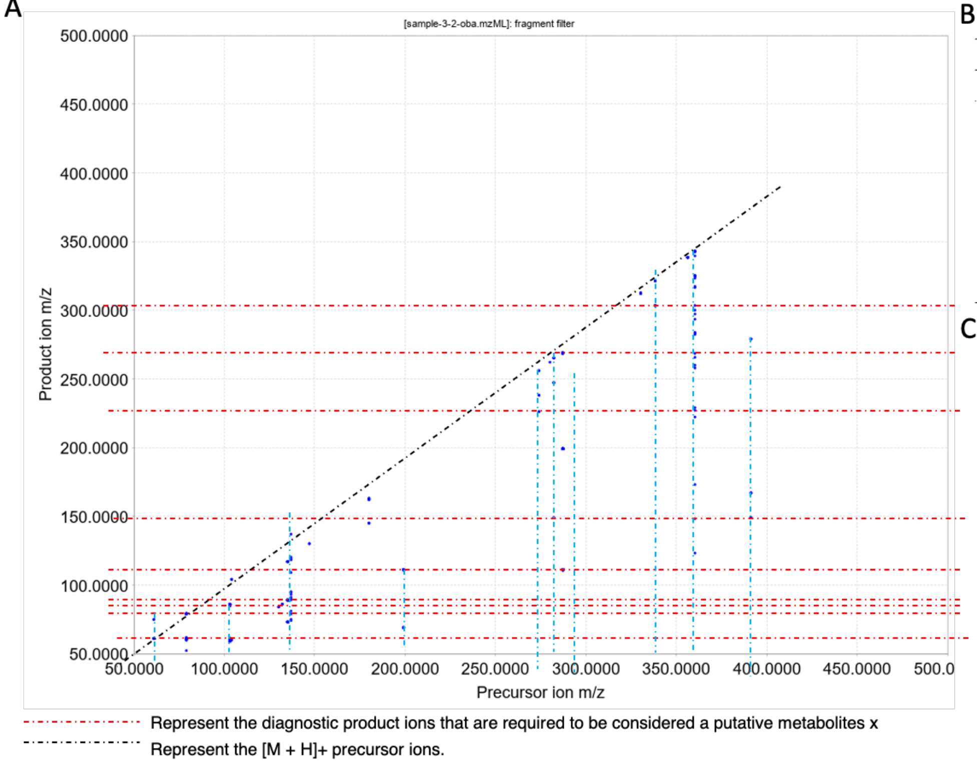

B

	Allium 3			
	RT (min)	MS1 (m/z)	MS2 (m/z)	MS3 (m/z)
1	2.25	104.1058	60.0135	can't detect
2	10.53	330.3329	312.2917	101.9833
3	10.93	282.2759	265.1280	247.2555
4	10.93	356.3483	338.2873	101.9973
5	12.53	391.2800	148.9577	120.9727
6	12.90	338.3378	148.9526	121.0148

C

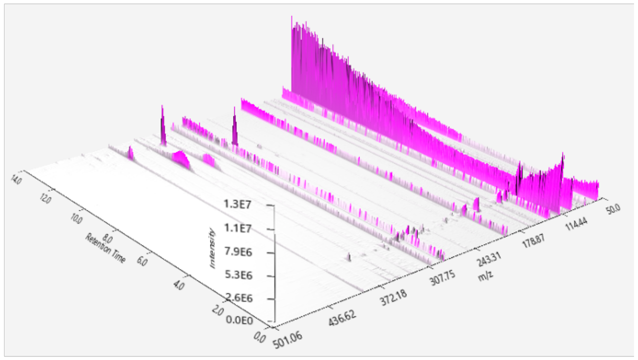

Figure S4. LC-MS profiling of bioactive AL3 extract. (A) Diagnostic Fragmentation Filtering (DFF) plot for metabolites analysis. (B) *m/z* list MS(n=3) of AL3 (B) 3D visualization of MS data of AL3.

A

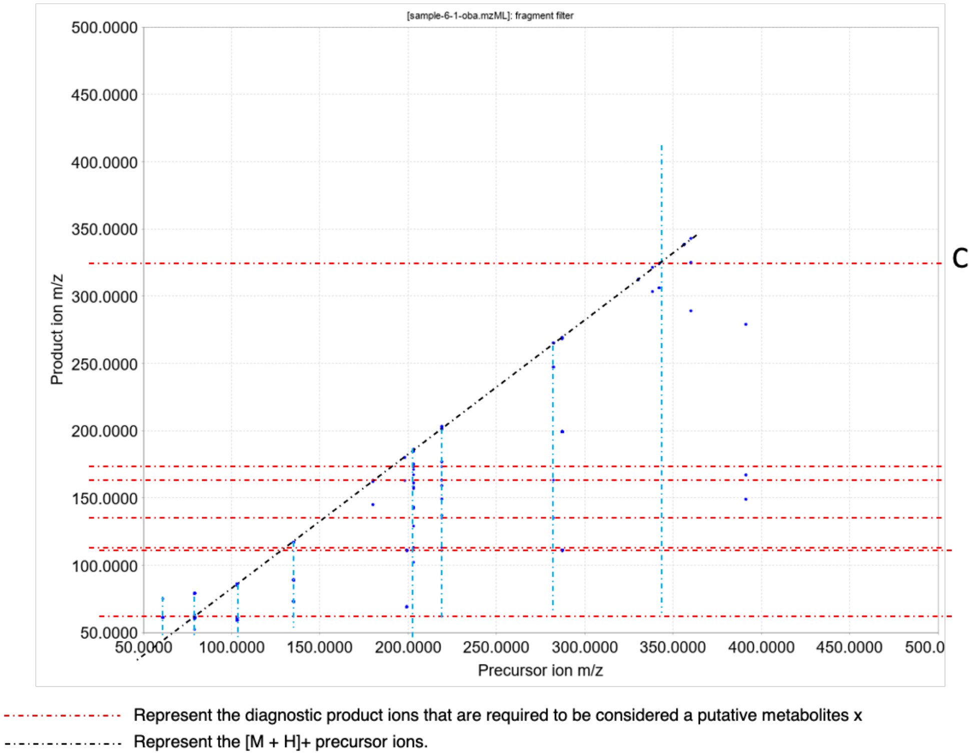

B

	Allium 6			
	RT (min)	MS1 (m/z)	MS2 (m/z)	MS3 (m/z)
1	1.87	360.1464	325.0564	288.9400
2	1.94	198.0951	179.9797	162.9164
3	1.94	180.0847	162.8774	145.0766
4	2.08	203.0504	185.0148	166.9430
5	2.14	219.0242	158.9557<br>176.7700	98.8628(158.9557)<br>159.1721(176.770<br>0)
6	2.27	104.1058	59.9128	can't detect
7	10.56	330.3330	312.2895	102.0410
8	10.79	356.3484	338.3188	101.9674
9	10.93	282.2759	265.2184<br>247.2157	247.2726(265.218<br>4)<br>163.1118(247.215<br>7)
10	12.48	391.2801	148.9516	121.0154

C

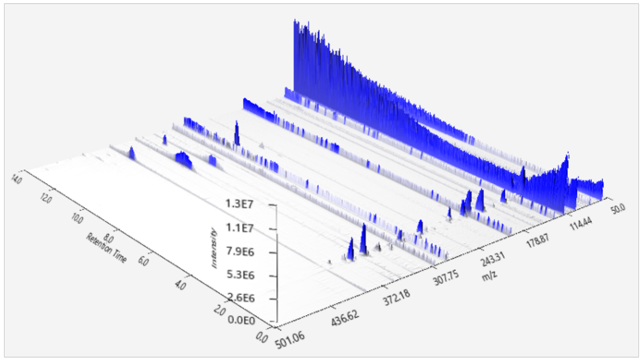

Figure S5. LC-MS profiling of bioactive AL6 extract. (A) Diagnostic Fragmentation Filtering (DFF) plot for metabolites analysis. (B) *m/z* list MS(n=3) of AL6 (B) 3D visualization of MS data of AL6.

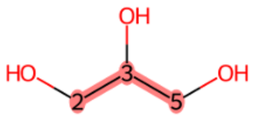

S1

Rank: 1 MW: 92.09
LTS0155285
Score: 1.0 (3/3 C)
Deviation : 1.04 ppm

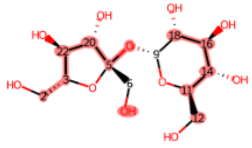

S2

Rank: 2 MW: 342.3
LTS0272557
Score: 1.0 (12/12 C)
Deviation : 2.12 ppm

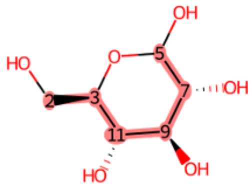

S3

Rank: 3 MW: 180.16
LTS0013597
Score: 1.0 (6/6 C)
Deviation : 2.2 ppm

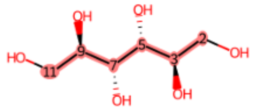

S4

Rank: 4 MW: 182.17
LTS0199986
Score: 1.0 (6/6 C)
Deviation : 2.78 ppm

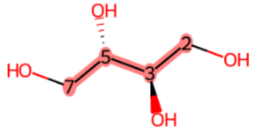

S5

Rank: 5 MW: 122.12
LTS0150163
Score: 1.0 (4/4 C)
Deviation : 2.82 ppm

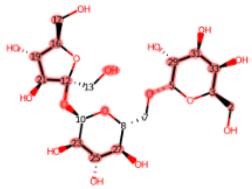

S6

Rank: 6 MW: 504.44
LTS0113066
Score: 1.0 (18/18 C)
Deviation : 4.29 ppm

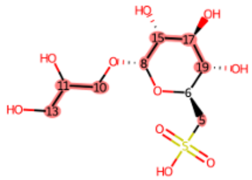

S7

Rank: 7 MW: 318.3
LTS0146582
Score: 0.89 (8/9 C)
Deviation : 3.66 ppm

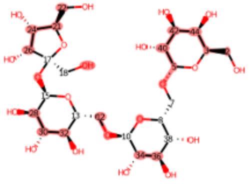

S8

Rank: 8 MW: 666.58
LTS0183884
Score: 0.88 (21/24 C)
Deviation : 4.74 ppm

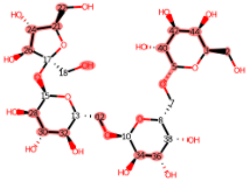

S9

Rank: 9 MW: 666.58
LTS0062089
Score: 0.88 (21/24 C)
Deviation : 4.74 ppm

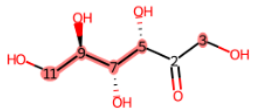

S10

Rank: 10 MW: 180.16
LTS0241114
Score: 0.83 (5/6 C)
Deviation : 0.8 ppm

Figure S6. Dereplication analysis from MixONat, structure of top 50 metabolites: compounds S**1**–S**10** from DB1

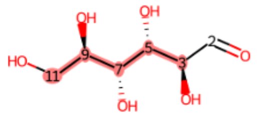

S11

Rank: 11 MW: 180.16
LTS0276202
Score: 0.83 (5/6 C)
Deviation : 0.88 ppm

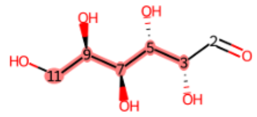

S12

Rank: 12 MW: 180.16
LTS0128031
Score: 0.83 (5/6 C)
Deviation : 1.18 ppm

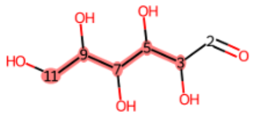

S13

Rank: 13 MW: 180.16
LTS0241274
Score: 0.83 (5/6 C)
Deviation : 1.18 ppm

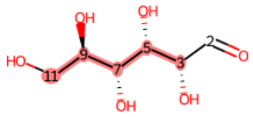

S14

Rank: 14 MW: 180.16
LTS0262158
Score: 0.83 (5/6 C)
Deviation : 1.39 ppm

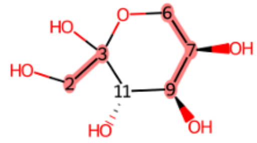

S15

Rank: 15 MW: 180.16
LTS0259277
Score: 0.83 (5/6 C)
Deviation : 1.58 ppm

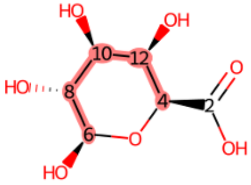

S16

Rank: 16 MW: 194.14
LTS0271461
Score: 0.83 (5/6 C)
Deviation : 2.62 ppm

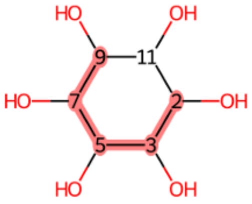

S17

Rank: 17 MW: 180.16
LTS0047771
Score: 0.83 (5/6 C)
Deviation : 3.87 ppm

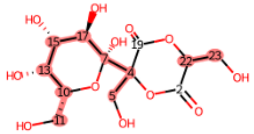

S18

Rank: 18 MW: 354.26
LTS0110247
Score: 0.83 (10/12 C)
Deviation : 4.1 ppm

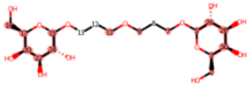

S19

Rank: 19 MW: 458.46
LTS0157972
Score: 0.83 (15/18 C)
Deviation : 7.91 ppm

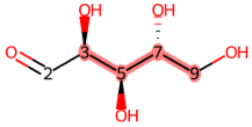

S20

Rank: 20 MW: 150.13
LTS0085513
Score: 0.8 (4/5 C)
Deviation : 0.27 ppm

Figure S7. Dereplication analysis from MixONat, structure of top 50 metabolites: compounds S**16**–S**20** from DB1

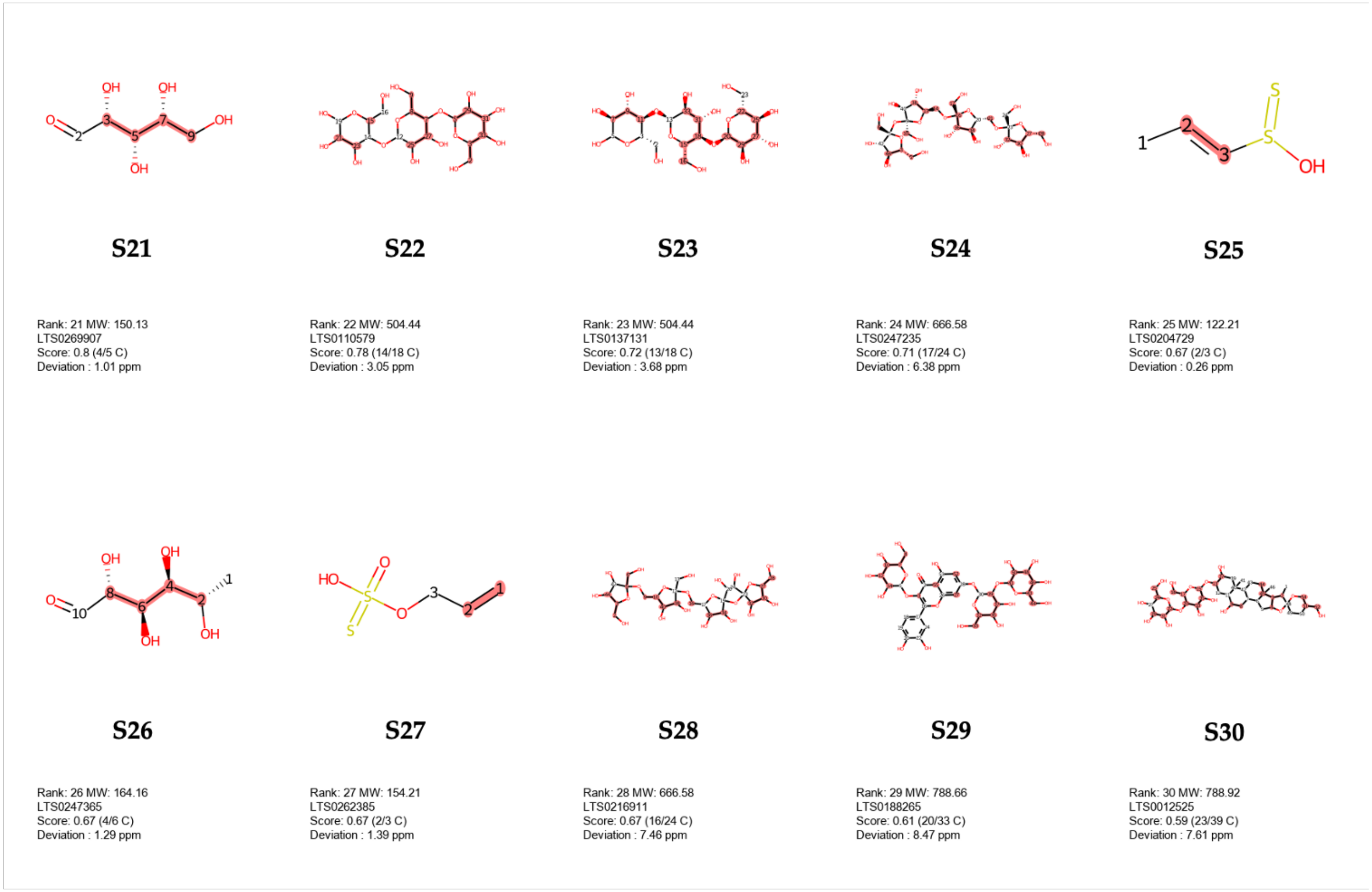

Figure S8. Dereplication analysis from MixONat, structure of top 50 metabolites: compounds S**21**–S**30** from DB1.

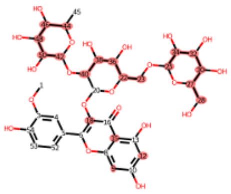

S31

Rank: 31 MW: 786.69
LTS0031032
Score: 0.59 (20/34 C)
Deviation : 7.69 ppm

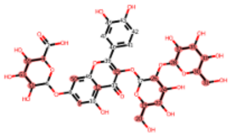

S32

Rank: 32 MW: 802.64
LTS0208698
Score: 0.58 (19/33 C)
Deviation : 5.65 ppm

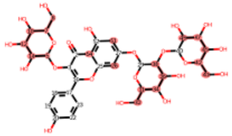

S33

Rank: 33 MW: 772.66
LTS0081415
Score: 0.58 (19/33 C)
Deviation : 6.61 ppm

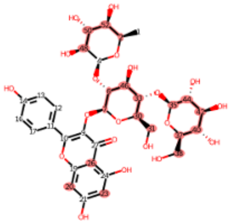

S34

Rank: 34 MW: 756.66
LTS0074468
Score: 0.58 (19/33 C)
Deviation : 8.89 ppm

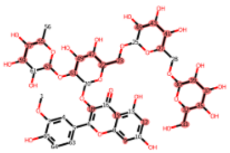

S35

Rank: 35 MW: 948.83
LTS0257423
Score: 0.57 (23/40 C)
Deviation : 8.49 ppm

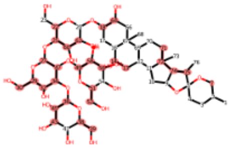

S36

Rank: 36 MW: 1097.2
LTS0129228
Score: 0.57 (29/51 C)
Deviation : 11.32 ppm

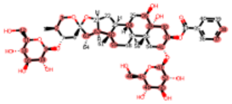

S37

Rank: 37 MW: 909.02
LTS0080305
Score: 0.57 (26/46 C)
Deviation : 12.52 ppm

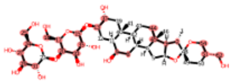

S38

Rank: 38 MW: 788.92
LTS0261262
Score: 0.56 (22/39 C)
Deviation : 6.78 ppm

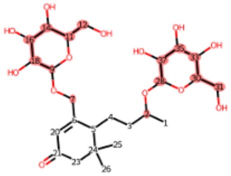

S39

Rank: 39 MW: 550.59
LTS0235000
Score: 0.56 (14/25 C)
Deviation : 5.46 ppm

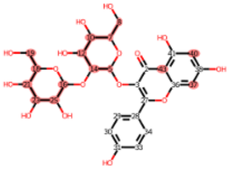

S40

Rank: 40 MW: 610.52
LTS0225418
Score: 0.56 (15/27 C)
Deviation : 5.66 ppm

Figure S9. Dereplication analysis from MixONat, structure of top 50 metabolites: compounds S**31**–S**40** from DB1.

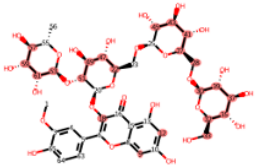

S41

Rank: 41 MW: 948.83
LTS0161746
Score: 0.55 (22/40 C)
Deviation : 7.73 ppm

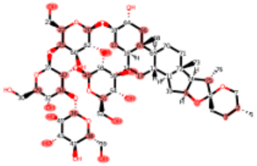

S42

Rank: 42 MW: 1097.2
LTS0210976
Score: 0.55 (28/51 C)
Deviation : 8.97 ppm

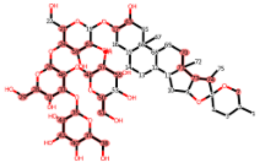

S43

Rank: 43 MW: 1081.2
LTS0138561
Score: 0.55 (28/51 C)
Deviation : 10.98 ppm

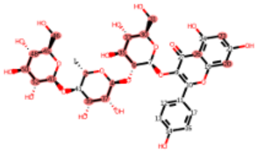

S44

Rank: 44 MW: 756.66
LTS0246349
Score: 0.55 (18/33 C)
Deviation : 5.63 ppm

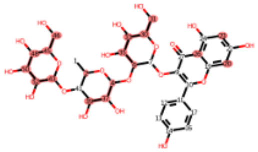

S45

Rank: 45 MW: 756.66
LTS0142113
Score: 0.55 (18/33 C)
Deviation : 5.67 ppm

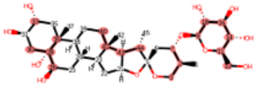

S46

Rank: 46 MW: 642.78
LTS0202010
Score: 0.55 (18/33 C)
Deviation : 6.6 ppm

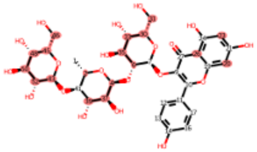

S47

Rank: 47 MW: 756.66
LTS0184521
Score: 0.55 (18/33 C)
Deviation : 6.86 ppm

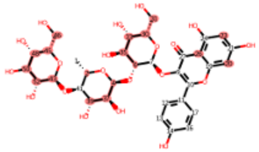

S48

Rank: 48 MW: 756.66
LTS0105990
Score: 0.55 (18/33 C)
Deviation : 7.8 ppm

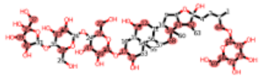

S49

Rank: 49 MW: 1101.19
LTS0126921
Score: 0.54 (27/50 C)
Deviation : 7.63 ppm

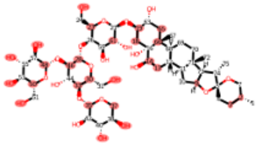

S50

Rank: 50 MW: 1083.17
LTS0008293
Score: 0.54 (27/50 C)
Deviation : 8.22 ppm

Figure S10. Dereplication analysis from MixONat, structure of top 50 metabolites: compounds S**41**–S**50** from DB1.

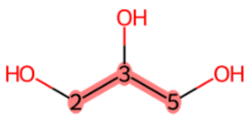

S51

Rank: 1 MW: 92.09
LTS0155285
Score: 1.0 (3/3 C)
Deviation : 1.04 ppm

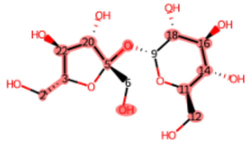

S52

Rank: 2 MW: 342.3
LTS0272557
Score: 1.0 (12/12 C)
Deviation : 2.12 ppm

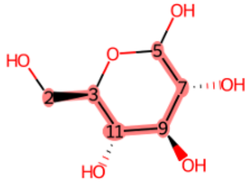

S53

Rank: 3 MW: 180.16
LTS0013597
Score: 1.0 (6/6 C)
Deviation : 2.2 ppm

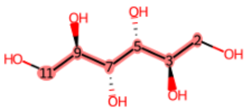

S54

Rank: 4 MW: 182.17
LTS0199986
Score: 1.0 (6/6 C)
Deviation : 2.78 ppm

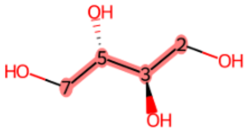

S55

Rank: 5 MW: 122.12
LTS0150163
Score: 1.0 (4/4 C)
Deviation : 2.82 ppm

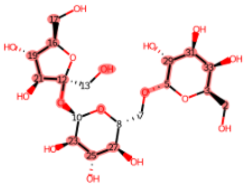

S56

Rank: 6 MW: 504.44
LTS0113066
Score: 1.0 (18/18 C)
Deviation : 4.29 ppm

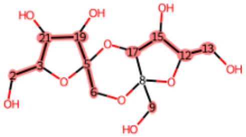

S57

Rank: 7 MW: 324.28
LTS0162289
Score: 0.92 (11/12 C)
Deviation : 4.53 ppm

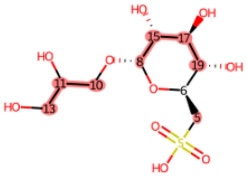

S58

Rank: 8 MW: 318.3
LTS0146582
Score: 0.89 (8/9 C)
Deviation : 3.66 ppm

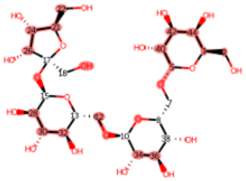

S59

Rank: 9 MW: 666.58
LTS0183884
Score: 0.88 (21/24 C)
Deviation : 4.74 ppm

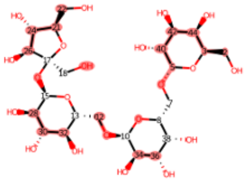

S60

Rank: 10 MW: 666.58
LTS0062089
Score: 0.88 (21/24 C)
Deviation : 4.74 ppm

Figure S11. Dereplication analysis from MixONat, structure of top 50 metabolites: compounds S**1**–S**10** from DB2

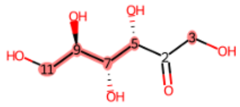

S61

Rank: 11 MW: 180.16
LTS0241114
Score: 0.83 (5/6 C)
Deviation : 0.8 ppm

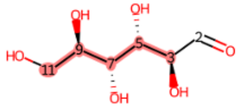

S62

Rank: 12 MW: 180.16
LTS0276202
Score: 0.83 (5/6 C)
Deviation : 0.88 ppm

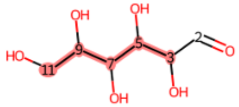

S63

Rank: 13 MW: 180.16
LTS0241274
Score: 0.83 (5/6 C)
Deviation : 1.18 ppm

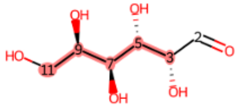

S64

Rank: 14 MW: 180.16
LTS0128031
Score: 0.83 (5/6 C)
Deviation : 1.18 ppm

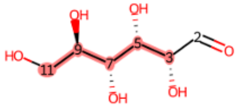

S65

Rank: 15 MW: 180.16
LTS0262158
Score: 0.83 (5/6 C)
Deviation : 1.39 ppm

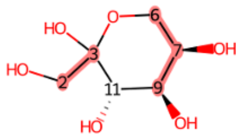

S66

Rank: 16 MW: 180.16
LTS0259277
Score: 0.83 (5/6 C)
Deviation : 1.58 ppm

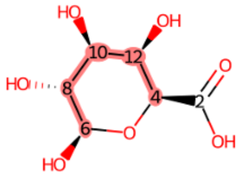

S67

Rank: 17 MW: 194.14
LTS0271461
Score: 0.83 (5/6 C)
Deviation : 2.62 ppm

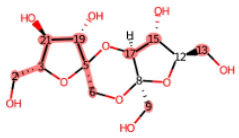

S68

Rank: 18 MW: 324.28
LTS0192374
Score: 0.83 (10/12 C)
Deviation : 2.64 ppm

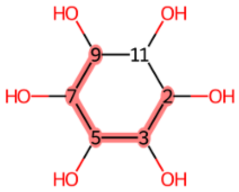

S69

Rank: 19 MW: 180.16
LTS0047771
Score: 0.83 (5/6 C)
Deviation : 3.87 ppm

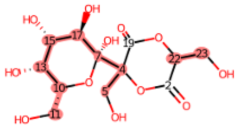

S70

Rank: 20 MW: 354.26
LTS0110247
Score: 0.83 (10/12 C)
Deviation : 4.1 ppm

Figure S12. Dereplication analysis from MixONat, structure of top 50 metabolites: compounds S**16**–S**20** from DB2

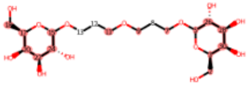

S71

Rank: 21 MW: 458.46
LTS0157972
Score: 0.83 (15/18 C)
Deviation : 7.91 ppm

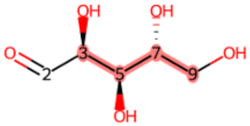

S72

Rank: 22 MW: 150.13
LTS0085513
Score: 0.8 (4/5 C)
Deviation : 0.27 ppm

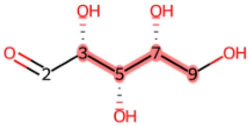

S73

Rank: 23 MW: 150.13
LTS0269907
Score: 0.8 (4/5 C)
Deviation : 1.01 ppm

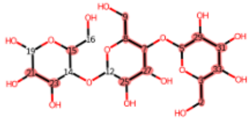

S74

Rank: 24 MW: 504.44
LTS0110579
Score: 0.78 (14/18 C)
Deviation : 3.05 ppm

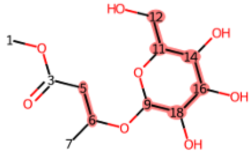

S75

Rank: 25 MW: 280.27
LTS0143481
Score: 0.73 (8/11 C)
Deviation : 1.94 ppm

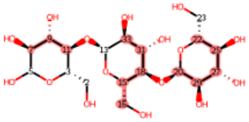

S76

Rank: 26 MW: 504.44
LTS0137131
Score: 0.72 (13/18 C)
Deviation : 3.68 ppm

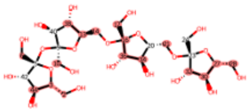

S77

Rank: 27 MW: 666.58
LTS0247235
Score: 0.71 (17/24 C)
Deviation : 6.38 ppm

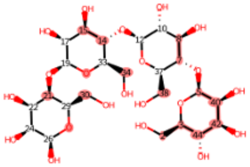

S78

Rank: 28 MW: 666.58
LTS0196709
Score: 0.7 (16/23 C)
Deviation : 5.73 ppm

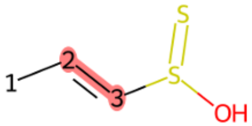

S79

Rank: 29 MW: 122.21
LTS0204729
Score: 0.67 (2/3 C)
Deviation : 0.26 ppm

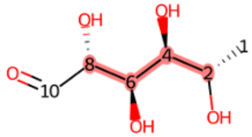

S80

Rank: 30 MW: 164.16
LTS0247365
Score: 0.67 (4/6 C)
Deviation : 1.29 ppm

Figure S13. Dereplication analysis from MixONat, structure of top 50 metabolites: compounds S**21**–S**30** from DB2

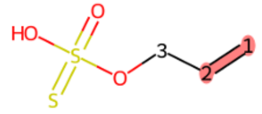

S81

Rank: 31 MW: 154.21
LTS0262385
Score: 0.67 (2/3 C)
Deviation : 1.39 ppm

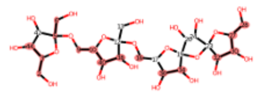

S82

Rank: 32 MW: 666.58
LTS0216911
Score: 0.67 (16/24 C)
Deviation : 7.46 ppm

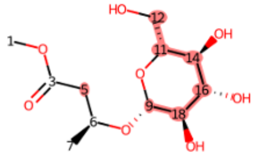

S83

Rank: 33 MW: 280.27
LTS0145917
Score: 0.64 (7/11 C)
Deviation : 2.86 ppm

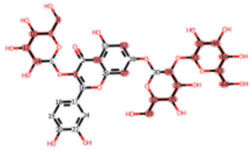

S84

Rank: 34 MW: 788.66
LTS0188265
Score: 0.61 (20/33 C)
Deviation : 8.47 ppm

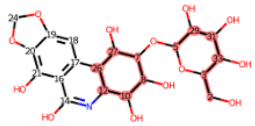

S85

Rank: 35 MW: 487.41
LTS0015975
Score: 0.6 (12/20 C)
Deviation : 3.93 ppm

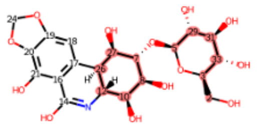

S86

Rank: 36 MW: 487.41
LTS0143193
Score: 0.6 (12/20 C)
Deviation : 4.03 ppm

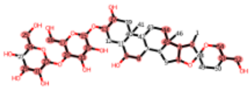

S87

Rank: 37 MW: 788.92
LTS0012525
Score: 0.59 (23/39 C)
Deviation : 7.61 ppm

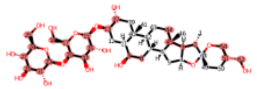

S88

Rank: 38 MW: 788.92
LTS0261262
Score: 0.59 (23/39 C)
Deviation : 8.32 ppm

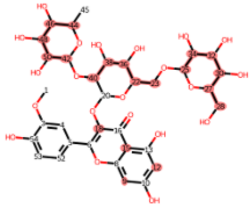

S89

Rank: 39 MW: 786.69
LTS0031032
Score: 0.59 (20/34 C)
Deviation : 7.69 ppm

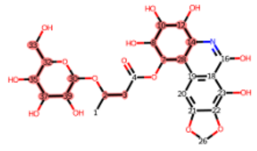

S90

Rank: 40 MW: 573.5
LTS0222933
Score: 0.58 (14/24 C)
Deviation : 4.81 ppm

Figure S14. Dereplication analysis from MixONat, structure of top 50 metabolites: compounds S**31**–S**40** from DB2

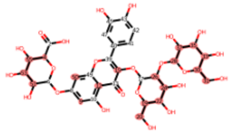

S91

Rank: 41 MW: 802.64
LTS0208698
Score: 0.58 (19/33 C)
Deviation : 5.65 ppm

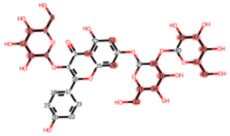

S92

Rank: 42 MW: 772.66
LTS0081415
Score: 0.58 (19/33 C)
Deviation : 6.61 ppm

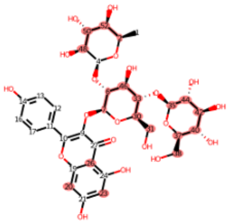

S93

Rank: 43 MW: 756.66
LTS0074468
Score: 0.58 (19/33 C)
Deviation : 8.76 ppm

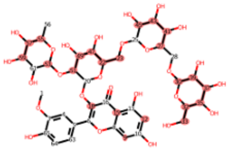

S94

Rank: 44 MW: 948.83
LTS0257423
Score: 0.57 (23/40 C)
Deviation : 8.49 ppm

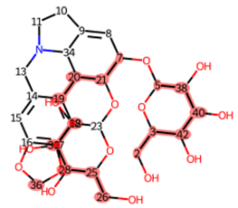

S95

Rank: 45 MW: 611.59
LTS0060212
Score: 0.57 (16/28 C)
Deviation : 6.51 ppm

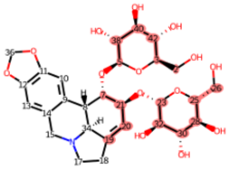

S96

Rank: 46 MW: 611.59
LTS0160600
Score: 0.57 (16/28 C)
Deviation : 7.52 ppm

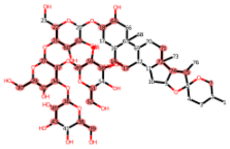

S97

Rank: 47 MW: 1097.2
LTS0129228
Score: 0.57 (29/51 C)
Deviation : 11.32 ppm

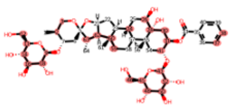

S98

Rank: 48 MW: 909.02
LTS0080305
Score: 0.57 (26/46 C)
Deviation : 12.52 ppm

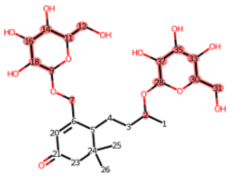

S99

Rank: 49 MW: 550.59
LTS0235000
Score: 0.56 (14/25 C)
Deviation : 5.46 ppm

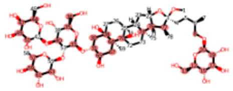

S100

Rank: 50 MW: 1129.24
LTS0043591
Score: 0.56 (29/52 C)
Deviation : 11.78 ppm

Figure S15. Dereplication analysis from MixONat, structure of top 50 metabolites: compounds S**41**–S**50** from DB2

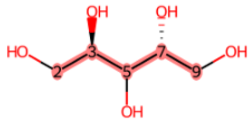

S101

Rank: 1 MW: 152.15
LTS0087699
Score: 1.0 (5/5 C)
Deviation : 0.51 ppm

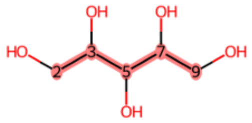

S102

Rank: 2 MW: 152.15
LTS0023185
Score: 1.0 (5/5 C)
Deviation : 0.51 ppm

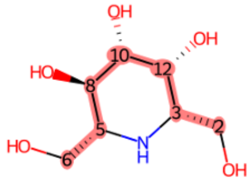

S103

Rank: 3 MW: 193.2
LTS0231470
Score: 1.0 (7/7 C)
Deviation : 1.13 ppm

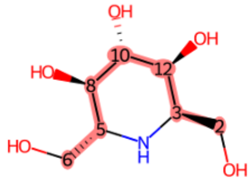

S104

Rank: 4 MW: 193.2
LTS0161003
Score: 1.0 (7/7 C)
Deviation : 1.19 ppm

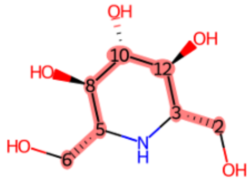

S105

Rank: 5 MW: 193.2
LTS0087238
Score: 1.0 (7/7 C)
Deviation : 1.51 ppm

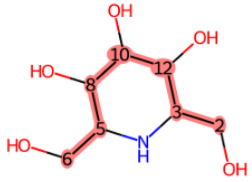

S106

Rank: 6 MW: 193.2
LTS0263530
Score: 1.0 (7/7 C)
Deviation : 1.75 ppm

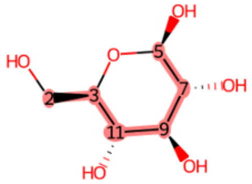

S107

Rank: 7 MW: 180.16
LTS0107522
Score: 1.0 (6/6 C)
Deviation : 1.81 ppm

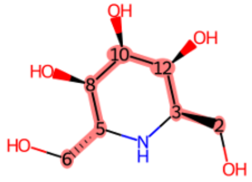

S108

Rank: 8 MW: 193.2
LTS0204783
Score: 1.0 (7/7 C)
Deviation : 2.07 ppm

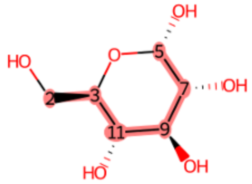

S109

Rank: 9 MW: 180.16
LTS0132398
Score: 1.0 (6/6 C)
Deviation : 2.11 ppm

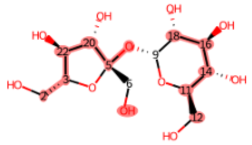

S110

Rank: 10 MW: 342.3
LTS0272557
Score: 1.0 (12/12 C)
Deviation : 2.12 ppm

Figure S16. Dereplication analysis from MixONat, structure of top 50 metabolites: compounds S**1**–S**10** from DB3

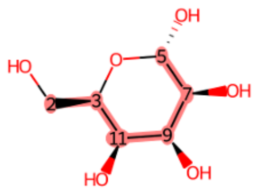

S111

Rank: 11 MW: 180.16
LTS0089262
Score: 1.0 (6/6 C)
Deviation : 2.2 ppm

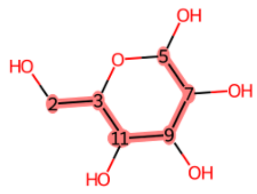

S112

Rank: 12 MW: 180.16
LTS0231627
Score: 1.0 (6/6 C)
Deviation : 2.2 ppm

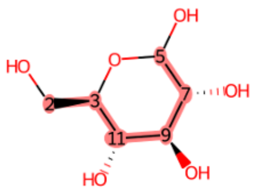

S113

Rank: 13 MW: 180.16
LTS0013597
Score: 1.0 (6/6 C)
Deviation : 2.2 ppm

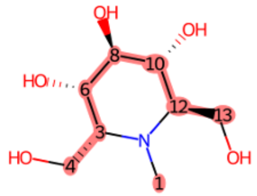

S114

Rank: 14 MW: 207.22
LTS0017054
Score: 1.0 (8/8 C)
Deviation : 2.47 ppm

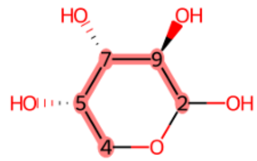

S115

Rank: 15 MW: 150.13
LTS0043712
Score: 1.0 (5/5 C)
Deviation : 2.48 ppm

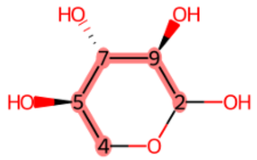

S116

Rank: 16 MW: 150.13
LTS0132381
Score: 1.0 (5/5 C)
Deviation : 2.48 ppm

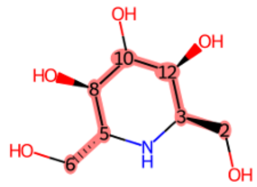

S117

Rank: 17 MW: 193.2
LTS0019868
Score: 1.0 (7/7 C)
Deviation : 2.52 ppm

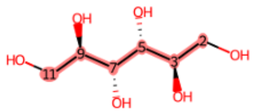

S118

Rank: 18 MW: 182.17
LTS0199986
Score: 1.0 (6/6 C)
Deviation : 2.78 ppm

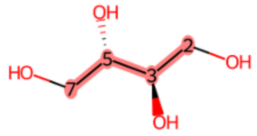

S119

Rank: 19 MW: 122.12
LTS0150163
Score: 1.0 (4/4 C)
Deviation : 2.82 ppm

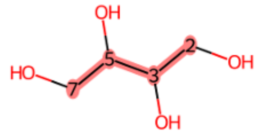

S120

Rank: 20 MW: 122.12
LTS0003058
Score: 1.0 (4/4 C)
Deviation : 2.82 ppm

Figure S17. Dereplication analysis from MixONat, structure of top 50 metabolites: compounds S**16**–S**20** from DB3

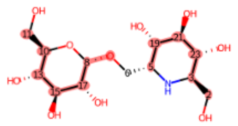

S121

Rank: 21 MW: 355.34
LTS0192514
Score: 1.0 (13/13 C)
Deviation : 3.61 ppm

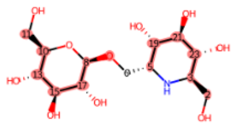

S122

Rank: 22 MW: 355.34
LTS0247838
Score: 1.0 (13/13 C)
Deviation : 4.0 ppm

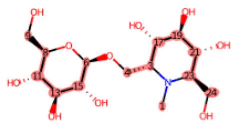

S123

Rank: 23 MW: 369.37
LTS0139126
Score: 1.0 (14/14 C)
Deviation : 4.49 ppm

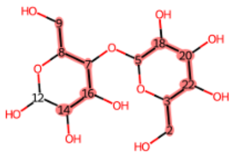

S124

Rank: 24 MW: 342.3
LTS0210079
Score: 0.92 (11/12 C)
Deviation : 2.83 ppm

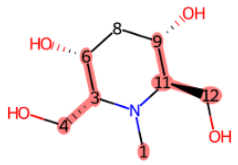

S125

Rank: 25 MW: 191.23
LTS0100655
Score: 0.88 (7/8 C)
Deviation : 1.53 ppm

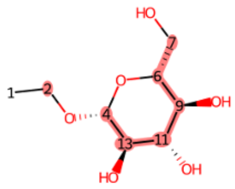

S126

Rank: 26 MW: 208.21
LTS0141759
Score: 0.88 (7/8 C)
Deviation : 3.07 ppm

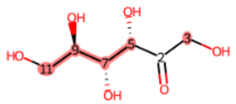

S127

Rank: 27 MW: 180.16
LTS0241114
Score: 0.83 (5/6 C)
Deviation : 0.8 ppm

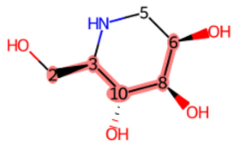

S128

Rank: 28 MW: 163.17
LTS0245637
Score: 0.83 (5/6 C)
Deviation : 1.1 ppm

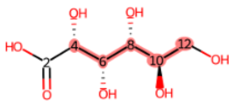

S129

Rank: 29 MW: 196.16
LTS0057096
Score: 0.83 (5/6 C)
Deviation : 1.16 ppm

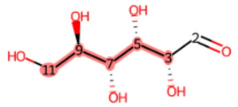

S130

Rank: 30 MW: 180.16
LTS0262158
Score: 0.83 (5/6 C)
Deviation : 1.39 ppm

Figure S18. Dereplication analysis from MixONat, structure of top 50 metabolites: compounds S**21**–S**30** from DB3

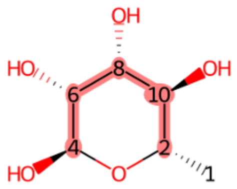

S131

Rank: 31 MW: 164.16
LTS0084516
Score: 0.83 (5/6 C)
Deviation : 1.41 ppm

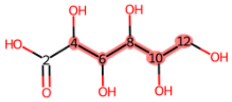

S132

Rank: 32 MW: 196.16
LTS0223750
Score: 0.83 (5/6 C)
Deviation : 1.48 ppm

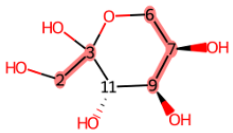

S133

Rank: 33 MW: 180.16
LTS0259277
Score: 0.83 (5/6 C)
Deviation : 1.58 ppm

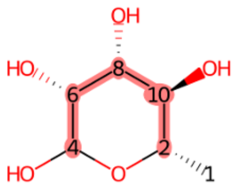

S134

Rank: 34 MW: 164.16
LTS0239546
Score: 0.83 (5/6 C)
Deviation : 1.95 ppm

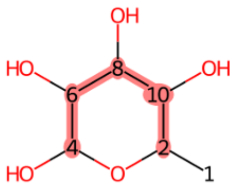

S135

Rank: 35 MW: 164.16
LTS0175812
Score: 0.83 (5/6 C)
Deviation : 2.09 ppm

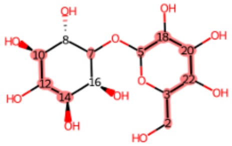

S136

Rank: 36 MW: 342.3
LTS0080274
Score: 0.83 (10/12 C)
Deviation : 2.11 ppm

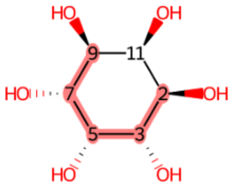

S137

Rank: 37 MW: 180.16
LTS0165930
Score: 0.83 (5/6 C)
Deviation : 2.45 ppm

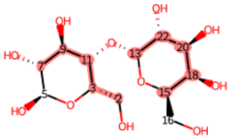

S138

Rank: 38 MW: 342.3
LTS0220516
Score: 0.83 (10/12 C)
Deviation : 2.49 ppm

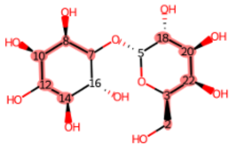

S139

Rank: 39 MW: 342.3
LTS0188871
Score: 0.83 (10/12 C)
Deviation : 3.8 ppm

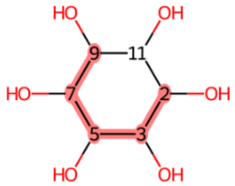

S140

Rank: 40 MW: 180.16
LTS0047771
Score: 0.83 (5/6 C)
Deviation : 3.87 ppm

Figure S19. Dereplication analysis from MixONat, structure of top 50 metabolites: compounds S**31**–S**40** from DB3

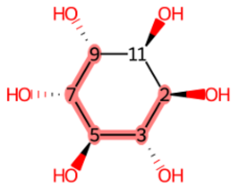

S141

Rank: 41 MW: 180.16
LTS0257088
Score: 0.83 (5/6 C)
Deviation : 3.87 ppm

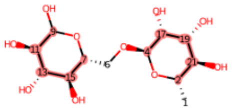

S142

Rank: 42 MW: 326.3
LTS0169607
Score: 0.83 (10/12 C)
Deviation : 4.99 ppm

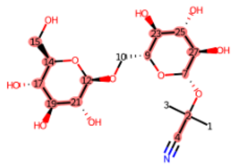

S143

Rank: 43 MW: 409.39
LTS0023485
Score: 0.81 (13/16 C)
Deviation : 3.11 ppm

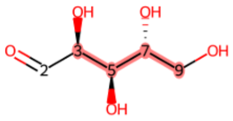

S144

Rank: 44 MW: 150.13
LTS0085513
Score: 0.8 (4/5 C)
Deviation : 0.27 ppm

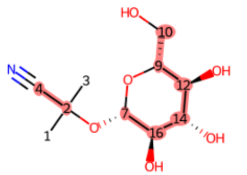

S145

Rank: 45 MW: 247.25
LTS0206216
Score: 0.8 (8/10 C)
Deviation : 1.73 ppm

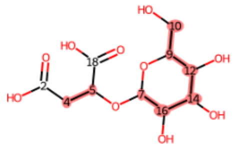

S146

Rank: 46 MW: 296.23
LTS0237794
Score: 0.8 (8/10 C)
Deviation : 1.78 ppm

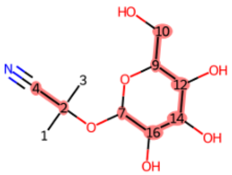

S147

Rank: 47 MW: 247.25
LTS0032647
Score: 0.8 (8/10 C)
Deviation : 1.78 ppm

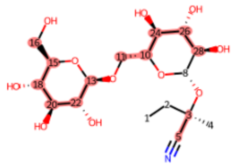

S148

Rank: 48 MW: 423.41
LTS0153167
Score: 0.76 (13/17 C)
Deviation : 2.25 ppm

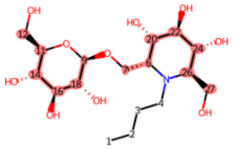

S149

Rank: 49 MW: 411.45
LTS0109641
Score: 0.76 (13/17 C)
Deviation : 4.97 ppm

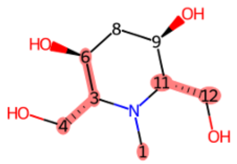

S150

Rank: 50 MW: 191.23
LTS0104449
Score: 0.75 (6/8 C)
Deviation : 1.25 ppm

Figure S20. Dereplication analysis from MixONat, structure of top 50 metabolites: compounds S**41**–S**50** from DB3

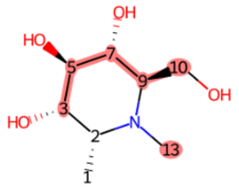

S151

Rank: 51 MW: 191.23
LTS0078874
Score: 0.75 (6/8 C)
Deviation : 2.27 ppm

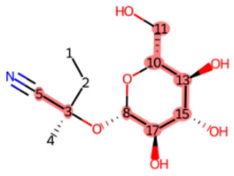

S152

Rank: 52 MW: 261.27
LTS0126731
Score: 0.73 (8/11 C)
Deviation : 1.01 ppm

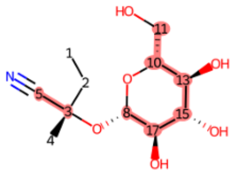

S153

Rank: 53 MW: 261.27
LTS0070973
Score: 0.73 (8/11 C)
Deviation : 1.01 ppm

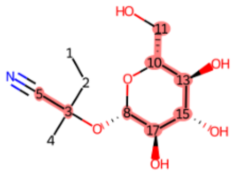

S154

Rank: 54 MW: 261.27
LTS0184222
Score: 0.73 (8/11 C)
Deviation : 1.3 ppm

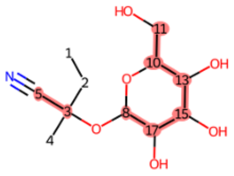

S155

Rank: 55 MW: 261.27
LTS0211301
Score: 0.73 (8/11 C)
Deviation : 1.35 ppm

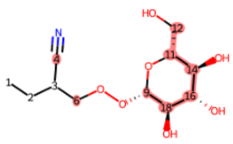

S156

Rank: 56 MW: 277.27
LTS0178194
Score: 0.73 (8/11 C)
Deviation : 1.98 ppm

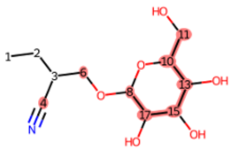

S157

Rank: 57 MW: 261.27
LTS0045615
Score: 0.73 (8/11 C)
Deviation : 3.09 ppm

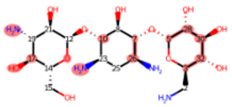

S158

Rank: 58 MW: 484.5
LTS0046726
Score: 0.72 (13/18 C)
Deviation : 3.9 ppm

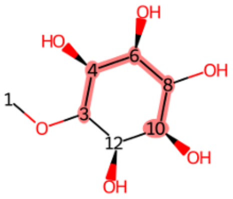

S159

Rank: 59 MW: 194.18
LTS0013262
Score: 0.71 (5/7 C)
Deviation : 0.58 ppm

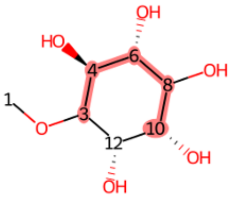

S160

Rank: 60 MW: 194.18
LTS0065138
Score: 0.71 (5/7 C)
Deviation : 0.58 ppm

Figure S21. Dereplication analysis from MixONat, structure of top 50 metabolites: compounds S**51**–S**60** from DB3

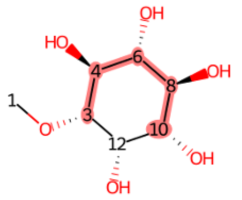

S161

Rank: 61 MW: 194.18
LTS0249934
Score: 0.71 (5/7 C)
Deviation : 0.66 ppm

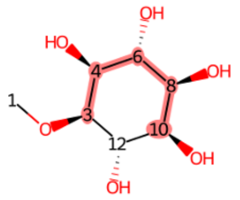

S162

Rank: 62 MW: 194.18
LTS0051765
Score: 0.71 (5/7 C)
Deviation : 0.66 ppm

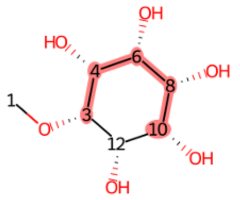

S163

Rank: 63 MW: 194.18
LTS0220361
Score: 0.71 (5/7 C)
Deviation : 0.87 ppm

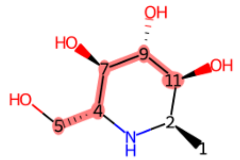

S164

Rank: 64 MW: 177.2
LTS0045051
Score: 0.71 (5/7 C)
Deviation : 0.91 ppm

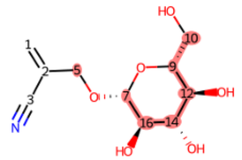

S165

Rank: 65 MW: 245.23
LTS0013020
Score: 0.7 (7/10 C)
Deviation : 1.95 ppm

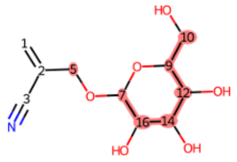

S166

Rank: 66 MW: 245.23
LTS0272370
Score: 0.7 (7/10 C)
Deviation : 2.04 ppm

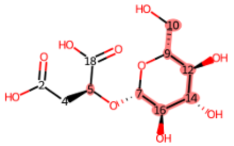

S167

Rank: 67 MW: 296.23
LTS0081012
Score: 0.7 (7/10 C)
Deviation : 2.89 ppm

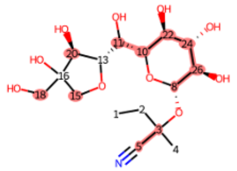

S168

Rank: 68 MW: 393.39
LTS0167176
Score: 0.69 (11/16 C)
Deviation : 2.56 ppm

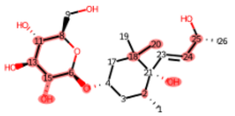

S169

Rank: 69 MW: 390.47
LTS0189590
Score: 0.68 (13/19 C)
Deviation : 5.74 ppm

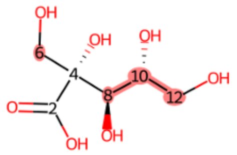

S170

Rank: 70 MW: 196.16
LTS0056947
Score: 0.67 (4/6 C)
Deviation : 0.96 ppm

Figure S22. Dereplication analysis from MixONat, structure of top 50 metabolites: compounds S**61**–S**70** from DB3

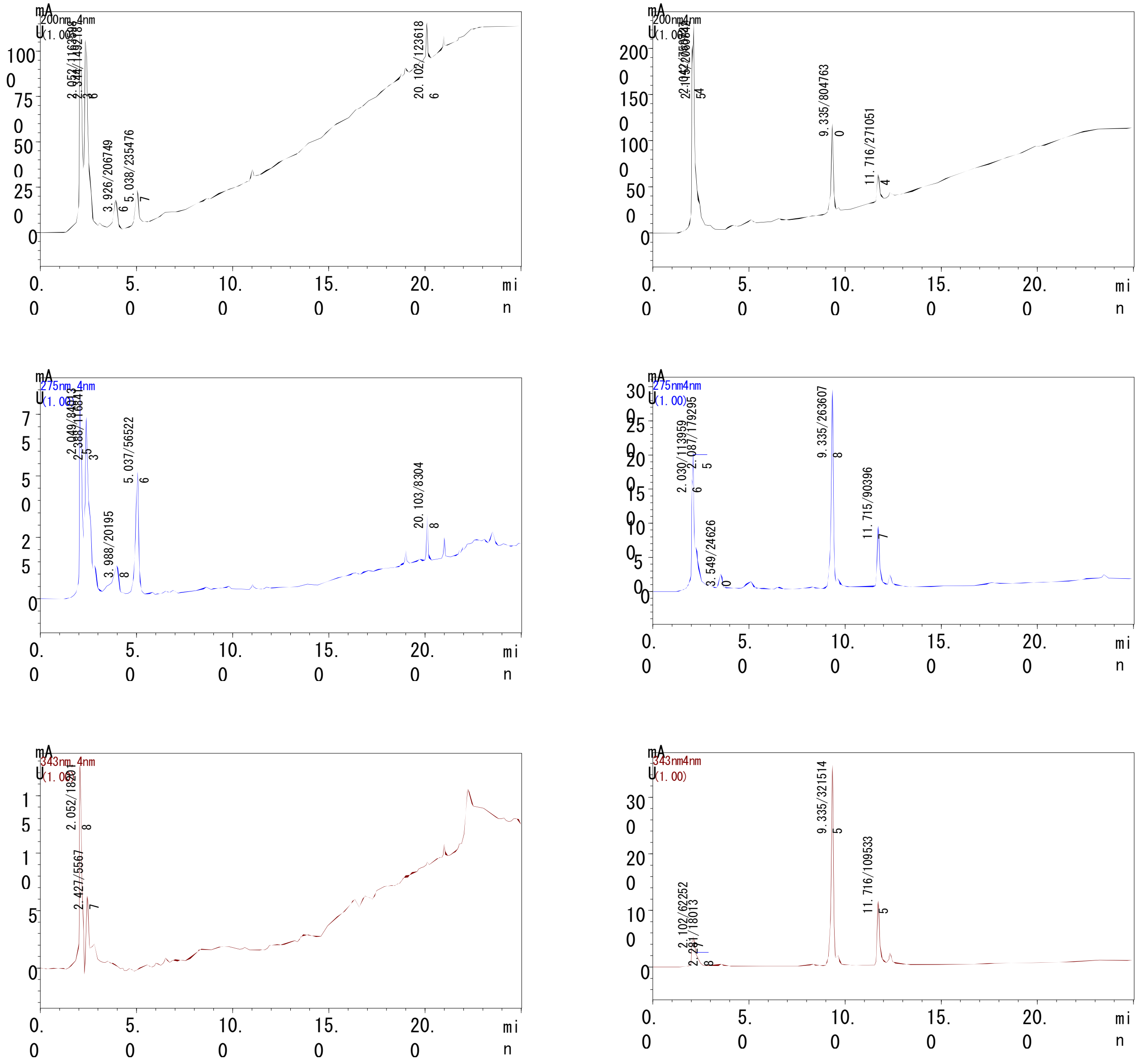

Figure S23. HPLC spectra of AL1 and AL6 (Left: AL1, Right: AL6).

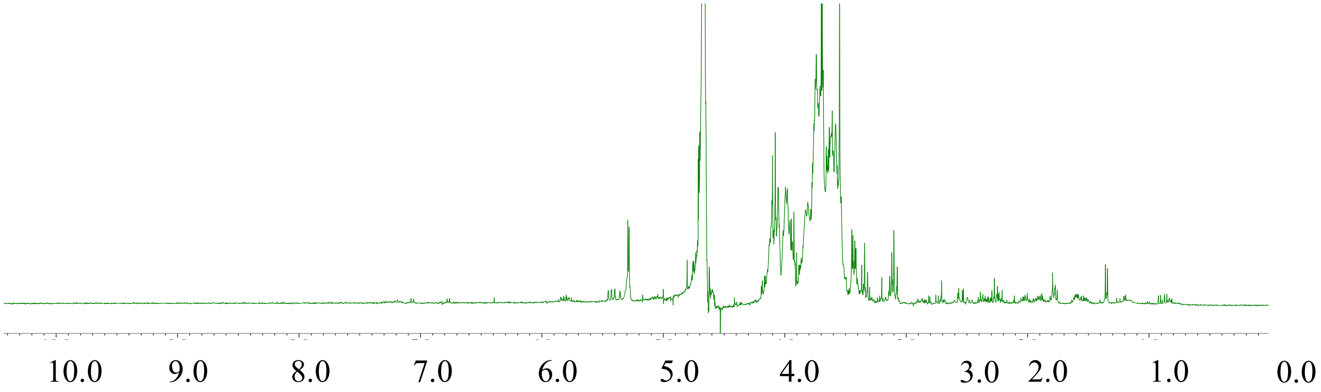

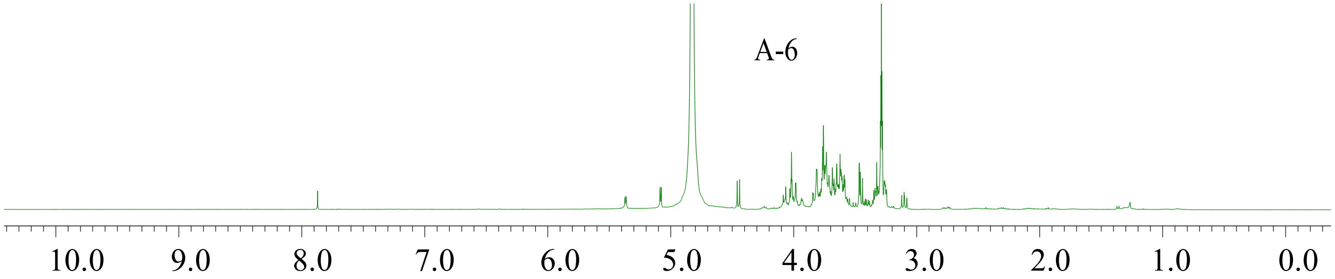

Figure S24. NMR profile of AL1 and AL6.

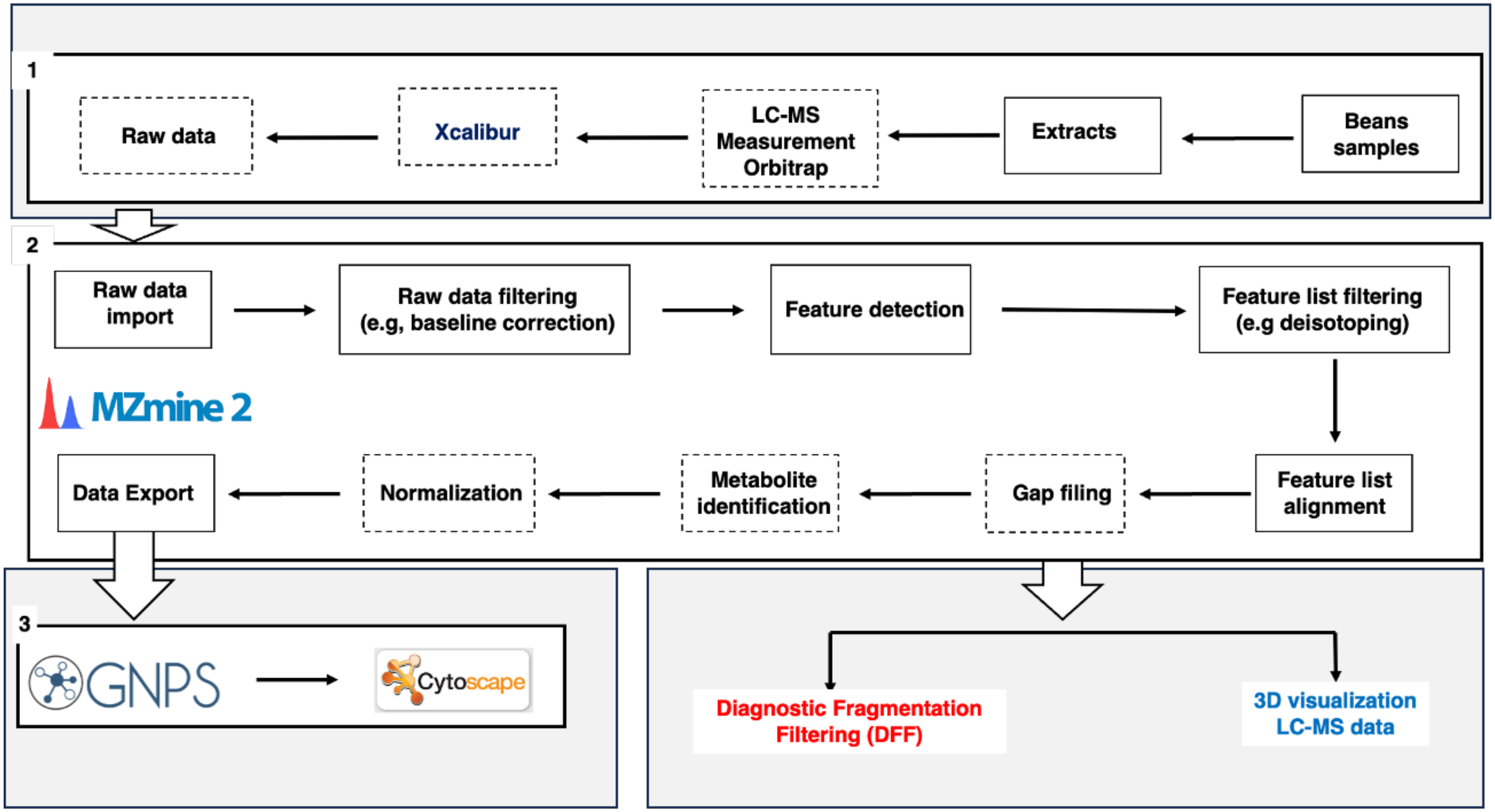

Figure S25. Mixture analysis LC-MS/MS experimental flow

A. Results of MixONat from Allium DB1

Allowed margin : 1.3
No molecular weight information
Number of results : 50
Equivalent carbons not allowed

Rank: 1
ID : 580
Name : LTS0155285
CAS : nan
MW : 92.09
Score : 1.0 (3/3 carbons)
Cumulated absolute difference : 1.04
Spectrum shifts left : 44/47

Matched spectrum shifts :
64.41 65.15 73.80
Matched SDF shifts :
64.90 64.90 74.10
Matched spectrum intensities :
0.020000 0.010000 0.020000

Rank: 2
ID : 98
Name : LTS0272557
CAS : nan
MW : 342.3
Score : 1.0 (12/12 carbons)
Cumulated absolute difference : 2.12
Spectrum shifts left : 35/47

Matched spectrum shifts :
61.91 63.59 64.35 70.63 72.01 73.41 74.30 75.71 78.19 83.32 93.67 105.47
Matched SDF shifts :
61.80 63.70 64.30 70.20 72.00 73.60 74.40 75.80 78.20 83.10 92.90 105.50
Matched spectrum intensities :
0.010000 0.010000 0.020000 0.050000 0.020000 0.020000 0.020000 0.010000 0.060000 0.020000 0.020000 0.010000

Rank: 3
ID : 269
Name : LTS0013597
CAS : nan
MW : 180.16
Score : 1.0 (6/6 carbons)
Cumulated absolute difference : 2.20
Spectrum shifts left : 41/47

Matched spectrum shifts :
61.91 71.26 72.01 73.08 73.41 93.67
Matched SDF shifts :
61.60 71.10 72.40 72.80 73.50 92.70
Matched spectrum intensities :
0.010000 0.010000 0.020000 0.010000 0.020000 0.020000

Rank: 4
ID : 585
Name : LTS0199986
CAS : nan
MW : 182.17
Score : 1.0 (6/6 carbons)
Cumulated absolute difference : 2.78
Spectrum shifts left : 41/47

Matched spectrum shifts :
65.15 65.79 73.08 73.41 74.30 74.51
Matched SDF shifts :
66.30 66.30 73.40 73.40 74.80 74.80
Matched spectrum intensities :
0.010000 0.040000 0.010000 0.020000 0.020000 0.020000

Rank: 5
ID : 535
Name : LTS0150163
CAS : nan
MW : 122.12
Score : 1.0 (4/4 carbons)
Cumulated absolute difference : 2.82
Spectrum shifts left : 43/47

Matched spectrum shifts :
65.15 65.79 75.71 76.25
Matched SDF shifts :
66.40 66.40 75.50 75.50
Matched spectrum intensities :
0.010000 0.040000 0.010000 0.030000

Rank: 6
ID : 292
Name : LTS0113066
CAS : nan
MW : 504.44
Score : 1.0 (18/18 carbons)
Cumulated absolute difference : 4.29
Spectrum shifts left : 29/47

Matched spectrum shifts :
62.64 63.59 64.35 69.21 71.71 72.01 73.41 73.80 74.30 74.51 75.71 76.25 77.22 78.19 83.32 93.67 103.43 105.47

Matched SDF shifts :
62.80 63.70 64.30 68.60 71.70 72.10 73.70 73.90 74.10 75.40 75.80 76.30 77.30 78.20 83.10 94.80 103.60 105.50
Matched spectrum intensities :
0.050000 0.010000 0.020000 0.050000 0.030000 0.020000 0.020000 0.020000 0.020000 0.020000 0.010000 0.030000 0.010000 0.060000 0.020000 0.020000 0.020000 0.010000

Rank: 7
ID : 530
Name : LTS0146582
CAS : nan
MW : 318.3
Score : 0.89 (8/9 carbons)
Cumulated absolute difference : 3.66
Spectrum shifts left : 39/47

Matched spectrum shifts :
40.05 63.49 69.21 71.26 72.01 73.41 73.80 105.47
Matched SDF shifts :
39.10 63.30 68.50 71.10 72.40 73.60 73.70 104.50
Matched spectrum intensities :
0.010000 0.010000 0.050000 0.010000 0.020000 0.020000 0.020000 0.010000
Not matched SDF shifts :
80.60

Rank: 8
ID : 711
Name : LTS0183884
CAS : nan
MW : 666.58
Score : 0.88 (21/24 carbons)
Cumulated absolute difference : 4.74
Spectrum shifts left : 26/47

Matched spectrum shifts :
62.64 63.59 64.35 69.21 71.32 71.71 72.01 73.41 73.80 74.30 74.51 75.71 76.25 77.11 77.22 78.19 78.46 83.32 93.67 103.43 105.47
Matched SDF shifts :
62.80 63.70 64.30 68.60 71.70 71.80 72.10 73.70 73.90 74.10 73.90 75.80 76.30 77.00 77.30 78.20 78.30 83.10 94.80 103.60 105.50
Matched spectrum intensities :
0.050000 0.010000 0.020000 0.050000 0.050000 0.030000 0.020000 0.020000 0.020000 0.020000 0.020000 0.010000 0.030000 0.010000 0.010000 0.060000 0.010000 0.020000 0.020000 0.020000 0.010000
Not matched SDF shifts :
67.30 75.40 103.80

Rank: 9
ID : 950
Name : LTS0062089
CAS : nan
MW : 666.58
Score : 0.88 (21/24 carbons)
Cumulated absolute difference : 4.74
Spectrum shifts left : 26/47

Matched spectrum shifts :
62.64 63.59 64.35 69.21 71.32 71.71 72.01 73.41 73.80 74.30 74.51 75.71 76.25 77.11 77.22 78.19 78.46 83.32 93.67 103.43 105.47
Matched SDF shifts :
62.80 63.70 64.30 68.60 71.70 71.80 72.10 73.70 73.90 74.10 73.90 75.80 76.30 77.00 77.30 78.20 78.30 83.10 94.80 103.60 105.50
Matched spectrum intensities :
0.050000 0.010000 0.020000 0.050000 0.050000 0.030000 0.020000 0.020000 0.020000 0.020000 0.020000 0.010000 0.030000 0.010000 0.010000 0.060000 0.010000 0.020000 0.020000 0.020000 0.010000
Not matched SDF shifts :
67.30 75.40 103.80

Rank: 10
ID : 241

Name : LTS0241114
CAS : nan
MW : 180.16
Score : 0.83 (5/6 carbons)
Cumulated absolute difference : 0.80
Spectrum shifts left : 42/47

Matched spectrum shifts :
64.35 65.79 73.08 75.71 76.25
Matched SDF shifts :
64.40 66.20 73.00 75.50 76.20
Matched spectrum intensities :
0.020000 0.040000 0.010000 0.010000 0.030000
Not matched SDF shifts :
217.40

Rank: 11
ID : 109
Name : LTS0276202
CAS : nan
MW : 180.16
Score : 0.83 (5/6 carbons)
Cumulated absolute difference : 0.88
Spectrum shifts left : 42/47

Matched spectrum shifts :
65.79 73.08 73.41 74.51 76.25
Matched SDF shifts :
66.30 73.10 73.40 74.80 76.20
Matched spectrum intensities :
0.040000 0.010000 0.020000 0.020000 0.030000
Not matched SDF shifts :
200.80

Rank: 12
ID : 124
Name : LTS0128031
CAS : nan
MW : 180.16
Score : 0.83 (5/6 carbons)
Cumulated absolute difference : 1.18
Spectrum shifts left : 42/47

Matched spectrum shifts :
65.79 70.63 73.41 73.80 74.51
Matched SDF shifts :
66.30 70.70 73.40 73.50 74.80
Matched spectrum intensities :
0.040000 0.050000 0.020000 0.020000 0.020000
Not matched SDF shifts :
200.80

Rank: 13
ID : 243
Name : LTS0241274
CAS : nan
MW : 180.16
Score : 0.83 (5/6 carbons)
Cumulated absolute difference : 1.18
Spectrum shifts left : 42/47

Matched spectrum shifts :
65.79 71.32 72.01 73.41 74.51
Matched SDF shifts :
66.30 71.50 72.20 73.40 74.80
Matched spectrum intensities :
0.040000 0.050000 0.020000 0.020000 0.020000
Not matched SDF shifts :
201.30

Rank: 14
ID : 66
Name : LTS0262158
CAS : nan
MW : 180.16
Score : 0.83 (5/6 carbons)
Cumulated absolute difference : 1.39
Spectrum shifts left : 42/47

Matched spectrum shifts :
65.79 73.08 73.41 73.80 74.51
Matched SDF shifts :
66.30 72.80 73.40 73.50 74.80
Matched spectrum intensities :
0.040000 0.010000 0.020000 0.020000 0.020000
Not matched SDF shifts :
200.80

Rank: 15
ID : 305
Name : LTS0259277
CAS : nan
MW : 180.16
Score : 0.83 (5/6 carbons)
Cumulated absolute difference : 1.58
Spectrum shifts left : 42/47

Matched spectrum shifts :
63.49 63.59 69.21 70.63 98.34
Matched SDF shifts :
63.30 63.80 69.20 69.70 98.10
Matched spectrum intensities :
0.010000 0.010000 0.050000 0.050000 0.030000
Not matched SDF shifts :

67.50

Rank: 16
ID : 722
Name : LTS0271461
CAS : nan
MW : 194.14
Score : 0.83 (5/6 carbons)
Cumulated absolute difference : 2.62
Spectrum shifts left : 42/47

Matched spectrum shifts :
72.01 73.08 73.41 75.71 93.67
Matched SDF shifts :
72.30 73.00 72.80 75.30 94.90
Matched spectrum intensities :
0.020000 0.010000 0.020000 0.010000 0.020000
Not matched SDF shifts :
172.90

Rank: 17
ID : 518
Name : LTS0047771
CAS : nan
MW : 180.16
Score : 0.83 (5/6 carbons)
Cumulated absolute difference : 3.87
Spectrum shifts left : 42/47

Matched spectrum shifts :
71.71 72.01 73.08 73.41 73.80
Matched SDF shifts :
72.70 72.70 72.70 72.70 72.70
Matched spectrum intensities :

0.030000   0.020000   0.010000   0.020000   0.020000
Not matched SDF shifts :
72.70

Rank: 18
ID : 487
Name : LTS0110247
CAS : nan
MW : 354.26
Score : 0.83 (10/12 carbons)
Cumulated absolute difference : 4.10
Spectrum shifts left : 37/47

Matched spectrum shifts :
61.91   63.49   65.79   71.26   73.08   74.30   75.71   76.25   76.75   98.34
Matched SDF shifts :
60.80   63.20   66.60   71.20   72.80   74.20   75.70   76.20   75.90   97.80
Matched spectrum intensities :
0.010000   0.010000   0.040000   0.010000   0.010000   0.020000   0.010000   0.030000   0.020000   0.030000
Not matched SDF shifts :
168.60   170.40

Rank: 19
ID : 451
Name : LTS0157972
CAS : nan
MW : 458.46
Score : 0.83 (15/18 carbons)
Cumulated absolute difference : 7.91
Spectrum shifts left : 32/47

Matched spectrum shifts :
62.64   63.49   65.15   65.79   69.21   71.26   71.32   71.71   72.01   74.51   75.71   78.19   78.46   103.43   105.47
Matched SDF shifts :

62.80 62.80 66.40 66.40 68.80 71.50 71.50 72.20 72.20 75.10 75.10 78.10 78.10 104.40 104.40
Matched spectrum intensities :
0.050000 0.010000 0.010000 0.040000 0.050000 0.010000 0.050000 0.030000 0.020000 0.020000 0.010000 0.060000 0.010000 0.020000 0.010000
Not matched SDF shifts :
30.20 30.20 68.80

Rank: 20
ID : 404
Name : LTS0085513
CAS : nan
MW : 150.13
Score : 0.8 (4/5 carbons)
Cumulated absolute difference : 0.27
Spectrum shifts left : 43/47

Matched spectrum shifts :
64.35 73.08 73.41 76.25
Matched SDF shifts :
64.40 73.00 73.50 76.20
Matched spectrum intensities :
0.020000 0.010000 0.020000 0.030000
Not matched SDF shifts :
200.80

Rank: 21
ID : 731
Name : LTS0269907
CAS : nan
MW : 150.13
Score : 0.8 (4/5 carbons)
Cumulated absolute difference : 1.01
Spectrum shifts left : 43/47

Matched spectrum shifts :
64.35 72.01 73.08 73.41
Matched SDF shifts :
64.40 72.80 73.00 73.50
Matched spectrum intensities :
0.020000 0.020000 0.010000 0.020000
Not matched SDF shifts :
200.80

Rank: 22
ID : 901
Name : LTS0110579
CAS : nan
MW : 504.44
Score : 0.78 (14/18 carbons)
Cumulated absolute difference : 3.05
Spectrum shifts left : 33/47

Matched spectrum shifts :
61.91 62.64 69.21 72.01 73.08 73.41 73.80 74.30 74.51 77.11 78.19 78.46 78.61 103.43
Matched SDF shifts :
61.50 62.60 69.50 72.00 73.40 74.10 74.00 74.30 74.50 77.10 78.30 78.60 79.40 103.40
Matched spectrum intensities :
0.010000 0.050000 0.050000 0.020000 0.010000 0.020000 0.020000 0.020000 0.020000 0.010000 0.060000 0.010000 0.010000 0.020000
Not matched SDF shifts :
61.10 79.40 95.30 103.80

Rank: 23
ID : 1012
Name : LTS0137131
CAS : nan
MW : 504.44
Score : 0.72 (13/18 carbons)

Cumulated absolute difference : 3.68
Spectrum shifts left : 34/47

Matched spectrum shifts :
61.91 71.32 73.08 73.41 73.80 74.30 74.51 76.25 76.75 77.11 78.46 78.61 103.43
Matched SDF shifts :
61.30 71.40 73.70 73.40 74.00 74.30 74.50 76.30 77.00 77.20 79.40 79.40 103.40
Matched spectrum intensities :
0.010000 0.050000 0.010000 0.020000 0.020000 0.020000 0.020000 0.030000 0.020000 0.010000 0.010000 0.010000 0.020000
Not matched SDF shifts :
61.10 61.10 74.10 95.30 103.80

Rank: 24
ID : 873
Name : LTS0247235
CAS : nan
MW : 666.58
Score : 0.71 (17/24 carbons)
Cumulated absolute difference : 6.38
Spectrum shifts left : 30/47

Matched spectrum shifts :
61.91 62.64 63.49 63.59 64.35 64.41 64.48 74.30 74.51 75.71 76.25 78.46 78.61 83.32 83.99 84.35 105.47
Matched SDF shifts :
63.10 62.80 63.20 63.20 63.90 63.90 63.90 74.40 74.40 75.90 76.20 79.20 79.20 83.30 83.70 84.90 105.30
Matched spectrum intensities :
0.010000 0.050000 0.010000 0.010000 0.020000 0.020000 0.040000 0.020000 0.020000 0.010000 0.030000 0.010000 0.010000 0.020000
0.010000 0.010000 0.010000
Not matched SDF shifts :
63.10 80.70 80.70 84.90 105.30 107.90 107.90

Rank: 25
ID : 58
Name : LTS0204729

CAS : nan
MW : 122.21
Score : 0.67 (2/3 carbons)
Cumulated absolute difference : 0.26
Spectrum shifts left : 45/47

Matched spectrum shifts :
127.50 134.36
Matched SDF shifts :
127.50 134.10
Matched spectrum intensities :
0.010000 0.010000
Not matched SDF shifts :
16.50

Rank: 26
ID : 868
Name : LTS0247365
CAS : nan
MW : 164.16
Score : 0.67 (4/6 carbons)
Cumulated absolute difference : 1.29
Spectrum shifts left : 43/47

Matched spectrum shifts :
69.21 73.08 73.41 74.51
Matched SDF shifts :
69.00 72.80 73.10 75.00
Matched spectrum intensities :
0.050000 0.010000 0.020000 0.020000
Not matched SDF shifts :
18.90 200.80

Rank: 27
ID : 20
Name : LTS0262385
CAS : nan
MW : 154.21
Score : 0.67 (2/3 carbons)
Cumulated absolute difference : 1.39
Spectrum shifts left : 45/47

Matched spectrum shifts :
118.03 134.36
Matched SDF shifts :
117.80 133.20
Matched spectrum intensities :
0.010000 0.010000
Not matched SDF shifts :
59.00

Rank: 28
ID : 807
Name : LTS0216911
CAS : nan
MW : 666.58
Score : 0.67 (16/24 carbons)
Cumulated absolute difference : 7.46
Spectrum shifts left : 31/47

Matched spectrum shifts :
61.91 62.64 63.49 63.59 64.35 74.30 74.51 75.71 76.25 78.19 78.46 78.61 83.32 83.99 84.35 105.47
Matched SDF shifts :
62.80 62.80 63.20 63.20 63.10 74.40 74.40 76.20 76.20 79.20 79.20 79.20 83.70 83.70 84.90 105.30
Matched spectrum intensities :
0.010000 0.050000 0.010000 0.010000 0.020000 0.020000 0.020000 0.010000 0.030000 0.060000 0.010000 0.010000 0.020000 0.010000
0.010000 0.010000
Not matched SDF shifts :
63.10 63.10 63.10 79.20 84.90 105.30 105.30 105.30

Rank: 29
ID : 718
Name : LTS0188265
CAS : nan
MW : 788.66
Score : 0.61 (20/33 carbons)
Cumulated absolute difference : 8.47
Spectrum shifts left : 27/47

Matched spectrum shifts :
61.91 62.64 63.49 71.26 71.32 71.71 73.80 74.51 76.75 77.11 77.22 78.19 78.46 78.61 83.32 93.67 99.50 103.43 105.47 134.36
Matched SDF shifts :
62.50 62.60 62.60 71.20 71.10 71.60 73.90 74.50 77.10 77.80 77.50 77.90 78.80 78.70 82.80 94.70 99.10 104.50 105.60 133.10
Matched spectrum intensities :
0.010000 0.050000 0.010000 0.010000 0.050000 0.030000 0.020000 0.020000 0.020000 0.010000 0.010000 0.060000 0.010000 0.010000 0.020000 0.020000 0.050000 0.020000 0.010000 0.010000
Not matched SDF shifts :
100.40 101.10 115.50 115.60 121.30 121.60 145.20 148.80 156.00 156.50 160.80 162.60 177.60

Rank: 30
ID : 956
Name : LTS0012525
CAS : nan
MW : 788.92
Score : 0.59 (23/39 carbons)
Cumulated absolute difference : 7.61
Spectrum shifts left : 24/47

Matched spectrum shifts :
40.05 40.69 40.90 41.33 41.55 61.91 62.64 63.59 64.35 64.41 69.21 70.63 72.01 73.41 74.51 77.11 78.19 78.46 78.61 83.32 84.35 103.43 105.47
Matched SDF shifts :

39.30 40.80 41.00 42.60 41.80 61.50 62.60 63.90 64.40 64.70 69.30 70.60 72.00 73.40 74.50 77.10 78.30 78.60 79.40 82.20
85.10 102.80 105.80
Matched spectrum intensities :
0.010000 0.880000 1.000000 0.440000 0.140000 0.010000 0.050000 0.010000 0.020000 0.020000 0.050000 0.050000 0.020000 0.020000
0.020000 0.010000 0.060000 0.010000 0.010000 0.020000 0.010000 0.020000 0.010000
Not matched SDF shifts :
15.00 17.00 17.20 22.10 27.90 30.70 31.80 32.70 35.80 38.20 47.00 49.70 55.60 57.20 69.50 110.30

Rank: 31
ID : 847
Name : LTS0031032
CAS : nan
MW : 786.69
Score : 0.59 (20/34 carbons)
Cumulated absolute difference : 7.69
Spectrum shifts left : 27/47

Matched spectrum shifts :
62.64 65.79 69.21 70.63 71.26 71.32 71.71 72.01 75.71 76.25 76.75 77.22 78.19 78.61 93.67 98.34 99.50 103.43 105.47 134.36
Matched SDF shifts :
62.80 67.00 68.50 70.70 70.70 71.40 71.60 72.00 75.20 76.50 76.70 77.40 78.20 79.30 93.70 98.90 100.80 104.00 105.40
133.80
Matched spectrum intensities :
0.050000 0.040000 0.050000 0.050000 0.010000 0.050000 0.030000 0.020000 0.010000 0.030000 0.020000 0.010000 0.060000 0.010000
0.020000 0.030000 0.050000 0.020000 0.010000 0.010000
Not matched SDF shifts :
18.20 55.70 101.10 113.70 115.50 120.60 122.30 146.10 149.10 156.30 156.60 161.60 164.20 177.30

Rank: 32
ID : 658
Name : LTS0208698
CAS : nan
MW : 802.64

Score : 0.58 (19/33 carbons)
Cumulated absolute difference : 5.65
Spectrum shifts left : 28/47

Matched spectrum shifts :
61.91 62.64 71.26 71.32 71.71 73.80 74.30 74.51 75.71 76.75 77.11 77.22 78.19 78.61 93.67 99.50 103.43 105.47 134.36
Matched SDF shifts :
62.60 62.60 71.10 71.60 71.80 74.00 74.50 74.50 75.80 77.10 77.10 77.50 77.70 78.80 94.20 99.20 104.50 105.60 134.90
Matched spectrum intensities :
0.010000 0.050000 0.010000 0.050000 0.030000 0.020000 0.020000 0.020000 0.010000 0.020000 0.010000 0.010000 0.060000 0.010000 0.020000 0.050000 0.020000 0.010000 0.010000
Not matched SDF shifts :
100.40 102.20 115.50 115.60 121.30 121.60 145.20 148.80 156.00 159.10 160.80 163.00 171.90 179.50

Rank: 33
ID : 330
Name : LTS0081415
CAS : nan
MW : 772.66
Score : 0.58 (19/33 carbons)
Cumulated absolute difference : 6.61
Spectrum shifts left : 28/47

Matched spectrum shifts :
61.91 62.64 63.49 71.26 71.32 71.71 73.80 74.51 76.75 77.11 77.22 78.19 78.46 78.61 83.32 93.67 99.50 103.43 105.47
Matched SDF shifts :
62.50 62.60 62.60 71.20 71.10 71.60 73.90 74.50 77.10 77.80 77.50 77.90 78.80 78.70 82.80 94.40 99.10 102.60 105.40
Matched spectrum intensities :
0.010000 0.050000 0.010000 0.010000 0.050000 0.030000 0.020000 0.020000 0.020000 0.010000 0.010000 0.060000 0.010000 0.010000 0.020000 0.020000 0.050000 0.020000 0.010000
Not matched SDF shifts :
100.40 104.50 115.20 115.20 121.10 130.90 130.90 135.80 155.90 160.10 160.80 162.00 162.60 180.10

Rank: 34

ID : 645
Name : LTS0074468
CAS : nan
MW : 756.66
Score : 0.58 (19/33 carbons)
Cumulated absolute difference : 8.89
Spectrum shifts left : 28/47

Matched spectrum shifts :
61.91 62.64 69.21 70.63 71.26 71.71 72.01 74.51 76.25 76.75 77.11 77.22 78.61 83.32 93.67 98.34 99.50 103.43 105.47
Matched SDF shifts :
61.10 62.70 68.50 70.70 70.70 72.00 72.40 75.10 76.30 77.00 77.20 77.70 78.80 82.20 93.70 98.90 100.40 104.40 104.70
Matched spectrum intensities :
0.010000 0.050000 0.050000 0.050000 0.010000 0.030000 0.020000 0.020000 0.030000 0.020000 0.010000 0.010000 0.010000 0.020000 0.020000 0.030000 0.050000 0.020000 0.010000
Not matched SDF shifts :
18.20 101.80 115.20 115.20 121.10 130.90 130.90 132.70 156.50 156.80 160.10 161.00 164.20 177.30

Rank: 35
ID : 529
Name : LTS0257423
CAS : nan
MW : 948.83
Score : 0.57 (23/40 carbons)
Cumulated absolute difference : 8.49
Spectrum shifts left : 24/47

Matched spectrum shifts :
62.64 65.79 69.21 70.63 71.26 71.32 71.71 72.01 74.51 75.71 76.25 76.75 77.11 77.22 78.19 78.46 78.61 93.67 98.34 99.50 103.43 105.47 134.36
Matched SDF shifts :
62.80 67.00 68.50 70.70 71.60 71.40 71.80 72.00 75.20 75.20 76.50 76.70 76.90 77.40 78.20 78.60 79.30 93.70 98.90 100.80 104.00 105.40 133.80
Matched spectrum intensities :

0.050000 0.040000 0.050000 0.050000 0.010000 0.050000 0.030000 0.020000 0.020000 0.010000 0.030000 0.020000 0.010000 0.010000
0.060000 0.010000 0.010000 0.020000 0.030000 0.050000 0.020000 0.010000 0.010000
Not matched SDF shifts :
18.20 55.70 67.00 70.70 101.10 105.00 113.70 115.50 120.60 122.30 146.10 149.10 156.30 156.60 161.60 164.20 177.30

Rank: 36
ID : 150
Name : LTS0129228
CAS : nan
MW : 1097.2
Score : 0.57 (29/51 carbons)
Cumulated absolute difference : 11.32
Spectrum shifts left : 18/47

Matched spectrum shifts :
40.69 40.90 41.33 41.55 61.91 62.64 63.49 63.59 65.79 69.21 70.63 71.26 72.01 73.08 73.80 74.30 74.51 75.71 76.25 76.75
77.11 77.22 78.19 78.46 78.61 84.35 99.50 103.43 105.47
Matched SDF shifts :
40.80 41.00 41.80 41.80 61.90 62.60 63.10 62.60 66.90 69.30 70.60 70.00 72.00 73.10 74.10 74.30 74.50 75.10 77.10 77.10
77.50 77.50 78.30 78.40 79.40 85.10 100.60 104.00 105.20
Matched spectrum intensities :
0.880000 1.000000 0.440000 0.140000 0.010000 0.050000 0.010000 0.010000 0.040000 0.050000 0.050000 0.010000 0.020000 0.010000
0.020000 0.020000 0.020000 0.010000 0.030000 0.020000 0.010000 0.010000 0.060000 0.010000 0.010000 0.010000 0.050000 0.020000
0.010000
Not matched SDF shifts :
14.80 17.00 17.20 17.20 22.10 29.10 30.40 30.70 31.60 32.70 35.80 38.20 47.00 49.70 55.60 57.20 61.50 69.50 69.50 81.30
102.80 109.20

Rank: 37
ID : 796
Name : LTS0080305
CAS : nan
MW : 909.02
Score : 0.57 (26/46 carbons)
Cumulated absolute difference : 12.52

Spectrum shifts left : 21/47

Matched spectrum shifts :
40.05 40.26 40.69 40.90 41.33 41.55 61.91 62.64 63.49 69.21 71.71 72.01 73.08 73.41 73.80 74.30 74.51 75.71 77.22 78.19 78.46 78.61 103.43 105.47 127.50 134.36
Matched SDF shifts :
40.00 39.80 40.70 40.90 42.20 41.80 62.70 62.70 63.10 69.70 71.70 72.40 72.70 72.70 74.00 75.20 75.10 75.40 78.30 78.30 78.40 78.40 104.40 104.40 128.70 133.40
Matched spectrum intensities :
0.010000 0.150000 0.880000 1.000000 0.440000 0.140000 0.010000 0.050000 0.010000 0.050000 0.030000 0.020000 0.010000 0.020000 0.020000 0.020000 0.020000 0.010000 0.010000 0.060000 0.010000 0.010000 0.020000 0.010000 0.010000 0.010000
Not matched SDF shifts :
14.90 17.00 17.10 18.20 21.40 32.70 34.70 38.50 38.80 39.10 42.50 56.30 78.90 81.30 110.60 128.70 129.80 129.80 129.80 166.10

Rank: 38
ID : 745
Name : LTS0261262
CAS : nan
MW : 788.92
Score : 0.56 (22/39 carbons)
Cumulated absolute difference : 6.78
Spectrum shifts left : 25/47

Matched spectrum shifts :
40.05 40.69 40.90 41.33 41.55 61.91 62.64 63.49 64.35 64.41 69.21 70.63 71.26 73.80 74.51 75.71 76.25 76.75 77.11 78.61 84.35 105.47
Matched SDF shifts :
39.30 40.80 41.00 42.60 41.80 61.30 62.70 63.10 64.40 64.70 69.30 70.60 70.60 73.70 74.50 75.10 76.30 77.00 77.20 78.70 85.10 105.30
Matched spectrum intensities :
0.010000 0.880000 1.000000 0.440000 0.140000 0.010000 0.050000 0.010000 0.020000 0.020000 0.050000 0.050000 0.010000 0.020000 0.020000 0.010000 0.030000 0.020000 0.010000 0.010000 0.010000 0.010000
Not matched SDF shifts :
14.80 17.00 17.20 22.10 27.90 30.70 31.80 32.70 34.70 38.20 47.00 49.80 55.60 57.20 81.30 105.80 110.30

Rank: 39
ID : 250
Name : LTS0235000
CAS : nan
MW : 550.59
Score : 0.56 (14/25 carbons)
Cumulated absolute difference : 5.46
Spectrum shifts left : 33/47

Matched spectrum shifts :
62.64 63.49 64.35 73.41 74.30 74.51 75.71 76.25 77.11 77.22 78.19 78.46 103.43 105.47
Matched SDF shifts :
62.60 62.80 64.40 73.60 74.20 75.20 75.70 75.20 77.00 77.90 78.00 78.20 103.30 104.20
Matched spectrum intensities :
0.050000 0.010000 0.020000 0.020000 0.020000 0.020000 0.010000 0.030000 0.010000 0.010000 0.060000 0.010000 0.020000 0.010000
Not matched SDF shifts :
21.10 26.20 28.10 28.10 34.90 36.10 47.00 49.10 123.60 147.80 202.60

Rank: 40
ID : 837
Name : LTS0225418
CAS : nan
MW : 610.52
Score : 0.56 (15/27 carbons)
Cumulated absolute difference : 5.66
Spectrum shifts left : 32/47

Matched spectrum shifts :
61.91 62.64 71.26 71.71 74.51 76.75 77.11 77.22 78.19 78.61 93.67 98.34 99.50 103.43 105.47
Matched SDF shifts :
62.60 62.60 71.10 71.60 74.50 77.10 77.10 77.50 77.70 78.80 93.70 98.90 100.40 104.50 104.70
Matched spectrum intensities :

0.010000 0.050000 0.010000 0.030000 0.020000 0.020000 0.010000 0.010000 0.060000 0.010000 0.020000 0.030000 0.050000 0.020000 0.010000
Not matched SDF shifts :
115.20 115.20 121.10 130.90 130.90 132.70 156.50 156.80 160.10 161.60 164.20 177.30

Rank: 41
ID : 668
Name : LTS0161746
CAS : nan
MW : 948.83
Score : 0.55 (22/40 carbons)
Cumulated absolute difference : 7.73
Spectrum shifts left : 25/47

Matched spectrum shifts :
62.64 69.21 71.26 71.32 71.71 72.01 73.08 73.41 73.80 74.30 76.25 76.75 77.11 77.22 78.19 78.46 78.61 93.67 98.34 99.50 103.43 134.36
Matched SDF shifts :
62.80 69.80 71.70 71.40 71.80 72.00 72.70 72.60 73.90 73.90 76.50 76.70 77.00 77.30 78.00 78.30 77.40 93.70 98.90 100.80 103.60 133.80
Matched spectrum intensities :
0.050000 0.050000 0.010000 0.050000 0.030000 0.020000 0.010000 0.020000 0.020000 0.020000 0.030000 0.020000 0.010000 0.010000 0.060000 0.010000 0.010000 0.020000 0.030000 0.050000 0.020000 0.010000
Not matched SDF shifts :
18.50 55.70 67.30 68.50 101.10 102.80 103.80 115.10 115.50 123.00 124.90 146.10 149.10 156.50 156.60 161.00 164.20 177.30

Rank: 42
ID : 388
Name : LTS0210976
CAS : nan
MW : 1097.2
Score : 0.55 (28/51 carbons)
Cumulated absolute difference : 8.97
Spectrum shifts left : 19/47

Matched spectrum shifts :
40.69 40.90 41.33 41.55 61.91 62.64 63.49 63.59 65.79 69.21 70.63 71.26 71.32 73.08 73.80 74.30 74.51 75.71 76.25 76.75
77.11 77.22 78.19 78.46 78.61 84.35 103.43 105.47
Matched SDF shifts :
40.80 41.00 41.80 41.80 61.90 62.70 63.10 62.70 66.90 69.30 70.60 70.60 70.60 73.10 73.70 74.50 75.10 75.60 76.30 77.00
77.20 77.50 78.40 79.40 78.80 85.10 103.40 105.20
Matched spectrum intensities :
0.880000 1.000000 0.440000 0.140000 0.010000 0.050000 0.010000 0.010000 0.040000 0.050000 0.050000 0.010000 0.050000 0.010000
0.020000 0.020000 0.020000 0.010000 0.030000 0.020000 0.010000 0.010000 0.060000 0.010000 0.010000 0.010000 0.020000 0.010000
Not matched SDF shifts :
14.80 17.00 17.20 17.20 22.10 29.10 30.40 30.70 31.60 32.70 34.70 38.20 47.00 49.80 55.60 57.20 61.30 69.80 81.00 81.30
104.40 104.40 109.20

Rank: 43
ID : 559
Name : LTS0138561
CAS : nan
MW : 1081.2
Score : 0.55 (28/51 carbons)
Cumulated absolute difference : 10.98
Spectrum shifts left : 19/47

Matched spectrum shifts :
40.05 40.69 41.55 61.91 62.64 63.49 63.59 65.79 69.21 70.63 71.26 72.01 73.08 73.80 74.30 74.51 75.71 76.25 76.75 77.11
77.22 78.19 78.46 78.61 84.35 99.50 103.43 105.47
Matched SDF shifts :
40.10 40.60 41.80 61.90 62.60 63.10 62.60 66.90 69.50 70.60 70.00 72.00 73.10 74.10 74.30 74.50 75.10 77.10 77.10 77.50
77.50 78.30 78.40 79.40 85.10 100.60 104.00 105.20

Matched spectrum intensities :
0.010000 0.880000 0.140000 0.010000 0.050000 0.010000 0.010000 0.040000 0.050000 0.050000 0.010000 0.020000 0.010000 0.020000
0.020000 0.020000 0.010000 0.030000 0.020000 0.010000 0.010000 0.060000 0.010000 0.010000 0.010000 0.050000 0.020000 0.010000
Not matched SDF shifts :

14.80 15.60 16.60 17.20 21.50 28.10 29.10 30.40 30.40 31.60 32.20 33.50 35.40 36.90 44.60 45.90 54.40 56.40 61.50 69.50 81.30 102.80 109.20

Rank: 44
ID : 876
Name : LTS0246349
CAS : nan
MW : 756.66
Score : 0.55 (18/33 carbons)
Cumulated absolute difference : 5.63
Spectrum shifts left : 29/47

Matched spectrum shifts :
61.91 62.64 70.63 71.26 71.71 72.01 74.30 75.71 77.11 77.22 78.19 78.46 78.61 93.67 98.34 99.50 103.43 105.47
Matched SDF shifts :
62.60 62.70 70.00 71.30 71.70 72.40 74.20 75.90 77.00 77.70 78.10 78.40 78.80 93.70 98.90 99.10 104.70 105.80
Matched spectrum intensities :
0.010000 0.050000 0.050000 0.010000 0.030000 0.020000 0.020000 0.010000 0.010000 0.010000 0.060000 0.010000 0.010000 0.020000 0.030000 0.050000 0.020000 0.010000
Not matched SDF shifts :
17.20 87.50 100.40 115.20 115.20 121.10 130.90 130.90 132.70 156.50 156.80 160.10 161.00 164.20 177.30

Rank: 45
ID : 547
Name : LTS0142113
CAS : nan
MW : 756.66
Score : 0.55 (18/33 carbons)
Cumulated absolute difference : 5.67
Spectrum shifts left : 29/47

Matched spectrum shifts :
61.91 62.64 70.63 71.26 71.32 71.71 74.51 75.71 77.11 77.22 78.19 78.46 78.61 93.67 98.34 99.50 103.43 105.47
Matched SDF shifts :

62.60 62.60 70.00 71.10 71.60 71.70 75.10 75.90 77.10 77.70 78.30 78.40 78.80 93.70 98.90 99.10 103.90 104.70
Matched spectrum intensities :
0.010000 0.050000 0.050000 0.010000 0.050000 0.030000 0.020000 0.010000 0.010000 0.010000 0.060000 0.010000 0.010000 0.020000 0.030000 0.050000 0.020000 0.010000
Not matched SDF shifts :
17.20 87.50 100.40 115.20 115.20 121.10 130.90 130.90 132.70 156.50 156.80 160.10 161.60 164.20 177.30

Rank: 46
ID : 217
Name : LTS0202010
CAS : nan
MW : 642.78
Score : 0.55 (18/33 carbons)
Cumulated absolute difference : 6.60
Spectrum shifts left : 29/47

Matched spectrum shifts :
40.05 40.26 40.69 40.90 41.33 41.55 62.64 63.49 69.21 70.63 71.26 73.80 74.51 75.71 78.19 78.46 78.61 103.43
Matched SDF shifts :
40.00 39.10 40.70 40.90 42.20 41.80 62.70 63.10 69.70 70.60 70.70 74.00 75.10 75.20 78.30 78.40 78.90 104.40
Matched spectrum intensities :
0.010000 0.150000 0.880000 1.000000 0.440000 0.140000 0.050000 0.010000 0.050000 0.050000 0.010000 0.020000 0.020000 0.010000 0.060000 0.010000 0.010000 0.020000
Not matched SDF shifts :
14.90 17.00 17.10 18.20 21.40 30.70 32.70 34.70 37.60 38.50 38.80 56.30 69.80 81.30 110.60

Rank: 47
ID : 710
Name : LTS0184521
CAS : nan
MW : 756.66
Score : 0.55 (18/33 carbons)
Cumulated absolute difference : 6.86

Spectrum shifts left : 29/47

Matched spectrum shifts :
61.91 62.64 69.21 70.63 71.32 71.71 74.51 75.71 77.11 77.22 78.19 78.46 78.61 93.67 98.34 99.50 103.43 105.47
Matched SDF shifts :
61.10 62.60 69.90 70.00 71.50 71.70 75.10 75.90 77.00 77.70 78.10 78.40 78.80 93.70 98.90 99.10 102.40 104.70
Matched spectrum intensities :
0.010000 0.050000 0.050000 0.050000 0.050000 0.030000 0.020000 0.010000 0.010000 0.010000 0.060000 0.010000 0.010000 0.020000 0.030000 0.050000 0.020000 0.010000
Not matched SDF shifts :
17.20 87.50 100.40 115.20 115.20 121.10 130.90 130.90 132.70 156.50 156.80 160.10 161.00 164.20 177.30

Rank: 48
ID : 343
Name : LTS0105990
CAS : nan
MW : 756.66
Score : 0.55 (18/33 carbons)
Cumulated absolute difference : 7.80
Spectrum shifts left : 29/47

Matched spectrum shifts :
61.91 62.64 69.21 70.63 71.32 71.71 74.51 75.71 77.11 77.22 78.19 78.46 78.61 93.67 98.34 99.50 103.43 105.47
Matched SDF shifts :
61.10 62.60 69.90 70.00 71.50 71.70 75.10 75.90 78.10 77.70 78.30 78.40 78.80 93.70 98.90 99.10 104.50 104.70
Matched spectrum intensities :
0.010000 0.050000 0.050000 0.050000 0.050000 0.030000 0.020000 0.010000 0.010000 0.010000 0.060000 0.010000 0.010000 0.020000 0.030000 0.050000 0.020000 0.010000
Not matched SDF shifts :
17.20 87.50 100.40 115.20 115.20 121.10 130.90 130.90 132.70 156.50 156.80 160.10 161.00 164.20 177.30

Rank: 49

ID : 942
Name : LTS0126921
CAS : nan
MW : 1101.19
Score : 0.54 (27/50 carbons)
Cumulated absolute difference : 7.63
Spectrum shifts left : 20/47

Matched spectrum shifts :
40.05 40.48 40.69 41.12 61.91 62.64 63.59 64.35 69.21 70.63 71.71 72.01 73.80 74.30 74.51 75.71 76.25 77.11 77.22 78.19 78.46 78.61 83.99 84.35 99.50 103.43 105.47
Matched SDF shifts :
39.70 40.50 40.80 41.10 61.50 62.80 63.80 64.20 69.30 70.60 72.00 72.00 74.20 74.50 74.50 75.20 75.00 77.10 77.10 78.10 78.20 79.20 83.70 85.10 99.20 102.80 105.10
Matched spectrum intensities :
0.010000 0.440000 0.880000 0.870000 0.010000 0.050000 0.010000 0.020000 0.050000 0.050000 0.030000 0.020000 0.020000 0.020000 0.020000 0.010000 0.030000 0.010000 0.010000 0.060000 0.010000 0.010000 0.010000 0.010000 0.050000 0.020000 0.010000
Not matched SDF shifts :
16.30 16.50 17.20 17.60 22.10 28.20 30.70 30.90 32.10 33.90 35.80 38.20 47.00 49.70 55.60 56.30 61.50 79.40 79.40 81.20 105.80 110.60 112.60

Rank: 50
ID : 834
Name : LTS0008293
CAS : nan
MW : 1083.17
Score : 0.54 (27/50 carbons)
Cumulated absolute difference : 8.22
Spectrum shifts left : 20/47

Matched spectrum shifts :
40.05 40.26 40.90 41.33 41.55 61.91 62.64 63.49 65.79 69.21 70.63 71.26 71.32 73.41 73.80 74.30 74.51 75.71 76.25 76.75 77.11 77.22 78.19 78.46 78.61 103.43 105.47
Matched SDF shifts :

40.00 39.10 40.90 41.80 41.80 61.30 62.70 63.10 66.80 69.90 70.60 71.30 71.40 73.70 73.80 74.50 74.50 75.50 76.40 77.20
77.20 78.00 78.00 78.40 78.70 103.40 106.30
Matched spectrum intensities :
0.010000 0.150000 1.000000 0.440000 0.140000 0.010000 0.050000 0.010000 0.040000 0.050000 0.050000 0.010000 0.050000 0.020000
0.020000 0.020000 0.020000 0.010000 0.030000 0.020000 0.010000 0.010000 0.060000 0.010000 0.010000 0.020000 0.010000
Not matched SDF shifts :
13.50 14.80 17.00 17.20 21.40 29.10 30.40 30.70 31.60 32.70 34.70 37.60 38.50 56.30 61.30 66.90 75.20 78.80 79.40 81.30
104.40 106.40 109.20

::

A. Results of MixONat from Amaryllidaceae DB2

Allowed margin : 1.3
No molecular weight information
Number of results : 50
Equivalent carbons not allowed

Rank: 1
ID : 946
Name : LTS0155285
CAS : nan
MW : 92.09
Score : 1.0 (3/3 carbons)
Cumulated absolute difference : 1.04
Spectrum shifts left : 44/47

Matched spectrum shifts :
64.41   65.15   73.80
Matched SDF shifts :
64.90   64.90   74.10
Matched spectrum intensities :
0.020000   0.010000   0.020000

Rank: 2
ID : 902
Name : LTS0272557
CAS : nan
MW : 342.3
Score : 1.0 (12/12 carbons)
Cumulated absolute difference : 2.12
Spectrum shifts left : 35/47

Matched spectrum shifts :
61.91 63.59 64.35 70.63 72.01 73.41 74.30 75.71 78.19 83.32 93.67 105.47
Matched SDF shifts :
61.80 63.70 64.30 70.20 72.00 73.60 74.40 75.80 78.20 83.10 92.90 105.50
Matched spectrum intensities :
0.010000 0.010000 0.020000 0.050000 0.020000 0.020000 0.020000 0.010000 0.060000 0.020000 0.020000 0.010000

Rank: 3
ID : 1677
Name : LTS0013597
CAS : nan
MW : 180.16
Score : 1.0 (6/6 carbons)
Cumulated absolute difference : 2.20
Spectrum shifts left : 41/47

Matched spectrum shifts :
61.91 71.26 72.01 73.08 73.41 93.67
Matched SDF shifts :
61.60 71.10 72.40 72.80 73.50 92.70
Matched spectrum intensities :
0.010000 0.010000 0.020000 0.010000 0.020000 0.020000

Rank: 4
ID : 954
Name : LTS0199986
CAS : nan
MW : 182.17
Score : 1.0 (6/6 carbons)
Cumulated absolute difference : 2.78
Spectrum shifts left : 41/47

Matched spectrum shifts :
65.15 65.79 73.08 73.41 74.30 74.51
Matched SDF shifts :

66.30   66.30   73.40   73.40   74.80   74.80
Matched spectrum intensities :
0.010000   0.040000   0.010000   0.020000   0.020000   0.020000

Rank: 5
ID : 845
Name : LTS0150163
CAS : nan
MW : 122.12
Score : 1.0 (4/4 carbons)
Cumulated absolute difference : 2.82
Spectrum shifts left : 43/47

Matched spectrum shifts :
65.15   65.79   75.71   76.25
Matched SDF shifts :
66.40   66.40   75.50   75.50
Matched spectrum intensities :
0.010000   0.040000   0.010000   0.030000

Rank: 6
ID : 1866
Name : LTS0113066
CAS : nan
MW : 504.44
Score : 1.0 (18/18 carbons)
Cumulated absolute difference : 4.29
Spectrum shifts left : 29/47

Matched spectrum shifts :
62.64   63.59   64.35   69.21   71.71   72.01   73.41   73.80   74.30   74.51   75.71   76.25   77.22   78.19   83.32   93.67   103.43   105.47
Matched SDF shifts :
62.80   63.70   64.30   68.60   71.70   72.10   73.70   73.90   74.10   75.40   75.80   76.30   77.30   78.20   83.10   94.80   103.60   105.50

Matched spectrum intensities :
0.050000 0.010000 0.020000 0.050000 0.030000 0.020000 0.020000 0.020000 0.020000 0.020000 0.010000 0.030000 0.010000 0.060000 0.020000 0.020000 0.020000 0.010000

Rank: 7
ID : 1481
Name : LTS0162289
CAS : nan
MW : 324.28
Score : 0.92 (11/12 carbons)
Cumulated absolute difference : 4.53
Spectrum shifts left : 36/47

Matched spectrum shifts :
62.64 63.49 63.59 65.79 70.63 74.30 75.71 78.46 83.32 83.99 103.43
Matched SDF shifts :
62.80 63.10 62.80 66.30 70.00 74.40 75.60 78.50 83.70 83.70 102.30
Matched spectrum intensities :
0.050000 0.010000 0.010000 0.040000 0.050000 0.020000 0.010000 0.010000 0.020000 0.010000 0.020000
Not matched SDF shifts :
102.30

Rank: 8
ID : 837
Name : LTS0146582
CAS : nan
MW : 318.3
Score : 0.89 (8/9 carbons)
Cumulated absolute difference : 3.66
Spectrum shifts left : 39/47

Matched spectrum shifts :
40.05 63.49 69.21 71.26 72.01 73.41 73.80 105.47
Matched SDF shifts :
39.10 63.30 68.50 71.10 72.40 73.60 73.70 104.50

Matched spectrum intensities :
0.010000 0.010000 0.050000 0.010000 0.020000 0.020000 0.020000 0.010000
Not matched SDF shifts :
80.60

Rank: 9
ID : 1191
Name : LTS0183884
CAS : nan
MW : 666.58
Score : 0.88 (21/24 carbons)
Cumulated absolute difference : 4.74
Spectrum shifts left : 26/47

Matched spectrum shifts :
62.64 63.59 64.35 69.21 71.32 71.71 72.01 73.41 73.80 74.30 74.51 75.71 76.25 77.11 77.22 78.19 78.46 83.32 93.67 103.43 105.47
Matched SDF shifts :
62.80 63.70 64.30 68.60 71.70 71.80 72.10 73.70 73.90 74.10 73.90 75.80 76.30 77.00 77.30 78.20 78.30 83.10 94.80 103.60 105.50
Matched spectrum intensities :
0.050000 0.010000 0.020000 0.050000 0.050000 0.030000 0.020000 0.020000 0.020000 0.020000 0.020000 0.010000 0.030000 0.010000 0.010000 0.060000 0.010000 0.020000 0.020000 0.020000 0.010000
Not matched SDF shifts :
67.30 75.40 103.80

Rank: 10
ID : 1738
Name : LTS0062089
CAS : nan
MW : 666.58
Score : 0.88 (21/24 carbons)
Cumulated absolute difference : 4.74
Spectrum shifts left : 26/47

Matched spectrum shifts :
62.64 63.59 64.35 69.21 71.32 71.71 72.01 73.41 73.80 74.30 74.51 75.71 76.25 77.11 77.22 78.19 78.46 83.32 93.67 103.43 105.47
Matched SDF shifts :
62.80 63.70 64.30 68.60 71.70 71.80 72.10 73.70 73.90 74.10 73.90 75.80 76.30 77.00 77.30 78.20 78.30 83.10 94.80 103.60 105.50
Matched spectrum intensities :
0.050000 0.010000 0.020000 0.050000 0.050000 0.030000 0.020000 0.020000 0.020000 0.020000 0.020000 0.010000 0.030000 0.010000 0.010000 0.060000 0.010000 0.020000 0.020000 0.020000 0.010000
Not matched SDF shifts :
67.30 75.40 103.80

Rank: 11
ID : 1461
Name : LTS0241114
CAS : nan
MW : 180.16
Score : 0.83 (5/6 carbons)
Cumulated absolute difference : 0.80
Spectrum shifts left : 42/47

Matched spectrum shifts :
64.35 65.79 73.08 75.71 76.25
Matched SDF shifts :
64.40 66.20 73.00 75.50 76.20
Matched spectrum intensities :
0.020000 0.040000 0.010000 0.010000 0.030000
Not matched SDF shifts :
217.40

Rank: 12
ID : 1228
Name : LTS0276202
CAS : nan
MW : 180.16

Score : 0.83 (5/6 carbons)
Cumulated absolute difference : 0.88
Spectrum shifts left : 42/47

Matched spectrum shifts :
65.79 73.08 73.41 74.51 76.25
Matched SDF shifts :
66.30 73.10 73.40 74.80 76.20
Matched spectrum intensities :
0.040000 0.010000 0.020000 0.020000 0.030000
Not matched SDF shifts :
200.80

Rank: 13
ID : 1478
Name : LTS0241274
CAS : nan
MW : 180.16
Score : 0.83 (5/6 carbons)
Cumulated absolute difference : 1.18
Spectrum shifts left : 42/47

Matched spectrum shifts :
65.79 71.32 72.01 73.41 74.51
Matched SDF shifts :
66.30 71.50 72.20 73.40 74.80
Matched spectrum intensities :
0.040000 0.050000 0.020000 0.020000 0.020000
Not matched SDF shifts :
201.30

Rank: 14
ID : 1678
Name : LTS0128031
CAS : nan

MW : 180.16
Score : 0.83 (5/6 carbons)
Cumulated absolute difference : 1.18
Spectrum shifts left : 42/47

Matched spectrum shifts :
65.79 70.63 73.41 73.80 74.51
Matched SDF shifts :
66.30 70.70 73.40 73.50 74.80
Matched spectrum intensities :
0.040000 0.050000 0.020000 0.020000 0.020000
Not matched SDF shifts :
200.80

Rank: 15
ID : 877
Name : LTS0262158
CAS : nan
MW : 180.16
Score : 0.83 (5/6 carbons)
Cumulated absolute difference : 1.39
Spectrum shifts left : 42/47

Matched spectrum shifts :
65.79 73.08 73.41 73.80 74.51
Matched SDF shifts :
66.30 72.80 73.40 73.50 74.80
Matched spectrum intensities :
0.040000 0.010000 0.020000 0.020000 0.020000
Not matched SDF shifts :
200.80

Rank: 16
ID : 1976
Name : LTS0259277
CAS : nan

MW : 180.16
Score : 0.83 (5/6 carbons)
Cumulated absolute difference : 1.58
Spectrum shifts left : 42/47

Matched spectrum shifts :
63.49 63.59 69.21 70.63 98.34
Matched SDF shifts :
63.30 63.80 69.20 69.70 98.10
Matched spectrum intensities :
0.010000 0.010000 0.050000 0.050000 0.030000
Not matched SDF shifts :
67.50

Rank: 17
ID : 1223
Name : LTS0271461
CAS : nan
MW : 194.14
Score : 0.83 (5/6 carbons)
Cumulated absolute difference : 2.62
Spectrum shifts left : 42/47

Matched spectrum shifts :
72.01 73.08 73.41 75.71 93.67
Matched SDF shifts :
72.30 73.00 72.80 75.30 94.90
Matched spectrum intensities :
0.020000 0.010000 0.020000 0.010000 0.020000
Not matched SDF shifts :
172.90

Rank: 18
ID : 517
Name : LTS0192374

CAS : nan
MW : 324.28
Score : 0.83 (10/12 carbons)
Cumulated absolute difference : 2.64
Spectrum shifts left : 37/47

Matched spectrum shifts :
63.49 63.59 64.35 69.21 73.08 74.30 75.71 78.46 83.32 105.47
Matched SDF shifts :
63.50 63.90 63.90 68.00 73.20 74.40 75.60 78.50 83.30 105.20
Matched spectrum intensities :
0.010000 0.010000 0.020000 0.050000 0.010000 0.020000 0.010000 0.010000 0.020000 0.010000
Not matched SDF shifts :
82.60 105.20

Rank: 19
ID : 820
Name : LTS0047771
CAS : nan
MW : 180.16
Score : 0.83 (5/6 carbons)
Cumulated absolute difference : 3.87
Spectrum shifts left : 42/47

Matched spectrum shifts :
71.71 72.01 73.08 73.41 73.80
Matched SDF shifts :
72.70 72.70 72.70 72.70 72.70
Matched spectrum intensities :
0.030000 0.020000 0.010000 0.020000 0.020000
Not matched SDF shifts :
72.70

Rank: 20
ID : 747

Name : LTS0110247
CAS : nan
MW : 354.26
Score : 0.83 (10/12 carbons)
Cumulated absolute difference : 4.10
Spectrum shifts left : 37/47

Matched spectrum shifts :
61.91 63.49 65.79 71.26 73.08 74.30 75.71 76.25 76.75 98.34
Matched SDF shifts :
60.80 63.20 66.60 71.20 72.80 74.20 75.70 76.20 75.90 97.80
Matched spectrum intensities :
0.010000 0.010000 0.040000 0.010000 0.010000 0.020000 0.010000 0.030000 0.020000 0.030000
Not matched SDF shifts :
168.60 170.40

Rank: 21
ID : 678
Name : LTS0157972
CAS : nan
MW : 458.46
Score : 0.83 (15/18 carbons)
Cumulated absolute difference : 7.91
Spectrum shifts left : 32/47

Matched spectrum shifts :
62.64 63.49 65.15 65.79 69.21 71.26 71.32 71.71 72.01 74.51 75.71 78.19 78.46 103.43 105.47
Matched SDF shifts :
62.80 62.80 66.40 66.40 68.80 71.50 71.50 72.20 72.20 75.10 75.10 78.10 78.10 104.40 104.40
Matched spectrum intensities :
0.050000 0.010000 0.010000 0.040000 0.050000 0.010000 0.050000 0.030000 0.020000 0.020000 0.010000 0.060000 0.010000 0.020000 0.010000
Not matched SDF shifts :
30.20 30.20 68.80

Rank: 22
ID : 591
Name : LTS0085513
CAS : nan
MW : 150.13
Score : 0.8 (4/5 carbons)
Cumulated absolute difference : 0.27
Spectrum shifts left : 43/47

Matched spectrum shifts :
64.35 73.08 73.41 76.25
Matched SDF shifts :
64.40 73.00 73.50 76.20
Matched spectrum intensities :
0.020000 0.010000 0.020000 0.030000
Not matched SDF shifts :
200.80

Rank: 23
ID : 1249
Name : LTS0269907
CAS : nan
MW : 150.13
Score : 0.8 (4/5 carbons)
Cumulated absolute difference : 1.01
Spectrum shifts left : 43/47

Matched spectrum shifts :
64.35 72.01 73.08 73.41
Matched SDF shifts :
64.40 72.80 73.00 73.50
Matched spectrum intensities :
0.020000 0.020000 0.010000 0.020000
Not matched SDF shifts :
200.80

Rank: 24
ID : 1643
Name : LTS0110579
CAS : nan
MW : 504.44
Score : 0.78 (14/18 carbons)
Cumulated absolute difference : 3.05
Spectrum shifts left : 33/47

Matched spectrum shifts :
61.91 62.64 69.21 72.01 73.08 73.41 73.80 74.30 74.51 77.11 78.19 78.46 78.61 103.43
Matched SDF shifts :
61.50 62.60 69.50 72.00 73.40 74.10 74.00 74.30 74.50 77.10 78.30 78.60 79.40 103.40
Matched spectrum intensities :
0.010000 0.050000 0.050000 0.020000 0.010000 0.020000 0.020000 0.020000 0.020000 0.010000 0.060000 0.010000 0.010000 0.020000
Not matched SDF shifts :
61.10 79.40 95.30 103.80

Rank: 25
ID : 989
Name : LTS0143481
CAS : nan
MW : 280.27
Score : 0.73 (8/11 carbons)
Cumulated absolute difference : 1.94
Spectrum shifts left : 39/47

Matched spectrum shifts :
40.05 62.64 70.63 73.80 74.51 75.71 78.19 103.43
Matched SDF shifts :
39.30 62.60 70.60 73.80 75.20 75.70 77.90 103.30
Matched spectrum intensities :
0.010000 0.050000 0.050000 0.020000 0.020000 0.010000 0.060000 0.020000
Not matched SDF shifts :
20.80 51.90 171.30

Rank: 26
ID : 1871
Name : LTS0137131
CAS : nan
MW : 504.44
Score : 0.72 (13/18 carbons)
Cumulated absolute difference : 3.68
Spectrum shifts left : 34/47

Matched spectrum shifts :
61.91 71.32 73.08 73.41 73.80 74.30 74.51 76.25 76.75 77.11 78.46 78.61 103.43
Matched SDF shifts :
61.30 71.40 73.70 73.40 74.00 74.30 74.50 76.30 77.00 77.20 79.40 79.40 103.40
Matched spectrum intensities :
0.010000 0.050000 0.010000 0.020000 0.020000 0.020000 0.020000 0.030000 0.020000 0.010000 0.010000 0.010000 0.020000
Not matched SDF shifts :
61.10 61.10 74.10 95.30 103.80

Rank: 27
ID : 1581
Name : LTS0247235
CAS : nan
MW : 666.58
Score : 0.71 (17/24 carbons)
Cumulated absolute difference : 6.38
Spectrum shifts left : 30/47

Matched spectrum shifts :
61.91 62.64 63.49 63.59 64.35 64.41 64.48 74.30 74.51 75.71 76.25 78.46 78.61 83.32 83.99 84.35 105.47
Matched SDF shifts :
63.10 62.80 63.20 63.20 63.90 63.90 63.90 74.40 74.40 75.90 76.20 79.20 79.20 83.30 83.70 84.90 105.30
Matched spectrum intensities :
0.010000 0.050000 0.010000 0.010000 0.020000 0.020000 0.040000 0.020000 0.020000 0.010000 0.030000 0.010000 0.010000 0.020000
0.010000 0.010000 0.010000
Not matched SDF shifts :

63.10 80.70 80.70 84.90 105.30 107.90 107.90

Rank: 28
ID : 1216
Name : LTS0196709
CAS : nan
MW : 666.58
Score : 0.7 (16/23 carbons)
Cumulated absolute difference : 5.73
Spectrum shifts left : 31/47

Matched spectrum shifts :
61.91 69.21 73.08 73.41 73.80 74.30 74.51 75.71 76.25 76.75 77.11 77.22 78.19 78.46 78.61 103.43
Matched SDF shifts :
61.60 68.90 73.70 73.70 74.00 74.30 74.50 75.10 76.30 77.00 77.20 77.20 79.40 79.40 79.40 103.40
Matched spectrum intensities :
0.010000 0.050000 0.010000 0.020000 0.020000 0.020000 0.020000 0.010000 0.030000 0.020000 0.010000 0.010000 0.060000 0.010000 0.010000 0.020000
Not matched SDF shifts :
61.10 61.10 61.30 74.10 95.30 103.80 103.80

Rank: 29
ID : 1588
Name : LTS0204729
CAS : nan
MW : 122.21
Score : 0.67 (2/3 carbons)
Cumulated absolute difference : 0.26
Spectrum shifts left : 45/47

Matched spectrum shifts :
127.50 134.36
Matched SDF shifts :
127.50 134.10
Matched spectrum intensities :

0.010000   0.010000
Not matched SDF shifts :
16.50

Rank: 30
ID : 1574
Name : LTS0247365
CAS : nan
MW : 164.16
Score : 0.67 (4/6 carbons)
Cumulated absolute difference : 1.29
Spectrum shifts left : 43/47

Matched spectrum shifts :
69.21   73.08   73.41   74.51
Matched SDF shifts :
69.00   72.80   73.10   75.00
Matched spectrum intensities :
0.050000   0.010000   0.020000   0.020000
Not matched SDF shifts :
18.90   200.80

Rank: 31
ID : 1146
Name : LTS0262385
CAS : nan
MW : 154.21
Score : 0.67 (2/3 carbons)
Cumulated absolute difference : 1.39
Spectrum shifts left : 45/47

Matched spectrum shifts :
118.03   134.36
Matched SDF shifts :
117.80   133.20

Matched spectrum intensities :
0.010000 0.010000
Not matched SDF shifts :
59.00

Rank: 32
ID : 1431
Name : LTS0216911
CAS : nan
MW : 666.58
Score : 0.67 (16/24 carbons)
Cumulated absolute difference : 7.46
Spectrum shifts left : 31/47

Matched spectrum shifts :
61.91 62.64 63.49 63.59 64.35 74.30 74.51 75.71 76.25 78.19 78.46 78.61 83.32 83.99 84.35 105.47
Matched SDF shifts :
62.80 62.80 63.20 63.20 63.10 74.40 74.40 76.20 76.20 79.20 79.20 79.20 83.70 83.70 84.90 105.30
Matched spectrum intensities :
0.010000 0.050000 0.010000 0.010000 0.020000 0.020000 0.020000 0.010000 0.030000 0.060000 0.010000 0.010000 0.020000 0.010000
0.010000 0.010000
Not matched SDF shifts :
63.10 63.10 63.10 79.20 84.90 105.30 105.30 105.30

Rank: 33
ID : 968
Name : LTS0145917
CAS : nan
MW : 280.27
Score : 0.64 (7/11 carbons)
Cumulated absolute difference : 2.86
Spectrum shifts left : 40/47

Matched spectrum shifts :
40.05 61.91 71.71 74.51 76.25 77.22 103.43

Matched SDF shifts :
40.10 61.10 71.70 75.10 76.30 77.70 104.30
Matched spectrum intensities :
0.010000 0.010000 0.030000 0.020000 0.030000 0.010000 0.020000
Not matched SDF shifts :
21.10 51.90 67.20 170.80

Rank: 34
ID : 1214
Name : LTS0188265
CAS : nan
MW : 788.66
Score : 0.61 (20/33 carbons)
Cumulated absolute difference : 8.47
Spectrum shifts left : 27/47

Matched spectrum shifts :
61.91 62.64 63.49 71.26 71.32 71.71 73.80 74.51 76.75 77.11 77.22 78.19 78.46 78.61 83.32 93.67 99.50 103.43 105.47 134.36
Matched SDF shifts :
62.50 62.60 62.60 71.20 71.10 71.60 73.90 74.50 77.10 77.80 77.50 77.90 78.80 78.70 82.80 94.70 99.10 104.50 105.60 133.10
Matched spectrum intensities :
0.010000 0.050000 0.010000 0.010000 0.050000 0.030000 0.020000 0.020000 0.020000 0.010000 0.010000 0.060000 0.010000 0.010000 0.020000 0.020000 0.050000 0.020000 0.010000 0.010000
Not matched SDF shifts :
100.40 101.10 115.50 115.60 121.30 121.60 145.20 148.80 156.00 156.50 160.80 162.60 177.60

Rank: 35
ID : 1645
Name : LTS0015975
CAS : nan
MW : 487.41
Score : 0.6 (12/20 carbons)
Cumulated absolute difference : 3.93
Spectrum shifts left : 35/47

Matched spectrum shifts :

41.33 62.64 65.79 69.21 71.71 72.01 73.80 75.71 77.22 78.19 78.61 103.43
Matched SDF shifts :
41.30 62.60 66.50 68.10 71.60 72.30 74.00 75.50 77.70 78.30 78.60 102.80
Matched spectrum intensities :
0.440000 0.050000 0.040000 0.050000 0.030000 0.020000 0.020000 0.010000 0.010000 0.060000 0.010000 0.020000
Not matched SDF shifts :
101.30 109.10 130.20 135.70 140.40 146.90 147.10 157.80

Rank: 36
ID : 1029
Name : LTS0143193
CAS : nan
MW : 487.41
Score : 0.6 (12/20 carbons)
Cumulated absolute difference : 4.03
Spectrum shifts left : 35/47

Matched spectrum shifts :
40.69 61.91 65.79 71.71 72.01 73.08 73.41 74.30 78.19 78.46 78.61 105.47
Matched SDF shifts :
40.70 61.10 66.50 72.40 72.40 72.70 73.10 74.20 78.30 78.40 78.90 105.30
Matched spectrum intensities :
0.880000 0.010000 0.040000 0.030000 0.020000 0.010000 0.020000 0.020000 0.060000 0.010000 0.010000 0.010000
Not matched SDF shifts :
101.30 109.90 130.20 135.70 137.80 146.90 147.10 157.80

Rank: 37
ID : 1748
Name : LTS0012525
CAS : nan
MW : 788.92
Score : 0.59 (23/39 carbons)
Cumulated absolute difference : 7.61
Spectrum shifts left : 24/47

Matched spectrum shifts :

40.05 40.69 40.90 41.33 41.55 61.91 62.64 63.59 64.35 64.41 69.21 70.63 72.01 73.41 74.51 77.11 78.19 78.46 78.61 83.32
84.35 103.43 105.47
Matched SDF shifts :
39.30 40.80 41.00 42.60 41.80 61.50 62.60 63.90 64.40 64.70 69.30 70.60 72.00 73.40 74.50 77.10 78.30 78.60 79.40 82.20
85.10 102.80 105.80
Matched spectrum intensities :
0.010000 0.880000 1.000000 0.440000 0.140000 0.010000 0.050000 0.010000 0.020000 0.020000 0.050000 0.050000 0.020000 0.020000
0.020000 0.010000 0.060000 0.010000 0.010000 0.020000 0.010000 0.020000 0.010000
Not matched SDF shifts :
15.00 17.00 17.20 22.10 27.90 30.70 31.80 32.70 35.80 38.20 47.00 49.70 55.60 57.20 69.50 110.30

Rank: 38
ID : 1290
Name : LTS0261262
CAS : nan
MW : 788.92
Score : 0.59 (23/39 carbons)
Cumulated absolute difference : 8.32
Spectrum shifts left : 24/47

Matched spectrum shifts :
40.05 40.69 40.90 41.33 41.55 61.91 62.64 63.49 64.35 64.41 69.21 70.63 71.26 73.80 74.51 75.71 77.11 78.19 78.46 78.61
84.35 103.43 105.47
Matched SDF shifts :
39.30 40.80 41.00 42.60 41.80 61.30 62.70 63.10 64.40 64.70 69.30 70.60 70.60 73.70 74.50 75.10 77.20 78.30 78.40 79.40
85.10 104.40 105.30
Matched spectrum intensities :
0.010000 0.880000 1.000000 0.440000 0.140000 0.010000 0.050000 0.010000 0.020000 0.020000 0.050000 0.050000 0.010000 0.020000
0.020000 0.010000 0.010000 0.060000 0.010000 0.010000 0.010000 0.020000 0.010000
Not matched SDF shifts :
14.80 17.00 17.20 22.10 27.90 30.70 31.80 32.70 34.70 38.20 47.00 49.80 55.60 57.20 81.30 110.30

Rank: 39
ID : 1513
Name : LTS0031032

CAS : nan
MW : 786.69
Score : 0.59 (20/34 carbons)
Cumulated absolute difference : 7.69
Spectrum shifts left : 27/47

Matched spectrum shifts :
62.64 65.79 69.21 70.63 71.26 71.32 71.71 72.01 75.71 76.25 76.75 77.22 78.19 78.61 93.67 98.34 99.50 103.43 105.47 134.36
Matched SDF shifts :
62.80 67.00 68.50 70.70 70.70 71.40 71.60 72.00 75.20 76.50 76.70 77.40 78.20 79.30 93.70 98.90 100.80 104.00 105.40 133.80
Matched spectrum intensities :
0.050000 0.040000 0.050000 0.050000 0.010000 0.050000 0.030000 0.020000 0.010000 0.030000 0.020000 0.010000 0.060000 0.010000 0.020000 0.030000 0.050000 0.020000 0.010000 0.010000
Not matched SDF shifts :
18.20 55.70 101.10 113.70 115.50 120.60 122.30 146.10 149.10 156.30 156.60 161.60 164.20 177.30

Rank: 40
ID : 1207
Name : LTS0222933
CAS : nan
MW : 573.5
Score : 0.58 (14/24 carbons)
Cumulated absolute difference : 4.81
Spectrum shifts left : 33/47

Matched spectrum shifts :
40.05 41.33 62.64 65.79 70.63 72.01 73.80 74.30 74.51 75.71 76.25 78.19 78.61 103.43
Matched SDF shifts :
39.30 41.30 62.60 66.50 70.60 72.30 73.80 74.00 75.20 75.70 75.30 77.90 79.20 103.30
Matched spectrum intensities :
0.010000 0.440000 0.050000 0.040000 0.050000 0.020000 0.020000 0.020000 0.020000 0.010000 0.030000 0.060000 0.010000 0.020000
Not matched SDF shifts :
20.80 101.30 109.10 130.20 135.70 140.40 146.90 147.10 157.80 171.30

Rank: 41
ID : 1091
Name : LTS0208698
CAS : nan
MW : 802.64
Score : 0.58 (19/33 carbons)
Cumulated absolute difference : 5.65
Spectrum shifts left : 28/47

Matched spectrum shifts :
61.91 62.64 71.26 71.32 71.71 73.80 74.30 74.51 75.71 76.75 77.11 77.22 78.19 78.61 93.67 99.50 103.43 105.47 134.36
Matched SDF shifts :
62.60 62.60 71.10 71.60 71.80 74.00 74.50 74.50 75.80 77.10 77.10 77.50 77.70 78.80 94.20 99.20 104.50 105.60 134.90
Matched spectrum intensities :
0.010000 0.050000 0.010000 0.050000 0.030000 0.020000 0.020000 0.020000 0.010000 0.020000 0.010000 0.010000 0.060000 0.010000 0.020000 0.050000 0.020000 0.010000 0.010000
Not matched SDF shifts :
100.40 102.20 115.50 115.60 121.30 121.60 145.20 148.80 156.00 159.10 160.80 163.00 171.90 179.50

Rank: 42
ID : 440
Name : LTS0081415
CAS : nan
MW : 772.66
Score : 0.58 (19/33 carbons)
Cumulated absolute difference : 6.61
Spectrum shifts left : 28/47

Matched spectrum shifts :
61.91 62.64 63.49 71.26 71.32 71.71 73.80 74.51 76.75 77.11 77.22 78.19 78.46 78.61 83.32 93.67 99.50 103.43 105.47
Matched SDF shifts :
62.50 62.60 62.60 71.20 71.10 71.60 73.90 74.50 77.10 77.80 77.50 77.90 78.80 78.70 82.80 94.40 99.10 102.60 105.40
Matched spectrum intensities :

0.010000 0.050000 0.010000 0.010000 0.050000 0.030000 0.020000 0.020000 0.020000 0.010000 0.010000 0.060000 0.010000 0.010000 0.020000 0.020000 0.050000 0.020000 0.010000
Not matched SDF shifts :
100.40 104.50 115.20 115.20 121.10 130.90 130.90 135.80 155.90 160.10 160.80 162.00 162.60 180.10

Rank: 43
ID : 1068
Name : LTS0074468
CAS : nan
MW : 756.66
Score : 0.58 (19/33 carbons)
Cumulated absolute difference : 8.76
Spectrum shifts left : 28/47

Matched spectrum shifts :
61.91 62.64 69.21 70.63 71.26 71.71 72.01 74.51 77.11 77.22 78.19 78.46 78.61 83.32 93.67 98.34 99.50 103.43 105.47
Matched SDF shifts :
61.10 62.70 68.50 70.70 70.70 72.00 72.40 75.10 77.20 77.70 78.30 78.40 78.80 82.20 93.70 98.90 100.40 104.40 104.70
Matched spectrum intensities :
0.010000 0.050000 0.050000 0.050000 0.010000 0.030000 0.020000 0.020000 0.010000 0.010000 0.060000 0.010000 0.010000 0.020000 0.020000 0.030000 0.050000 0.020000 0.010000
Not matched SDF shifts :
18.20 101.80 115.20 115.20 121.10 130.90 130.90 132.70 156.50 156.80 160.10 161.00 164.20 177.30

Rank: 44
ID : 836
Name : LTS0257423
CAS : nan
MW : 948.83
Score : 0.57 (23/40 carbons)
Cumulated absolute difference : 8.49
Spectrum shifts left : 24/47

Matched spectrum shifts :
62.64 65.79 69.21 70.63 71.26 71.32 71.71 72.01 74.51 75.71 76.25 76.75 77.11 77.22 78.19 78.46 78.61 93.67 98.34 99.50 103.43 105.47 134.36

Matched SDF shifts :
62.80 67.00 68.50 70.70 71.60 71.40 71.80 72.00 75.20 75.20 76.50 76.70 76.90 77.40 78.20 78.60 79.30 93.70 98.90 100.80 104.00 105.40 133.80
Matched spectrum intensities :
0.050000 0.040000 0.050000 0.050000 0.010000 0.050000 0.030000 0.020000 0.020000 0.010000 0.030000 0.020000 0.010000 0.010000 0.060000 0.010000 0.010000 0.020000 0.030000 0.050000 0.020000 0.010000 0.010000
Not matched SDF shifts :
18.20 55.70 67.00 70.70 101.10 105.00 113.70 115.50 120.60 122.30 146.10 149.10 156.30 156.60 161.60 164.20 177.30

Rank: 45
ID : 132
Name : LTS0060212
CAS : nan
MW : 611.59
Score : 0.57 (16/28 carbons)
Cumulated absolute difference : 6.51
Spectrum shifts left : 31/47

Matched spectrum shifts :
41.55 61.91 62.64 71.71 73.08 73.80 74.51 75.71 77.11 77.22 78.19 78.46 78.61 99.50 103.43 127.50
Matched SDF shifts :
42.30 62.60 62.60 71.60 72.60 73.80 74.50 75.70 77.90 77.50 77.90 78.50 79.20 100.70 103.10 126.60
Matched spectrum intensities :
0.140000 0.010000 0.050000 0.030000 0.010000 0.020000 0.020000 0.010000 0.010000 0.010000 0.060000 0.010000 0.010000 0.050000 0.020000 0.010000
Not matched SDF shifts :
28.20 53.40 56.80 60.60 104.00 106.80 109.10 114.70 129.10 143.60 147.00 147.40

Rank: 46
ID : 34
Name : LTS0160600
CAS : nan
MW : 611.59
Score : 0.57 (16/28 carbons)
Cumulated absolute difference : 7.52
Spectrum shifts left : 31/47

Matched spectrum shifts :
41.55 61.91 62.64 69.21 71.71 72.01 74.51 75.71 77.11 77.22 78.19 78.46 78.61 103.43 105.47 127.50
Matched SDF shifts :
42.30 61.10 62.70 69.10 71.70 72.40 75.10 75.40 78.30 78.30 78.30 78.40 78.60 104.40 105.30 126.60
Matched spectrum intensities :
0.140000 0.010000 0.050000 0.050000 0.030000 0.020000 0.020000 0.010000 0.010000 0.010000 0.060000 0.010000 0.010000 0.020000 0.010000 0.010000
Not matched SDF shifts :
28.20 53.40 56.80 60.60 101.30 106.80 109.90 114.70 129.10 143.60 146.80 147.40

Rank: 47
ID : 535
Name : LTS0129228
CAS : nan
MW : 1097.2
Score : 0.57 (29/51 carbons)
Cumulated absolute difference : 11.32
Spectrum shifts left : 18/47

Matched spectrum shifts :
40.69 40.90 41.33 41.55 61.91 62.64 63.49 63.59 65.79 69.21 70.63 71.26 72.01 73.08 73.80 74.30 74.51 75.71 76.25 76.75 77.11 77.22 78.19 78.46 78.61 84.35 99.50 103.43 105.47
Matched SDF shifts :
40.80 41.00 41.80 41.80 61.90 62.60 63.10 62.60 66.90 69.30 70.60 70.00 72.00 73.10 74.10 74.30 74.50 75.10 77.10 77.10 77.50 77.50 78.30 78.40 79.40 85.10 100.60 104.00 105.20
Matched spectrum intensities :
0.880000 1.000000 0.440000 0.140000 0.010000 0.050000 0.010000 0.010000 0.040000 0.050000 0.050000 0.010000 0.020000 0.010000 0.020000 0.020000 0.020000 0.010000 0.030000 0.020000 0.010000 0.010000 0.060000 0.010000 0.010000 0.010000 0.050000 0.020000 0.010000
Not matched SDF shifts :
14.80 17.00 17.20 17.20 22.10 29.10 30.40 30.70 31.60 32.70 35.80 38.20 47.00 49.70 55.60 57.20 61.50 69.50 69.50 81.30 102.80 109.20

Rank: 48

ID : 1405
Name : LTS0080305
CAS : nan
MW : 909.02
Score : 0.57 (26/46 carbons)
Cumulated absolute difference : 12.52
Spectrum shifts left : 21/47

Matched spectrum shifts :
40.05 40.26 40.69 40.90 41.33 41.55 61.91 62.64 63.49 69.21 71.71 72.01 73.08 73.41 73.80 74.30 74.51 75.71 77.22 78.19
78.46 78.61 103.43 105.47 127.50 134.36
Matched SDF shifts :
40.00 39.80 40.70 40.90 42.20 41.80 62.70 62.70 63.10 69.70 71.70 72.40 72.70 72.70 74.00 75.20 75.10 75.40 78.30 78.30
78.40 78.40 104.40 104.40 128.70 133.40
Matched spectrum intensities :
0.010000 0.150000 0.880000 1.000000 0.440000 0.140000 0.010000 0.050000 0.010000 0.050000 0.030000 0.020000 0.010000 0.020000
0.020000 0.020000 0.020000 0.010000 0.010000 0.060000 0.010000 0.010000 0.020000 0.010000 0.010000 0.010000
Not matched SDF shifts :
14.90 17.00 17.10 18.20 21.40 32.70 34.70 38.50 38.80 39.10 42.50 56.30 78.90 81.30 110.60 128.70 129.80 129.80 129.80
166.10

Rank: 49
ID : 1537
Name : LTS0235000
CAS : nan
MW : 550.59
Score : 0.56 (14/25 carbons)
Cumulated absolute difference : 5.46
Spectrum shifts left : 33/47

Matched spectrum shifts :
62.64 63.49 64.35 73.41 74.30 74.51 75.71 76.25 77.11 77.22 78.19 78.46 103.43 105.47
Matched SDF shifts :
62.60 62.80 64.40 73.60 74.20 75.20 75.70 75.20 77.00 77.90 78.00 78.20 103.30 104.20
Matched spectrum intensities :
0.050000 0.010000 0.020000 0.020000 0.020000 0.020000 0.010000 0.030000 0.010000 0.010000 0.060000 0.010000 0.020000 0.010000
Not matched SDF shifts :

21.10 26.20 28.10 28.10 34.90 36.10 47.00 49.10 123.60 147.80 202.60

Rank: 50
ID : 1538
Name : LTS0043591
CAS : nan
MW : 1129.24
Score : 0.56 (29/52 carbons)
Cumulated absolute difference : 11.78
Spectrum shifts left : 18/47

Matched spectrum shifts :
40.48 40.69 40.90 41.33 41.55 61.91 62.64 63.49 69.21 70.63 71.26 71.32 71.71 72.01 73.08 73.80 74.30 74.51 75.71 76.25 77.11 77.22 78.19 78.46 78.61 83.32 83.99 103.43 105.47
Matched SDF shifts :
40.50 40.80 40.90 42.50 42.20 61.90 62.30 63.10 69.70 70.60 70.70 70.60 70.50 72.10 72.70 74.20 74.50 75.00 75.20 75.10 78.10 77.50 78.20 78.40 78.30 83.00 83.50 103.40 105.10
Matched spectrum intensities :
0.440000 0.880000 1.000000 0.440000 0.140000 0.010000 0.050000 0.010000 0.050000 0.050000 0.010000 0.050000 0.030000 0.020000 0.010000 0.020000 0.020000 0.020000 0.010000 0.030000 0.010000 0.010000 0.060000 0.010000 0.010000 0.020000 0.010000 0.020000 0.010000
Not matched SDF shifts :
14.90 16.20 17.60 18.50 21.10 21.80 28.20 32.60 33.80 33.80 33.90 34.40 35.10 47.00 48.40 50.60 61.10 69.80 81.00 81.30 101.90 104.40 108.10

::

Rank: 1
ID : 1024
Name : LTS0087699
CAS : nan
MW : 152.15
Score : 1.0 (5/5 carbons)
Cumulated absolute difference : 0.51
Spectrum shifts left : 42/47

Matched spectrum shifts :
63.49 63.59 73.80 74.30 74.51
Matched SDF shifts :
63.50 63.50 74.00 74.40 74.40
Matched spectrum intensities :
0.010000 0.010000 0.020000 0.020000 0.020000

Rank: 2
ID : 5220
Name : LTS0023185
CAS : nan
MW : 152.15
Score : 1.0 (5/5 carbons)
Cumulated absolute difference : 0.51
Spectrum shifts left : 42/47

Matched spectrum shifts :
63.49 63.59 73.80 74.30 74.51
Matched SDF shifts :
63.50 63.50 74.00 74.40 74.40
Matched spectrum intensities :
0.010000 0.010000 0.020000 0.020000 0.020000

Rank: 3
ID : 4101
Name : LTS0231470
CAS : nan
MW : 193.2
Score : 1.0 (7/7 carbons)
Cumulated absolute difference : 1.13
Spectrum shifts left : 40/47

Matched spectrum shifts :
61.91 62.64 63.49 63.59 69.21 71.32 71.71
Matched SDF shifts :
61.90 62.60 63.20 63.20 69.10 71.60 71.70
Matched spectrum intensities :
0.010000 0.050000 0.010000 0.010000 0.050000 0.050000 0.030000

Rank: 4
ID : 2371
Name : LTS0161003
CAS : nan
MW : 193.2
Score : 1.0 (7/7 carbons)

Cumulated absolute difference : 1.19
Spectrum shifts left : 40/47

Matched spectrum shifts :
61.91 63.49 63.59 65.15 71.71 76.25 77.11
Matched SDF shifts :
61.90 63.20 63.20 65.00 71.70 76.50 77.20
Matched spectrum intensities :
0.010000 0.010000 0.010000 0.010000 0.030000 0.030000 0.010000

Rank: 5
ID : 2608
Name : LTS0087238
CAS : nan
MW : 193.2
Score : 1.0 (7/7 carbons)
Cumulated absolute difference : 1.51
Spectrum shifts left : 40/47

Matched spectrum shifts :
61.91 63.49 63.59 65.15 71.71 75.71 76.25
Matched SDF shifts :
61.90 63.20 63.20 65.00 71.70 75.30 76.50
Matched spectrum intensities :
0.010000 0.010000 0.010000 0.010000 0.030000 0.010000 0.030000

Rank: 6
ID : 3875

Name : LTS0263530
CAS : nan
MW : 193.2
Score : 1.0 (7/7 carbons)
Cumulated absolute difference : 1.75
Spectrum shifts left : 40/47

Matched spectrum shifts :
61.91 62.64 63.49 63.59 72.01 73.08 73.41
Matched SDF shifts :
62.50 62.50 63.20 63.20 72.00 73.40 73.40
Matched spectrum intensities :
0.010000 0.050000 0.010000 0.010000 0.020000 0.010000 0.020000

Rank: 7
ID : 1408
Name : LTS0107522
CAS : nan
MW : 180.16
Score : 1.0 (6/6 carbons)
Cumulated absolute difference : 1.81
Spectrum shifts left : 41/47

Matched spectrum shifts :
61.91 70.63 72.01 73.08 73.41 93.67
Matched SDF shifts :
61.90 70.70 72.40 72.80 73.50 92.70
Matched spectrum intensities :
0.010000 0.050000 0.020000 0.010000 0.020000 0.020000

Rank: 8
ID : 1787
Name : LTS0204783
CAS : nan
MW : 193.2
Score : 1.0 (7/7 carbons)
Cumulated absolute difference : 2.07
Spectrum shifts left : 40/47

Matched spectrum shifts :
61.91 63.49 63.59 65.15 69.21 70.63 76.25
Matched SDF shifts :
61.90 63.20 63.20 65.00 68.50 70.90 76.50
Matched spectrum intensities :
0.010000 0.010000 0.010000 0.010000 0.050000 0.050000 0.030000

Rank: 9
ID : 122
Name : LTS0132398
CAS : nan
MW : 180.16
Score : 1.0 (6/6 carbons)
Cumulated absolute difference : 2.11
Spectrum shifts left : 41/47

Matched spectrum shifts :
61.91 70.63 72.01 73.08 73.41 93.67
Matched SDF shifts :
61.60 70.70 72.40 72.80 73.50 92.70

Matched spectrum intensities :
0.010000 0.050000 0.020000 0.010000 0.020000 0.020000

Rank: 10
ID : 1831
Name : LTS0272557
CAS : nan
MW : 342.3
Score : 1.0 (12/12 carbons)
Cumulated absolute difference : 2.12
Spectrum shifts left : 35/47

Matched spectrum shifts :
61.91 63.59 64.35 70.63 72.01 73.41 74.30 75.71 78.19 83.32 93.67 105.47
Matched SDF shifts :
61.80 63.70 64.30 70.20 72.00 73.60 74.40 75.80 78.20 83.10 92.90 105.50
Matched spectrum intensities :
0.010000 0.010000 0.020000 0.050000 0.020000 0.020000 0.020000 0.010000 0.060000 0.020000 0.020000 0.010000

Rank: 11
ID : 1061
Name : LTS0089262
CAS : nan
MW : 180.16
Score : 1.0 (6/6 carbons)
Cumulated absolute difference : 2.20
Spectrum shifts left : 41/47

Matched spectrum shifts :
61.91 71.26 72.01 73.08 73.41 93.67
Matched SDF shifts :
61.60 71.10 72.40 72.80 73.50 92.70
Matched spectrum intensities :
0.010000 0.010000 0.020000 0.010000 0.020000 0.020000

Rank: 12
ID : 2414
Name : LTS0231627
CAS : nan
MW : 180.16
Score : 1.0 (6/6 carbons)
Cumulated absolute difference : 2.20
Spectrum shifts left : 41/47

Matched spectrum shifts :
61.91 71.26 72.01 73.08 73.41 93.67
Matched SDF shifts :
61.60 71.10 72.40 72.80 73.50 92.70
Matched spectrum intensities :
0.010000 0.010000 0.020000 0.010000 0.020000 0.020000

Rank: 13
ID : 4900
Name : LTS0013597
CAS : nan
MW : 180.16
Score : 1.0 (6/6 carbons)

Cumulated absolute difference : 2.20
Spectrum shifts left : 41/47

Matched spectrum shifts :
61.91 71.26 72.01 73.08 73.41 93.67
Matched SDF shifts :
61.60 71.10 72.40 72.80 73.50 92.70
Matched spectrum intensities :
0.010000 0.010000 0.020000 0.010000 0.020000 0.020000

Rank: 14
ID : 5155
Name : LTS0017054
CAS : nan
MW : 207.22
Score : 1.0 (8/8 carbons)
Cumulated absolute difference : 2.47
Spectrum shifts left : 39/47

Matched spectrum shifts :
40.05 61.91 62.64 64.35 64.41 71.71 72.01 76.25
Matched SDF shifts :
39.60 62.00 62.40 64.20 64.20 72.40 72.40 76.50
Matched spectrum intensities :
0.010000 0.010000 0.050000 0.020000 0.020000 0.030000 0.020000 0.030000

Rank: 15
ID : 1296
Name : LTS0043712
CAS : nan
MW : 150.13

Score : 1.0 (5/5 carbons)
Cumulated absolute difference : 2.48
Spectrum shifts left : 42/47

Matched spectrum shifts :
65.79 70.63 72.01 73.08 93.67
Matched SDF shifts :
66.50 70.30 72.20 73.10 94.90
Matched spectrum intensities :
0.040000 0.050000 0.020000 0.010000 0.020000

Rank: 16
ID : 2887
Name : LTS0132381
CAS : nan
MW : 150.13
Score : 1.0 (5/5 carbons)
Cumulated absolute difference : 2.48
Spectrum shifts left : 42/47

Matched spectrum shifts :
65.79 70.63 72.01 73.08 93.67
Matched SDF shifts :
66.50 70.30 72.20 73.10 94.90
Matched spectrum intensities :
0.040000 0.050000 0.020000 0.010000 0.020000

Rank: 17
ID : 4219

Name : LTS0019868
CAS : nan
MW : 193.2
Score : 1.0 (7/7 carbons)
Cumulated absolute difference : 2.52
Spectrum shifts left : 40/47

Matched spectrum shifts :
61.91 63.49 63.59 65.15 73.08 74.51 75.71
Matched SDF shifts :
61.90 63.20 63.20 65.00 72.60 75.30 75.30
Matched spectrum intensities :
0.010000 0.010000 0.010000 0.010000 0.010000 0.020000 0.010000

Rank: 18
ID : 2031
Name : LTS0199986
CAS : nan
MW : 182.17
Score : 1.0 (6/6 carbons)
Cumulated absolute difference : 2.78
Spectrum shifts left : 41/47

Matched spectrum shifts :
65.15 65.79 73.08 73.41 74.30 74.51
Matched SDF shifts :
66.30 66.30 73.40 73.40 74.80 74.80
Matched spectrum intensities :
0.010000 0.040000 0.010000 0.020000 0.020000 0.020000

Rank: 19
ID : 1622
Name : LTS0150163
CAS : nan
MW : 122.12
Score : 1.0 (4/4 carbons)
Cumulated absolute difference : 2.82
Spectrum shifts left : 43/47

Matched spectrum shifts :
65.15 65.79 75.71 76.25
Matched SDF shifts :
66.40 66.40 75.50 75.50
Matched spectrum intensities :
0.010000 0.040000 0.010000 0.030000

Rank: 20
ID : 5199
Name : LTS0003058
CAS : nan
MW : 122.12
Score : 1.0 (4/4 carbons)
Cumulated absolute difference : 2.82
Spectrum shifts left : 43/47

Matched spectrum shifts :
65.15 65.79 75.71 76.25
Matched SDF shifts :
66.40 66.40 75.50 75.50

Matched spectrum intensities :
0.010000 0.040000 0.010000 0.030000

Rank: 21
ID : 4623
Name : LTS0192514
CAS : nan
MW : 355.34
Score : 1.0 (13/13 carbons)
Cumulated absolute difference : 3.61
Spectrum shifts left : 34/47

Matched spectrum shifts :
61.91 62.64 63.49 63.59 69.21 71.32 71.71 73.41 73.80 76.75 77.11 77.22 105.47
Matched SDF shifts :
61.90 62.80 63.20 63.20 69.60 71.70 71.70 73.70 73.70 76.90 77.20 77.60 104.50
Matched spectrum intensities :
0.010000 0.050000 0.010000 0.010000 0.050000 0.050000 0.030000 0.020000 0.020000 0.020000 0.010000
0.010000 0.010000

Rank: 22
ID : 2806
Name : LTS0247838
CAS : nan
MW : 355.34
Score : 1.0 (13/13 carbons)
Cumulated absolute difference : 4.00
Spectrum shifts left : 34/47

Matched spectrum shifts :
61.91 62.64 63.49 63.59 69.21 71.32 71.71 73.80 74.51 76.25 77.11 78.19 105.47
Matched SDF shifts :
61.90 62.30 63.20 63.20 69.60 71.70 71.70 73.70 75.10 76.50 77.20 78.00 104.50
Matched spectrum intensities :
0.010000 0.050000 0.010000 0.010000 0.050000 0.050000 0.030000 0.020000 0.020000 0.030000 0.010000 0.060000 0.010000

Rank: 23
ID : 1819
Name : LTS0139126
CAS : nan
MW : 369.37
Score : 1.0 (14/14 carbons)
Cumulated absolute difference : 4.49
Spectrum shifts left : 33/47

Matched spectrum shifts :
40.05 61.91 62.64 64.35 64.41 69.21 71.32 71.71 72.01 73.08 73.41 76.25 77.11 105.47
Matched SDF shifts :
39.60 62.30 62.40 64.20 64.20 69.60 71.50 71.70 72.40 72.40 73.50 76.50 77.20 104.50
Matched spectrum intensities :
0.010000 0.010000 0.050000 0.020000 0.020000 0.050000 0.050000 0.030000 0.020000 0.010000 0.020000 0.030000 0.010000 0.010000

Rank: 24
ID : 3904
Name : LTS0210079
CAS : nan

MW : 342.3
Score : 0.92 (11/12 carbons)
Cumulated absolute difference : 2.83
Spectrum shifts left : 36/47

Matched spectrum shifts :
61.91 62.64 69.21 73.41 73.80 74.30 74.51 78.19 78.46 78.61 103.43
Matched SDF shifts :
61.10 62.60 69.50 73.40 74.00 74.30 74.10 78.30 78.60 79.40 103.40
Matched spectrum intensities :
0.010000 0.050000 0.050000 0.020000 0.020000 0.020000 0.020000 0.060000 0.010000 0.010000 0.020000
Not matched SDF shifts :
95.30

Rank: 25
ID : 945
Name : LTS0100655
CAS : nan
MW : 191.23
Score : 0.88 (7/8 carbons)
Cumulated absolute difference : 1.53
Spectrum shifts left : 40/47

Matched spectrum shifts :
40.05 61.91 62.64 64.35 64.41 71.32 71.71
Matched SDF shifts :
39.60 62.00 62.40 64.20 64.20 71.70 71.70
Matched spectrum intensities :
0.010000 0.010000 0.050000 0.020000 0.020000 0.050000 0.030000
Not matched SDF shifts :

35.10

Rank: 26
ID : 2079
Name : LTS0141759
CAS : nan
MW : 208.21
Score : 0.88 (7/8 carbons)
Cumulated absolute difference : 3.07
Spectrum shifts left : 40/47

Matched spectrum shifts :
61.91 64.41 69.21 74.51 76.25 77.11 105.47
Matched SDF shifts :
62.30 64.70 69.70 75.10 76.50 77.20 104.50
Matched spectrum intensities :
0.010000 0.020000 0.050000 0.020000 0.030000 0.010000 0.010000
Not matched SDF shifts :
15.10

Rank: 27
ID : 4093
Name : LTS0241114
CAS : nan
MW : 180.16
Score : 0.83 (5/6 carbons)
Cumulated absolute difference : 0.80
Spectrum shifts left : 42/47

Matched spectrum shifts :

64.35 65.79 73.08 75.71 76.25
Matched SDF shifts :
64.40 66.20 73.00 75.50 76.20
Matched spectrum intensities :
0.020000 0.040000 0.010000 0.010000 0.030000
Not matched SDF shifts :
217.40

Rank: 28
ID : 4620
Name : LTS0245637
CAS : nan
MW : 163.17
Score : 0.83 (5/6 carbons)
Cumulated absolute difference : 1.10
Spectrum shifts left : 42/47

Matched spectrum shifts :
62.64 63.49 69.21 71.71 76.75
Matched SDF shifts :
62.60 63.20 68.50 71.70 76.80
Matched spectrum intensities :
0.050000 0.010000 0.050000 0.030000 0.020000
Not matched SDF shifts :
46.40

Rank: 29
ID : 4436
Name : LTS0057096

CAS : nan
MW : 196.16
Score : 0.83 (5/6 carbons)
Cumulated absolute difference : 1.16
Spectrum shifts left : 42/47

Matched spectrum shifts :
65.79 70.63 73.08 73.41 74.51
Matched SDF shifts :
66.30 70.70 72.80 73.40 74.80
Matched spectrum intensities :
0.040000 0.050000 0.010000 0.020000 0.020000
Not matched SDF shifts :
174.00

Rank: 30
ID : 1722
Name : LTS0262158
CAS : nan
MW : 180.16
Score : 0.83 (5/6 carbons)
Cumulated absolute difference : 1.39
Spectrum shifts left : 42/47

Matched spectrum shifts :
65.79 73.08 73.41 73.80 74.51
Matched SDF shifts :
66.30 72.80 73.40 73.50 74.80
Matched spectrum intensities :
0.040000 0.010000 0.020000 0.020000 0.020000

Not matched SDF shifts :
200.80

Rank: 31
ID : 3783
Name : LTS0084516
CAS : nan
MW : 164.16
Score : 0.83 (5/6 carbons)
Cumulated absolute difference : 1.41
Spectrum shifts left : 42/47

Matched spectrum shifts :
69.21 72.01 73.08 73.80 93.67
Matched SDF shifts :
69.20 72.20 72.80 73.70 94.50
Matched spectrum intensities :
0.050000 0.020000 0.010000 0.020000 0.020000
Not matched SDF shifts :
18.30

Rank: 32
ID : 3798
Name : LTS0223750
CAS : nan
MW : 196.16
Score : 0.83 (5/6 carbons)
Cumulated absolute difference : 1.48
Spectrum shifts left : 42/47

Matched spectrum shifts :
65.79 72.01 73.08 73.41 74.51
Matched SDF shifts :
66.30 72.20 72.60 73.40 74.80
Matched spectrum intensities :
0.040000 0.020000 0.010000 0.020000 0.020000
Not matched SDF shifts :
174.00

Rank: 33
ID : 6125
Name : LTS0259277
CAS : nan
MW : 180.16
Score : 0.83 (5/6 carbons)
Cumulated absolute difference : 1.58
Spectrum shifts left : 42/47

Matched spectrum shifts :
63.49 63.59 69.21 70.63 98.34
Matched SDF shifts :
63.30 63.80 69.20 69.70 98.10
Matched spectrum intensities :
0.010000 0.010000 0.050000 0.050000 0.030000
Not matched SDF shifts :
67.50

Rank: 34

ID : 2573
Name : LTS0239546
CAS : nan
MW : 164.16
Score : 0.83 (5/6 carbons)
Cumulated absolute difference : 1.95
Spectrum shifts left : 42/47

Matched spectrum shifts :
69.21 73.08 73.80 76.25 93.67
Matched SDF shifts :
69.90 72.80 73.90 76.30 94.50
Matched spectrum intensities :
0.050000 0.010000 0.020000 0.030000 0.020000
Not matched SDF shifts :
18.20

Rank: 35
ID : 1323
Name : LTS0175812
CAS : nan
MW : 164.16
Score : 0.83 (5/6 carbons)
Cumulated absolute difference : 2.09
Spectrum shifts left : 42/47

Matched spectrum shifts :
70.63 73.08 74.30 76.25 93.67
Matched SDF shifts :
70.00 72.80 74.40 76.50 94.50

Matched spectrum intensities :
0.050000 0.010000 0.020000 0.030000 0.020000
Not matched SDF shifts :
18.30

Rank: 36
ID : 928
Name : LTS0080274
CAS : nan
MW : 342.3
Score : 0.83 (10/12 carbons)
Cumulated absolute difference : 2.11
Spectrum shifts left : 37/47

Matched spectrum shifts :
62.64 69.21 71.26 71.32 72.01 73.80 75.71 77.22 78.19 103.43
Matched SDF shifts :
62.60 69.50 71.20 71.20 72.40 73.80 75.70 77.50 77.90 102.80
Matched spectrum intensities :
0.050000 0.050000 0.010000 0.050000 0.020000 0.020000 0.010000 0.010000 0.060000 0.020000
Not matched SDF shifts :
68.20 68.20

Rank: 37
ID : 2394
Name : LTS0165930
CAS : nan
MW : 180.16

Score : 0.83 (5/6 carbons)
Cumulated absolute difference : 2.45
Spectrum shifts left : 42/47

Matched spectrum shifts :
71.71 72.01 73.08 73.41 73.80
Matched SDF shifts :
72.70 72.70 72.70 73.70 73.70
Matched spectrum intensities :
0.030000 0.020000 0.010000 0.020000 0.020000
Not matched SDF shifts :
72.70

Rank: 38
ID : 3919
Name : LTS0220516
CAS : nan
MW : 342.3
Score : 0.83 (10/12 carbons)
Cumulated absolute difference : 2.49
Spectrum shifts left : 37/47

Matched spectrum shifts :
61.91 71.32 73.41 73.80 74.30 74.51 76.25 77.11 78.61 103.43
Matched SDF shifts :
61.10 71.40 73.40 74.00 74.30 74.10 76.30 77.00 79.40 103.40
Matched spectrum intensities :
0.010000 0.050000 0.020000 0.020000 0.020000 0.020000 0.030000 0.010000 0.010000 0.020000
Not matched SDF shifts :
61.10 95.30

Rank: 39
ID : 3134
Name : LTS0188871
CAS : nan
MW : 342.3
Score : 0.83 (10/12 carbons)
Cumulated absolute difference : 3.80
Spectrum shifts left : 37/47

Matched spectrum shifts :
61.91 69.21 70.63 71.26 71.32 72.01 74.30 75.71 77.22 78.19
Matched SDF shifts :
61.10 68.20 71.20 71.20 71.40 72.40 74.20 75.70 77.50 77.70
Matched spectrum intensities :
0.010000 0.050000 0.050000 0.010000 0.050000 0.020000 0.020000 0.010000 0.010000 0.060000
Not matched SDF shifts :
68.20 101.80

Rank: 40
ID : 1559
Name : LTS0047771
CAS : nan
MW : 180.16
Score : 0.83 (5/6 carbons)
Cumulated absolute difference : 3.87
Spectrum shifts left : 42/47

Matched spectrum shifts :

71.71 72.01 73.08 73.41 73.80
Matched SDF shifts :
72.70 72.70 72.70 72.70 72.70
Matched spectrum intensities :
0.030000 0.020000 0.010000 0.020000 0.020000
Not matched SDF shifts :
72.70

Rank: 41
ID : 4471
Name : LTS0257088
CAS : nan
MW : 180.16
Score : 0.83 (5/6 carbons)
Cumulated absolute difference : 3.87
Spectrum shifts left : 42/47

Matched spectrum shifts :
71.71 72.01 73.08 73.41 73.80
Matched SDF shifts :
72.70 72.70 72.70 72.70 72.70
Matched spectrum intensities :
0.030000 0.020000 0.010000 0.020000 0.020000
Not matched SDF shifts :
72.70

Rank: 42
ID : 1908
Name : LTS0169607

CAS : nan
MW : 326.3
Score : 0.83 (10/12 carbons)
Cumulated absolute difference : 4.99
Spectrum shifts left : 37/47

Matched spectrum shifts :
69.21 70.63 71.26 71.71 72.01 73.08 73.41 74.51 93.67 99.50
Matched SDF shifts :
68.30 70.40 70.70 71.90 72.50 72.80 73.30 75.10 94.50 100.30
Matched spectrum intensities :
0.050000 0.050000 0.010000 0.030000 0.020000 0.010000 0.020000 0.020000 0.020000 0.050000
Not matched SDF shifts :
18.20 68.00

Rank: 43
ID : 1042
Name : LTS0023485
CAS : nan
MW : 409.39
Score : 0.81 (13/16 carbons)
Cumulated absolute difference : 3.11
Spectrum shifts left : 34/47

Matched spectrum shifts :
61.91 69.21 71.26 71.71 72.01 73.80 74.51 76.25 76.75 77.22 98.34 103.43 118.03
Matched SDF shifts :
62.30 69.70 71.20 71.90 72.00 73.90 74.80 76.30 76.40 77.30 98.90 103.60 118.40
Matched spectrum intensities :

0.010000 0.050000 0.010000 0.030000 0.020000 0.020000 0.020000 0.030000 0.020000 0.010000 0.030000
0.020000 0.010000
Not matched SDF shifts :
26.70 26.70 67.30

Rank: 44
ID : 636
Name : LTS0085513
CAS : nan
MW : 150.13
Score : 0.8 (4/5 carbons)
Cumulated absolute difference : 0.27
Spectrum shifts left : 43/47

Matched spectrum shifts :
64.35 73.08 73.41 76.25
Matched SDF shifts :
64.40 73.00 73.50 76.20
Matched spectrum intensities :
0.020000 0.010000 0.020000 0.030000
Not matched SDF shifts :
200.80

Rank: 45
ID : 3719
Name : LTS0206216
CAS : nan
MW : 247.25
Score : 0.8 (8/10 carbons)

Cumulated absolute difference : 1.73
Spectrum shifts left : 39/47

Matched spectrum shifts :
62.64 71.71 72.01 74.51 78.19 78.46 98.34 118.03
Matched SDF shifts :
62.60 71.70 71.90 74.80 78.10 78.20 98.90 118.40
Matched spectrum intensities :
0.050000 0.030000 0.020000 0.020000 0.060000 0.010000 0.030000 0.010000
Not matched SDF shifts :
26.70 26.70

Rank: 46
ID : 5750
Name : LTS0237794
CAS : nan
MW : 296.23
Score : 0.8 (8/10 carbons)
Cumulated absolute difference : 1.78
Spectrum shifts left : 39/47

Matched spectrum shifts :
40.05 62.64 74.30 74.51 75.71 76.25 78.19 103.43
Matched SDF shifts :
39.20 62.60 74.50 74.50 75.70 76.30 77.90 103.10
Matched spectrum intensities :
0.010000 0.050000 0.020000 0.020000 0.010000 0.030000 0.060000 0.020000

Not matched SDF shifts :
174.80 174.90

Rank: 47
ID : 5702
Name : LTS0032647
CAS : nan
MW : 247.25
Score : 0.8 (8/10 carbons)
Cumulated absolute difference : 1.78
Spectrum shifts left : 39/47

Matched spectrum shifts :
62.64 71.71 72.01 73.80 78.19 78.46 99.50 118.03
Matched SDF shifts :
62.80 71.60 71.90 74.10 78.10 78.50 100.10 118.40
Matched spectrum intensities :
0.050000 0.030000 0.020000 0.020000 0.060000 0.010000 0.050000 0.010000
Not matched SDF shifts :
26.70 26.70

Rank: 48
ID : 2308
Name : LTS0153167
CAS : nan
MW : 423.41
Score : 0.76 (13/17 carbons)

Cumulated absolute difference : 2.25
Spectrum shifts left : 34/47

Matched spectrum shifts :
62.64 69.21 71.26 71.71 72.01 73.08 73.80 74.30 76.25 78.19 78.46 103.43 118.03
Matched SDF shifts :
62.80 69.80 71.20 71.70 72.00 73.10 73.90 74.00 76.40 78.00 78.00 103.60 118.00
Matched spectrum intensities :
0.050000 0.050000 0.010000 0.030000 0.020000 0.010000 0.020000 0.020000 0.030000 0.060000 0.010000 0.020000 0.010000
Not matched SDF shifts :
7.80 23.60 32.60 97.00

Rank: 49
ID : 2539
Name : LTS0109641
CAS : nan
MW : 411.45
Score : 0.76 (13/17 carbons)
Cumulated absolute difference : 4.97
Spectrum shifts left : 34/47

Matched spectrum shifts :
61.91 62.64 64.35 64.41 69.21 70.63 71.32 71.71 72.01 73.41 76.25 77.11 105.47
Matched SDF shifts :
62.30 62.40 64.20 64.20 69.60 69.70 71.50 72.40 72.40 73.50 76.50 77.20 104.50
Matched spectrum intensities :
0.010000 0.050000 0.020000 0.020000 0.050000 0.050000 0.050000 0.030000 0.020000 0.020000 0.030000 0.010000 0.010000
Not matched SDF shifts :

13.80 19.90 30.10 49.20

Rank: 50
ID : 305
Name : LTS0104449
CAS : nan
MW : 191.23
Score : 0.75 (6/8 carbons)
Cumulated absolute difference : 1.25
Spectrum shifts left : 41/47

Matched spectrum shifts :
40.05 61.91 62.64 64.35 64.41 69.21
Matched SDF shifts :
39.60 62.00 62.40 64.20 64.20 69.10
Matched spectrum intensities :
0.010000 0.010000 0.050000 0.020000 0.020000 0.050000
Not matched SDF shifts :
32.40 68.70

Rank: 51
ID : 3234
Name : LTS0078874
CAS : nan
MW : 191.23
Score : 0.75 (6/8 carbons)
Cumulated absolute difference : 2.27
Spectrum shifts left : 41/47

Matched spectrum shifts :
40.05 62.64 64.35 72.01 76.25 77.11
Matched SDF shifts :
38.90 62.40 64.20 72.40 76.50 77.20
Matched spectrum intensities :
0.010000 0.050000 0.020000 0.020000 0.030000 0.010000
Not matched SDF shifts :
13.20 49.40

Rank: 52
ID : 1862
Name : LTS0126731
CAS : nan
MW : 261.27
Score : 0.73 (8/11 carbons)
Cumulated absolute difference : 1.01
Spectrum shifts left : 39/47

Matched spectrum shifts :
62.64 71.71 73.08 74.51 76.25 78.19 98.34 118.03
Matched SDF shifts :
62.60 71.70 73.10 74.80 76.20 78.20 98.90 118.00
Matched spectrum intensities :
0.050000 0.030000 0.010000 0.020000 0.030000 0.060000 0.030000 0.010000
Not matched SDF shifts :
7.80 23.60 32.60

Rank: 53
ID : 3763

Name : LTS0070973
CAS : nan
MW : 261.27
Score : 0.73 (8/11 carbons)
Cumulated absolute difference : 1.01
Spectrum shifts left : 39/47

Matched spectrum shifts :
62.64 71.71 73.08 74.51 76.25 78.19 98.34 118.03
Matched SDF shifts :
62.60 71.70 73.10 74.80 76.20 78.20 98.90 118.00
Matched spectrum intensities :
0.050000 0.030000 0.010000 0.020000 0.030000 0.060000 0.030000 0.010000
Not matched SDF shifts :
7.80 23.60 32.60

Rank: 54
ID : 4081
Name : LTS0184222
CAS : nan
MW : 261.27
Score : 0.73 (8/11 carbons)
Cumulated absolute difference : 1.30
Spectrum shifts left : 39/47

Matched spectrum shifts :
62.64 71.71 73.08 74.51 78.19 78.46 98.34 118.03
Matched SDF shifts :
62.60 71.70 73.10 74.80 78.10 78.20 98.90 118.00
Matched spectrum intensities :

0.050000 0.030000 0.010000 0.020000 0.060000 0.010000 0.030000 0.010000
Not matched SDF shifts :
7.80 23.60 32.60

Rank: 55
ID : 3833
Name : LTS0211301
CAS : nan
MW : 261.27
Score : 0.73 (8/11 carbons)
Cumulated absolute difference : 1.35
Spectrum shifts left : 39/47

Matched spectrum shifts :
62.64 71.71 73.08 73.80 78.19 78.46 99.50 118.03
Matched SDF shifts :
62.80 71.60 73.10 74.10 78.10 78.50 100.10 118.00
Matched spectrum intensities :
0.050000 0.030000 0.010000 0.020000 0.060000 0.010000 0.050000 0.010000
Not matched SDF shifts :
7.80 23.60 32.60

Rank: 56
ID : 1856
Name : LTS0178194
CAS : nan
MW : 277.27
Score : 0.73 (8/11 carbons)
Cumulated absolute difference : 1.98

Spectrum shifts left : 39/47

Matched spectrum shifts :
61.91 64.35 70.63 74.30 76.75 78.19 99.50 118.03
Matched SDF shifts :
62.20 64.40 70.70 74.30 76.80 77.80 100.00 117.40
Matched spectrum intensities :
0.010000 0.020000 0.050000 0.020000 0.020000 0.060000 0.050000 0.010000
Not matched SDF shifts :
11.70 26.40 34.90

Rank: 57
ID : 1367
Name : LTS0045615
CAS : nan
MW : 261.27
Score : 0.73 (8/11 carbons)
Cumulated absolute difference : 3.09
Spectrum shifts left : 39/47

Matched spectrum shifts :
62.64 69.21 74.30 74.51 78.19 78.46 105.47 118.03
Matched SDF shifts :
62.80 69.80 74.20 75.10 78.10 78.20 104.80 117.40
Matched spectrum intensities :
0.050000 0.050000 0.020000 0.020000 0.060000 0.010000 0.010000 0.010000
Not matched SDF shifts :
11.70 26.40 34.90

Rank: 58
ID : 944
Name : LTS0046726
CAS : nan
MW : 484.5
Score : 0.72 (13/18 carbons)
Cumulated absolute difference : 3.90
Spectrum shifts left : 34/47

Matched spectrum shifts :
40.48 61.91 69.21 71.26 71.32 71.71 73.08 73.41 74.51 75.71 76.25 78.46 103.43
Matched SDF shifts :
40.40 61.60 69.90 71.10 71.50 71.60 72.90 73.10 74.70 75.30 76.50 78.40 104.40
Matched spectrum intensities :
0.440000 0.010000 0.050000 0.010000 0.050000 0.030000 0.010000 0.020000 0.020000 0.010000 0.030000 0.010000 0.020000
Not matched SDF shifts :
43.60 52.60 56.60 56.70 101.50

Rank: 59
ID : 989
Name : LTS0013262
CAS : nan
MW : 194.18
Score : 0.71 (5/7 carbons)
Cumulated absolute difference : 0.58
Spectrum shifts left : 42/47

Matched spectrum shifts :
69.21 71.26 71.32 72.01 74.30

Matched SDF shifts :
69.20 71.20 71.20 72.40 74.30
Matched spectrum intensities :
0.050000 0.010000 0.050000 0.020000 0.020000
Not matched SDF shifts :
57.50 69.20

Rank: 60
ID : 5265
Name : LTS0065138
CAS : nan
MW : 194.18
Score : 0.71 (5/7 carbons)
Cumulated absolute difference : 0.58
Spectrum shifts left : 42/47

Matched spectrum shifts :
69.21 71.26 71.32 72.01 74.30
Matched SDF shifts :
69.20 71.20 71.20 72.40 74.30
Matched spectrum intensities :
0.050000 0.010000 0.050000 0.020000 0.020000
Not matched SDF shifts :
57.50 69.20

Rank: 61
ID : 3035
Name : LTS0249934
CAS : nan

MW : 194.18
Score : 0.71 (5/7 carbons)
Cumulated absolute difference : 0.66
Spectrum shifts left : 42/47

Matched spectrum shifts :
69.21 71.26 71.32 72.01 73.08
Matched SDF shifts :
69.20 71.20 71.20 72.40 73.00
Matched spectrum intensities :
0.050000 0.010000 0.050000 0.020000 0.010000
Not matched SDF shifts :
59.10 69.20

Rank: 62
ID : 6248
Name : LTS0051765
CAS : nan
MW : 194.18
Score : 0.71 (5/7 carbons)
Cumulated absolute difference : 0.66
Spectrum shifts left : 42/47

Matched spectrum shifts :
69.21 71.26 71.32 72.01 73.08
Matched SDF shifts :
69.20 71.20 71.20 72.40 73.00
Matched spectrum intensities :
0.050000 0.010000 0.050000 0.020000 0.010000
Not matched SDF shifts :

59.10   69.20

Rank: 63
ID : 3638
Name : LTS0220361
CAS : nan
MW : 194.18
Score : 0.71 (5/7 carbons)
Cumulated absolute difference : 0.87
Spectrum shifts left : 42/47

Matched spectrum shifts :
69.21   71.26   71.32   72.01   78.61
Matched SDF shifts :
69.20   71.20   71.20   72.40   78.90
Matched spectrum intensities :
0.050000   0.010000   0.050000   0.020000   0.010000
Not matched SDF shifts :
58.10   69.20

Rank: 64
ID : 4694
Name : LTS0045051
CAS : nan
MW : 177.2
Score : 0.71 (5/7 carbons)
Cumulated absolute difference : 0.91
Spectrum shifts left : 42/47

Matched spectrum shifts :
61.91 63.49 70.63 76.25 77.11
Matched SDF shifts :
61.90 63.20 70.90 76.50 77.20
Matched spectrum intensities :
0.010000 0.010000 0.050000 0.030000 0.010000
Not matched SDF shifts :
19.10 52.20

Rank: 65
ID : 5272
Name : LTS0013020
CAS : nan
MW : 245.23
Score : 0.7 (7/10 carbons)
Cumulated absolute difference : 1.95
Spectrum shifts left : 40/47

Matched spectrum shifts :
62.64 64.35 71.71 75.71 78.19 78.46 103.43
Matched SDF shifts :
62.80 64.40 71.70 75.20 78.00 78.20 104.20
Matched spectrum intensities :
0.050000 0.020000 0.030000 0.010000 0.060000 0.010000 0.020000
Not matched SDF shifts :
114.10 116.20 131.10

Rank: 66
ID : 3439

Name : LTS0272370
CAS : nan
MW : 245.23
Score : 0.7 (7/10 carbons)
Cumulated absolute difference : 2.04
Spectrum shifts left : 40/47

Matched spectrum shifts :
62.64 64.35 74.30 75.71 78.19 78.46 103.43
Matched SDF shifts :
62.80 64.40 74.20 75.20 78.00 78.20 104.20
Matched spectrum intensities :
0.050000 0.020000 0.020000 0.010000 0.060000 0.010000 0.020000
Not matched SDF shifts :
114.10 116.20 131.10

Rank: 67
ID : 504
Name : LTS0081012
CAS : nan
MW : 296.23
Score : 0.7 (7/10 carbons)
Cumulated absolute difference : 2.89
Spectrum shifts left : 40/47

Matched spectrum shifts :
61.91 71.32 74.51 76.25 77.22 78.61 103.43
Matched SDF shifts :
61.10 71.40 75.10 76.30 77.70 78.60 104.30
Matched spectrum intensities :

0.010000 0.050000 0.020000 0.030000 0.010000 0.010000 0.020000
Not matched SDF shifts :
32.70 173.60 174.80
