# Supplementary material for: Inhibition of Triacylglycerol Accumulation and Oxidized Hydroperoxides in Hepatocytes by Allium cepa (Bulb)
Source: Antioxidants (Basel). 2025 May 29;14(6):653. doi: 10.3390/antiox14060653 (PMC12190010; doi:10.3390/antiox14060653)
Supplement: Supplementary file 1 [file antioxidants-14-00653-s001.zip › File S1-S3/File S2- Supporting information for comparison of inhibited TG(OOH)n lipid species-end.pdf]

Supporting Information (SI-2)

Inhibition of triacylglycerol accumulation and oxidized hydroperoxides in hepatocytes by *Allium cepa* (bulb)

Dya Fita Dibwe [1], Saki Oba [2], Satomi Monde [2], and Shu-Ping Hui [1,*]

[1] Faculty of Health Sciences, Hokkaido University, Kita-12, Nishi-5, Kita-Ku, Sapporo 060-0812, Japan;
[2] Graduate School of Health Sciences, Hokkaido University, Kita-12, Nishi-5, Kita-Ku, Sapporo 060-0812, Japan;

* Correspondences: keino@hs.hokudai.ac.jp; Tel./Fax: +81-11-706-36

Supporting Information

Table of contents:

1. **Figure SI-2. 1.** Comparison of inhibited TG(OOH)$_n$ lipid species in OA and LA by AL6 and their molecular lipid species with C18: 1 and C18:2 P3
2. **Figure SI-2. 2.** Comparison of inhibited TGOOH lipid species in OA and LA by AL6 and their molecular lipid species with C18: 1 and C18:2 P4
3. **Figure SI-3. 3**. Comparison of inhibited TG(OOH)$_3$ lipid species in OA and LA by AL6 and their molecular lipid species with C18: 1 and C18:2 P5
4. **Table SI-2. 1-(OA).** Lipid species with their acyl chains of and the mass TAG and TG(OOH)n in OA condition P6
5. **Table SI-2. 2-(OA).** Common Name of Lipid species TAG from OA condition with their number of isomeres (Lipid maps) P8
6. **Table SI-2. 3-(OA).** Smile of Lipid species TAG from OA condition (Lipid maps) P9
7. **Table SI-2. 4-(LA)** Lipid species with their acyl chains of and the mass TAG and TG(OOH)n in LA condition P13
8. **Table SI-2. 5-(LA).** Common Name of Lipid species TAG from LA condition with their number of isomeres (Lipid maps) P23
9.
10. **Table SI-2. 6-(LA).** Smile of Lipid species TAG from LA condition (Lipid maps) P28

Table SI-2. 1-(OA) Lipid species with their acyl chains of and the mass TAG and TG(OOH)n in OA condition

LM ID	Formula	Sum Composition	Abbrev Chains	Exact Mass	TG (M+NH4)	TGOOH (M+NH4)	TG(OOH)2 (M+NH4)	TG(OOH)3 (M+NH4)
				TGOOH				
TG 54:9								
LMGL03013034	C57H92O6	TG 54:9	TG 18:1_18:4_18:4	872.68939	890.72321	922.713013	954.702813	986.692613
LMGL03016901	C57H92O6	TG 54:9	TG 18:4_18:1_18:4	872.68939	890.72321	922.713013	954.702813	986.692613
TG 54:6								
LMGL03010373	C57H98O6	TG 54:6	TG 18:1_18:2_18:3	878.73634	896.77016	928.759963	960.749763	992.739563
LMGL03010400	C57H98O6	TG 54:6	TG 16:1_18:1_20:4	878.73634	896.77016	928.759963	960.749763	992.739563
LMGL03010403	C57H98O6	TG 54:6	TG 16:0_18:1_20:5	878.73634	896.77016	928.759963	960.749763	992.739563
LMGL03013030	C57H98O6	TG 54:6	TG 18:1_18:1_18:4	878.73634	896.77016	928.759963	960.749763	992.739563
LMGL03014380	C57H98O6	TG 54:6	TG 14:0_18:1_22:5	878.73634	896.77016	928.759963	960.749763	992.739563
LMGL03014785	C57H98O6	TG 54:6	TG 14:1_18:1_22:4	878.73634	896.77016	928.759963	960.749763	992.739563
LMGL03016217	C57H98O6	TG 54:6	TG 18:1_18:2_18:3	878.73634	896.77016	928.759963	960.749763	992.739563
TG 56:10								
LMGL03016249	C59H94O6	TG 56:10	TG 18:1_18:4_20:5	898.70504	916.73886	948.728663	980.718463	1012.70826

LM ID	Formula	Sum Composition	Abbrev Chains	Exact Mass	TG (M+NH4)	TGOOH (M+NH4)	TG(OOH)2 (M+NH4)	TG(OOH)3 (M+NH4)
				TG(OOH)2				
TG 48:5								
LMGL03013465	C51H88O6	TG 48:5	TG 12:0_18:1_18:4	796.65809	814.69191	846.681713	878.671513	910.661313
TG 50:2								
LMGL03010043	C53H98O6	TG 50:2	TG 16:0_16:1_18:1	830.73634	848.77016	880.759963	912.749763	944.739563
LMGL03012792	C53H98O6	TG 50:2	TG 14:0_18:1_18:1	830.73634	848.77016	880.759963	912.749763	944.739563
LMGL03013469	C53H98O6	TG 50:2	TG 12:0_18:1_20:1	830.73634	848.77016	880.759963	912.749763	944.739563
LMGL03013932	C53H98O6	TG 50:2	TG 13:0_18:1_19:1	830.73634	848.77016	880.759963	912.749763	944.739563
LMGL03014747	C53H98O6	TG 50:2	TG 14:1_18:0_18:1	830.73634	848.77016	880.759963	912.749763	944.739563
LMGL03015082	C53H98O6	TG 50:2	TG 15:0_17:1_18:1	830.73634	848.77016	880.759963	912.749763	944.739563
LMGL03015410	C53H98O6	TG 50:2	TG 15:1_17:0_18:1	830.73634	848.77016	880.759963	912.749763	944.739563

LM ID	Formula	Sum Composition	Abbrev Chains	Exact Mass	TG (M+NH4)	TGOOH (M+NH4)	TG(OOH)2 (M+NH4)	TG(OOH)3 (M+NH4)
				TG(OOH)3				
TG 52:5								
LMGL03010140	C55H96O6	TG 52:5	TG 17:2_17:2_18:1	852.72069	870.75451	902.744313	934.734113	966.723913
LMGL03010168	C55H96O6	TG 52:5	TG 16:1_18:1_18:3	852.72069	870.75451	902.744313	934.734113	966.723913
LMGL03013479	C55H96O6	TG 52:5	TG 12:0_18:1_22:4	852.72069	870.75451	902.744313	934.734113	966.723913
LMGL03014372	C55H96O6	TG 52:5	TG 14:0_18:1_20:4	852.72069	870.75451	902.744313	934.734113	966.723913
LMGL03014777	C55H96O6	TG 52:5	TG 14:1_18:1_20:3	852.72069	870.75451	902.744313	934.734113	966.723913
LMGL03015728	C55H96O6	TG 52:5	TG 16:0_18:1_18:4	852.72069	870.75451	902.744313	934.734113	966.723913
LMGL03015818	C55H96O6	TG 52:5	TG 16:1_18:1_18:3	852.72069	870.75451	902.744313	934.734113	966.723913

Table SI-2. 2-(OA) Common Name of Lipid species TG from OA condition with their number of isomeres (Lipid maps)

**LM ID**	**Common Name**
**TGOOH**	
TAG 54:9	
LMGL03013034	TG(18:1(9Z)/18:4(6Z,9Z,12Z,15Z)/18:4(6Z,9Z,12Z,15Z))[iso3]
LMGL03016901	TG(18:2(12Y,14Y)/18:1(9Z)/18:2(12Y,14Y))
TAG 54:6	
LMGL03010373	TG(18:1(9Z)/18:2(9Z,12Z)/18:3(9Z,12Z,15Z))[iso6]
LMGL03010400	TG(16:1(9Z)/18:1(9Z)/20:4(5Z,8Z,11Z,14Z))[iso6]
LMGL03010403	TG(16:0/18:1(9Z)/20:5(5Z,8Z,11Z,14Z,17Z))[iso6]
LMGL03013030	TG(18:1(9Z)/18:1(9Z)/18:4(6Z,9Z,12Z,15Z))[iso3]
LMGL03014380	TG(14:0/18:1(9Z)/22:5(7Z,10Z,13Z,16Z,19Z))[iso6]
LMGL03014785	TG(14:1(9Z)/18:1(9Z)/22:4(7Z,10Z,13Z,16Z))[iso6]
LMGL03016217	TG(18:1(9Z)/18:2(9Z,12Z)/18:3(6Z,9Z,12Z))[iso6]
TAG 56:10	
LMGL03016249	TG(18:1(9Z)/18:4(6Z,9Z,12Z,15Z)/20:5(5Z,8Z,11Z,14Z,17Z))[iso6]

LM ID	Common Name
**TG(OOH)2**	
TAG 48:5	
LMGL03013465	TG(12:0/18:1(9Z)/18:4(6Z,9Z,12Z,15Z))[iso6]
TAG 50:2	
LMGL03010043	TG(16:0/16:1(9Z)/18:1(9Z))[iso6]
LMGL03012792	TG(14:0/18:1(9Z)/18:1(9Z))[iso3]
LMGL03013469	TG(12:0/18:1(9Z)/20:1(11Z))[iso6]
LMGL03013932	TG(13:0/18:1(9Z)/19:1(9Z))[iso6]
LMGL03014747	TG(14:1(9Z)/18:0/18:1(9Z))[iso6]
LMGL03015082	TG(15:0/17:1(9Z)/18:1(9Z))[iso6]
LMGL03015410	TG(15:1(9Z)/17:0/18:1(9Z))[iso6]

LM ID	Common Name
**TG(OOH)3**	
TAG 52:5	
LMGL03010140	TG(17:2(9Z,12Z)/17:2(9Z,12Z)/18:1(9Z))[iso3]
LMGL03010168	TG(16:1(9Z)/18:1(9Z)/18:3(9Z,12Z,15Z))[iso6]
LMGL03013479	TG(12:0/18:1(9Z)/22:4(7Z,10Z,13Z,16Z))[iso6]
LMGL03014372	TG(14:0/18:1(9Z)/20:4(5Z,8Z,11Z,14Z))[iso6]
LMGL03014777	TG(14:1(9Z)/18:1(9Z)/20:3(8Z,11Z,14Z))[iso6]
LMGL03015728	TG(16:0/18:1(9Z)/18:4(6Z,9Z,12Z,15Z))[iso6]
LMGL03015818	TG(16:1(9Z)/18:1(9Z)/18:3(6Z,9Z,12Z))[iso6]

Table SI3-3-(OA). Smile of Lipid species TAG from OA condition (Lipid maps)

LM ID	Common Name
	**SMILES**
TAG 54:9	
LMGL03013034	C(OC(=O)CCCC/C=C\C/C=C\C/C=C\C/C=C\CC)[C@]([H])(OC(CCCC/C=C\C/C=C\C/C=C\C/C=C\CC)=O)COC(CCCCCCC/C=C\CCCCCCCC)=O
LMGL03016901	C(OC(=O)CCCCCCCCCCC#CC#CCCC)[C@]([H])(OC(CCCCCCCCCCC#CC#CCCC)=O)COC(CCCCCCC/C=C\CCCCCCCC)=O
TAG 54:6	
LMGL03010373	C(OC(=O)CCCCCCC/C=C\C/C=C\C/C=C\CC)[C@]([H])(OC(CCCCCCC/C=C\C/C=C\CCCCC)=O)COC(CCCCCCC/C=C\CCCCCCC)=O
LMGL03010400	C(OC(=O)CCC/C=C\C/C=C\C/C=C\C/C=C\CCCCC)[C@]([H])(OC(CCCCCCC/C=C\CCCCCCCC)=O)COC(CCCCCCC/C=C\CCCCCC)=O
LMGL03010403	C(OC(=O)CCC/C=C\C/C=C\C/C=C\C/C=C\C/C=C\CC)[C@]([H])(OC(CCCCCCC/C=C\CCCCCCCC)=O)COC(CCCCCCCCCCCCCCC)=O
LMGL03013030	C(OC(=O)CCCC/C=C\C/C=C\C/C=C\C/C=C\CC)[C@]([H])(OC(CCCCCCC/C=C\CCCCCCCC)=O)COC(CCCCCCC/C=C\CCCCCCCC)=O
LMGL03014380	C(OC(=O)CCCCC/C=C\C/C=C\C/C=C\C/C=C\C/C=C\CC)[C@]([H])(OC(CCCCCCC/C=C\CCCCCCCC)=O)COC(CCCCCCCCCCCCC)=O
LMGL03014785	C(OC(=O)CCCCC/C=C\C/C=C\C/C=C\C/C=C\CCCCC)[C@]([H])(OC(CCCCCCC/C=C\CCCCCCCC)=O)COC(CCCCCCC/C=C\CCCC)=O
LMGL03016217	C(OC(=O)CCCC/C=C\C/C=C\C/C=C\CCCCC)[C@]([H])(OC(CCCCCCC/C=C\C/C=C\CCCCC)=O)COC(CCCCCCC/C=C\CCCCCCCC)=O
TAG 56:10	
LMGL03016249	C(OC(=O)CCC/C=C\C/C=C\C/C=C\C/C=C\C/C=C\CC)[C@]([H])(OC(CCCC/C=C\C/C=C\C/C=C\C/C=C\CC)=O)COC(CCCCCCC/C=C\CCCCCCCC)=O

LM ID	SMILES
TAG 48:5	
LMGL03013465	C(OC(=O)CCCC/C=C\C/C=C\C/C=C\C/C=C\CC)[C@]([H])(OC(CCCCCCC/C=C\CCCCCCCC)=O)COC(CCCCCCCCCCC)=O
TAG 50:2	
LMGL03010043	C(OC(=O)CCCCCCC/C=C\CCCCCCCC)[C@]([H])(OC(CCCCCCC/C=C\CCCCCC)=O)COC(CCCCCCCCCCCCCCC)=O
LMGL03012792	C(OC(=O)CCCCCCC/C=C\CCCCCCCC)[C@]([H])(OC(CCCCCCC/C=C\CCCCCCCC)=O)COC(CCCCCCCCCCCCC)=O
LMGL03013469	C(OC(=O)CCCCCCCCC/C=C\CCCCCCCC)[C@]([H])(OC(CCCCCCC/C=C\CCCCCCCC)=O)COC(CCCCCCCCCCC)=O
LMGL03013932	C(OC(=O)CCCCCCC/C=C\CCCCCCCCC)[C@]([H])(OC(CCCCCCC/C=C\CCCCCCCC)=O)COC(CCCCCCCCCCCC)=O
LMGL03014747	C(OC(=O)CCCCCCC/C=C\CCCCCCCC)[C@]([H])(OC(CCCCCCCCCCCCCCCCC)=O)COC(CCCCCCC/C=C\CCCC)=O
LMGL03015082	C(OC(=O)CCCCCCC/C=C\CCCCCCCC)[C@]([H])(OC(CCCCCCC/C=C\CCCCCCC)=O)COC(CCCCCCCCCCCCCC)=O
LMGL03015410	C(OC(=O)CCCCCCC/C=C\CCCCCCCC)[C@]([H])(OC(CCCCCCCCCCCCCCCC)=O)COC(CCCCCCC/C=C\CCCCC)=O

LM ID	SMILES
TAG 52:5	
LMGL03010140	C(OC(=O)CCCCCCC/C=C\CCCCCCCC)[C@]([H])(OC(CCCCCCC/C=C\C/C=C\CCCC)=O)COC(CCCCCCC/C=C\C/C=C\CCCC)=O
LMGL03010168	C(OC(=O)CCCCCCC/C=C\C/C=C\C/C=C\CC)[C@]([H])(OC(CCCCCCC/C=C\CCCCCCCC)=O)COC(CCCCCCC/C=C\CCCCCC)=O
LMGL03013479	C(OC(=O)CCCCC/C=C\C/C=C\C/C=C\C/C=C\CCCCC)[C@]([H])(OC(CCCCCCC/C=C\CCCCCCCC)=O)COC(CCCCCCCCCCC)=O

LMGL03014372	C(OC(=O)CCC/C=C\C/C=C\C/C=C\C/C=C\CCCCC)[C@]([H])(OC(CCCCCCC/C=C\CCCCCCCC)=O)COC(CCCCCCCCCCCCC)=O
LMGL03014777	C(OC(=O)CCCCCC/C=C\C/C=C\C/C=C\CCCCC)[C@]([H])(OC(CCCCCCC/C=C\CCCCCCCC)=O)COC(CCCCCCC/C=C\CCCC)=O
LMGL03015728	C(OC(=O)CCCC/C=C\C/C=C\C/C=C\C/C=C\CC)[C@]([H])(OC(CCCCCCC/C=C\CCCCCCCC)=O)COC(CCCCCCCCCCCCCCC)=O
LMGL03015818	C(OC(=O)CCCC/C=C\C/C=C\C/C=C\CCCCC)[C@]([H])(OC(CCCCCCC/C=C\CCCCCCCC)=O)COC(CCCCCCC/C=C\CCCCCC)=O

Table SI-2. 4-(LA) Lipid species with their acyl chains of and the mass TAG and TG(OOH)n in LA condition

LM ID	Formula	Sum Composition	Abbrev Chains	Exact Mass	M+H	M+NH4	TGOOH (M+NH4)	TG(OOH) 2 (M+NH4)	TG(OOH) 3 (M+NH4)
TGOOH									
TAG 54:9							31.99	63.9796	95.9694
**LMGL03014806**	C57H92O6	TG 54:9	TG 14:1_18:2_22:6	872.69	873.697	895.68	927.67	959.659	991.649
**LMGL03016289**	C57H92O6	TG 54:9	TG 18:2_18:3_18:4	872.69	873.697	895.68	927.67	959.659	991.649
**LMGL03016306**	C57H92O6	TG 54:9	TG 18:2_18:3_18:4	872.69	873.697	895.68	927.67	959.659	991.649
TAG 56:7							31.99	63.9796	95.9694
**LMGL03010734**	C59H100O6	TG 56:7	TG 18:2_18:2_20:3	904.75	905.76	927.74	959.73	991.721	1023.71
**LMGL03010741**	C59H100O6	TG 56:7	TG 18:1_18:2_20:4	904.75	905.76	927.74	959.73	991.721	1023.71
**LMGL03010747**	C59H100O6	TG 56:7	TG 18:0_18:2_20:5	904.75	905.76	927.74	959.73	991.721	1023.71
**LMGL03010917**	C59H100O6	TG 56:7	TG 16:1_18:2_22:4	904.75	905.76	927.74	959.73	991.721	1023.71
**LMGL03010921**	C59H100O6	TG 56:7	TG 16:0_18:2_22:5	904.75	905.76	927.74	959.73	991.721	1023.71
**LMGL03016294**	C59H100O6	TG 56:7	TG 18:2_18:3_20:2	904.75	905.76	927.74	959.73	991.721	1023.71
**LMGL03016312**	C59H100O6	TG 56:7	TG 18:2_18:4_20:1	904.75	905.76	927.74	959.73	991.721	1023.71
TG 58:10							31.99	63.9796	95.9694
**LMGL03011316**	C61H98O6	TG 58:10	TG 18:2_20:4_20:4	926.74	927.744	949.73	981.72	1013.71	1045.7
**LMGL03011322**	C61H98O6	TG 58:10	TG 18:2_20:3_20:5	926.74	927.744	949.73	981.72	1013.71	1045.7
**LMGL03011536**	C61H98O6	TG 58:10	TG 18:2_18:3_22:5	926.74	927.744	949.73	981.72	1013.71	1045.7

**LMGL03011544**	C61H98O6	TG 58:10	TG 18:2_18:2_22:6	926.74	927.744	949.73	981.72	1013.71	1045.7
**LMGL03016304**	C61H98O6	TG 58:10	TG 18:2_18:3_22:5	926.74	927.744	949.73	981.72	1013.71	1045.7
**LMGL03016322**	C61H98O6	TG 58:10	TG 18:2_18:4_22:4	926.74	927.744	949.73	981.72	1013.71	1045.7
TG 58:11							31.99	63.9796	95.9694
LMGL03011398	C61H96O6	TG 58:11	TG 18:2_20:4_20:5	924.72	925.729	947.71	979.7	1011.69	1043.68
LMGL03011620	C61H96O6	TG 58:11	TG 18:2_18:3_22:6	924.72	925.729	947.71	979.7	1011.69	1043.68
LMGL03016305	C61H96O6	TG 58:11	TG 18:2_18:3_22:6	924.72	925.729	947.71	979.7	1011.69	1043.68
LMGL03016323	C61H96O6	TG 58:11	TG 18:2_18:4_22:5	924.72	925.729	947.71	979.7	1011.69	1043.68
							31.99	63.9796	95.9694
**LM ID**	**Formula**	**Sum Composition**	**Abbrev Chains**	**Exact Mass**	**M+H**	**M+Na**	######	#VALUE!	#VALUE!
TAG(OOH)2									
TAG 54:3									
LMGL03010252	C57H104O6	TG 54:3	TG 18:0_18:1_18:2	884.78	885.791	907.77	939.76	971.753	1003.74
LMGL03010263	C57H104O6	TG 54:3	TG 16:1_18:2_20:0	884.78	885.791	907.77	939.76	971.753	1003.74
LMGL03010267	C57H104O6	TG 54:3	TG 16:0_18:2_20:1	884.78	885.791	907.77	939.76	971.753	1003.74
LMGL03010294	C57H104O6	TG 54:3	TG 17:1_18:2_19:0	884.78	885.791	907.77	939.76	971.753	1003.74
LMGL03014395	C57H104O6	TG 54:3	TG 14:0_18:2_22:1	884.78	885.791	907.77	939.76	971.753	1003.74
LMGL03014800	C57H104O6	TG 54:3	TG 14:1_18:2_22:0	884.78	885.791	907.77	939.76	971.753	1003.74
LMGL03015528	C57H104O6	TG 54:3	TG 15:1_18:2_21:0	884.78	885.791	907.77	939.76	971.753	1003.74
LMGL03015911	C57H104O6	TG 54:3	TG 17:0_18:2_19:1	884.78	885.791	907.77	939.76	971.753	1003.74
TAG 62:13							31.99	63.9796	95.9694
LMGL03012263	C65H100O6	TG 62:13	TG 20:5_20:5_22:3	976.75	977.76	999.74	1031.7	1063.72	1095.71

LMGL03012318	$C_{65}H_{100}O_6$	TG 62:13	TG 20:4_20:5_22:4	976.75	977.76	999.74	1031.7	1063.72	1095.71
LMGL03012326	$C_{65}H_{100}O_6$	TG 62:13	TG 20:3_20:5_22:5	976.75	977.76	999.74	1031.7	1063.72	1095.71
LMGL03012327	$C_{65}H_{100}O_6$	TG 62:13	TG 20:4_20:4_22:5	976.75	977.76	999.74	1031.7	1063.72	1095.71
LMGL03012336	$C_{65}H_{100}O_6$	TG 62:13	TG 20:2_20:5_22:6	976.75	977.76	999.74	1031.7	1063.72	1095.71
LMGL03012337	$C_{65}H_{100}O_6$	TG 62:13	TG 20:3_20:4_22:6	976.75	977.76	999.74	1031.7	1063.72	1095.71
LMGL03012391	$C_{65}H_{100}O_6$	TG 62:13	TG 18:3_22:5_22:5	976.75	977.76	999.74	1031.7	1063.72	1095.71
LMGL03012401	$C_{65}H_{100}O_6$	TG 62:13	TG 18:3_22:4_22:6	976.75	977.76	999.74	1031.7	1063.72	1095.71
LMGL03012399	$C_{65}H_{100}O_6$	TG 62:13	TG 18:1_22:6_22:6	976.75	977.76	999.74	1031.7	1063.72	1095.71
LMGL03013079	$C_{65}H_{100}O_6$	TG 62:13	TG 18:3_22:5_22:5	976.75	977.76	999.74	1031.7	1063.72	1095.71
LMGL03016506	$C_{65}H_{100}O_6$	TG 62:13	TG 18:3_22:4_22:6	976.75	977.76	999.74	1031.7	1063.72	1095.71
LMGL03016670	$C_{65}H_{100}O_6$	TG 62:13	TG 18:4_22:3_22:6	976.75	977.76	999.74	1031.7	1063.72	1095.71
LMGL03016671	$C_{65}H_{100}O_6$	TG 62:13	TG 18:4_22:4_22:5	976.75	977.76	999.74	1031.7	1063.72	1095.71
			**Not reported species in LM**				31.99	63.9796	95.9694
	$C_{65}H_{100}O_6$	TG 62:13	TG 18:2_22:6_22:5				31.99	63.9796	95.9694
	$C_{65}H_{100}O_6$	TG 62:13	TG 18:2_22:5_22:6				31.99	63.9796	95.9694
	$C_{65}H_{100}O_6$	TG 62:13	TG 18:2_22:5_22:6				31.99	63.9796	95.9694
	$C_{65}H_{100}O_6$	TG 62:13	TG 18:2_22:6_22:5				31.99	63.9796	95.9694
	$C_{65}H_{100}O_6$	TG 62:13	TG 18:2_22:5_22:6				31.99	63.9796	95.9694
							31.99	63.9796	95.9694

							31.99	63.9796	95.9694
**LM ID**	**Formula**	**Sum Composition**	**Abbrev Chains**	**Exact Mass**	**M+H**	**M+Na**	######	#VALUE!	#VALUE!
TG(OOH)3							31.99	63.9796	95.9694
TAG 50:3							31.99	63.9796	95.9694
LMGL03010053	C53H96O6	TG 50:3	TG 16:0_16:1_18:2	828.72	829.729	851.71	883.7	915.69	947.68
LMGL03013488	C53H96O6	TG 50:3	TG 12:0_18:2_20:1	828.72	829.729	851.71	883.7	915.69	947.68
LMGL03013951	C53H96O6	TG 50:3	TG 13:0_18:2_19:1	828.72	829.729	851.71	883.7	915.69	947.68
LMGL03014362	C53H96O6	TG 50:3	TG 14:0_18:1_18:2	828.72	829.729	851.71	883.7	915.69	947.68
LMGL03014748	C53H96O6	TG 50:3	TG 14:1_18:0_18:2	828.72	829.729	851.71	883.7	915.69	947.68
LMGL03015083	C53H96O6	TG 50:3	TG 15:0_17:1_18:2	828.72	829.729	851.71	883.7	915.69	947.68
LMGL03015411	C53H96O6	TG 50:3	TG 15:1_17:0_18:2	828.72	829.729	851.71	883.7	915.69	947.68
TAG 52:5							31.99	63.9796	95.9694
LMGL03010144	C55H96O6	TG 52:5	TG 17:1_17:2_18:2	852.72	853.729	875.71	907.7	939.69	971.68
LMGL03010163	C55H96O6	TG 52:5	TG 16:1_18:2_18:2	852.72	853.729	875.71	907.7	939.69	971.68
LMGL03010167	C55H96O6	TG 52:5	TG 16:0_18:2_18:3	852.72	853.729	875.71	907.7	939.69	971.68
LMGL03013497	C55H96O6	TG 52:5	TG 12:0_18:2_22:3	852.72	853.729	875.71	907.7	939.69	971.68
LMGL03014390	C55H96O6	TG 52:5	TG 14:0_18:2_20:3	852.72	853.729	875.71	907.7	939.69	971.68
LMGL03014795	C55H96O6	TG 52:5	TG 14:1_18:2_20:2	852.72	853.729	875.71	907.7	939.69	971.68
LMGL03015731	C55H96O6	TG 52:5	TG 16:0_18:2_18:3	852.72	853.729	875.71	907.7	939.69	971.68
TG 54:10							31.99	63.9796	95.9694
LMGL03012804	C57H90O6	TG 54:10	TG 14:0_20:5_20:5	870.67	871.682	893.66	925.65	957.643	989.633
LMGL03013046	C57H90O6	TG 54:10	TG 18:3_18:3_18:4	870.67	871.682	893.66	925.65	957.643	989.633
LMGL03013081	C57H90O6	TG 54:10	TG 18:3_18:3_18:4	870.67	871.682	893.66	925.65	957.643	989.633
LMGL03013635	C57H90O6	TG 54:10	TG 12:0_20:4_22:6	870.67	871.682	893.66	925.65	957.643	989.633
LMGL03013642	C57H90O6	TG 54:10	TG 12:0_20:5_22:5	870.67	871.682	893.66	925.65	957.643	989.633
LMGL03014451	C57H90O6	TG 54:10	TG 14:0_18:4_22:6	870.67	871.682	893.66	925.65	957.643	989.633
LMGL03014824	C57H90O6	TG 54:10	TG 14:1_18:3_22:6	870.67	871.682	893.66	925.65	957.643	989.633

LMGL03014841	C57H90O6	TG 54:10	TG 14:1_18:3_22:6	870.67	871.682	893.66	925.65	957.643	989.633
LMGL03014856	C57H90O6	TG 54:10	TG 14:1_18:4_22:5	870.67	871.682	893.66	925.65	957.643	989.633
LMGL03014933	C57H90O6	TG 54:10	TG 14:1_20:4_20:5	870.67	871.682	893.66	925.65	957.643	989.633
LMGL03015854	C57H90O6	TG 54:10	TG 16:1_18:4_20:5	870.67	871.682	893.66	925.65	957.643	989.633
LMGL03016355	C57H90O6	TG 54:10	TG 18:3_18:3_18:4	870.67	871.682	893.66	925.65	957.643	989.633
		TG 54:10	TG 18:2_18:4_18:4				31.99	63.9796	95.9694
							31.99	63.9796	95.9694
							31.99	63.9796	95.9694
TG 54:4							31.99	63.9796	95.9694
LMGL03010287	C57H102O6	TG 54:4	TG 18:0_18:2_18:2	882.77	883.775	905.76	937.75	969.737	1001.73
LMGL03010288	C57H102O6	TG 54:4	TG 18:1_18:1_18:2	882.77	883.775	905.76	937.75	969.737	1001.73
LMGL03010303	C57H102O6	TG 54:4	TG 16:1_18:2_20:1	882.77	883.775	905.76	937.75	969.737	1001.73
LMGL03010307	C57H102O6	TG 54:4	TG 16:0_18:2_20:2	882.77	883.775	905.76	937.75	969.737	1001.73
LMGL03010333	C57H102O6	TG 54:4	TG 17:2_18:2_19:0	882.77	883.775	905.76	937.75	969.737	1001.73

LMGL03014396	C57H102O6	TG 54:4	TG 14:0_18:2_22:2	882.77	883.775	905.76	937.75	969.737	1001.73
LMGL03014801	C57H102O6	TG 54:4	TG 14:1_18:2_22:1	882.77	883.775	905.76	937.75	969.737	1001.73
LMGL03015994	C57H102O6	TG 54:4	TG 17:1_18:2_19:1	882.77	883.775	905.76	937.75	969.737	1001.73
							31.99	63.9796	95.9694
LMGL03010291	C57H102O6	TG 54:4	TG 18:0_18:1_18:3	882.77	883.775	905.76	937.75	969.737	1001.73
LMGL03013022	C57H102O6	TG 54:4	TG 18:0_18:0_18:4	882.77	883.775	905.76	937.75	969.737	1001.73
LMGL03016142	C57H102O6	TG 54:4	TG 18:0_18:1_18:3	882.77	883.775	905.76	937.75	969.737	1001.73
TAG 56:10							31.99	63.9796	95.9694
LMGL03010946	C59H94O6	TG 56:10	TG 18:3_18:3_20:4	898.71	899.713	921.69	953.68	985.674	1017.66
LMGL03010947	C59H94O6	TG 56:10	TG 16:0_20:5_20:5	898.71	899.713	921.69	953.68	985.674	1017.66
LMGL03010948	C59H94O6	TG 56:10	TG 16:1_20:4_20:5	898.71	899.713	921.69	953.68	985.674	1017.66
LMGL03011078	C59H94O6	TG 56:10	TG 17:2_17:2_22:6	898.71	899.713	921.69	953.68	985.674	1017.66
LMGL03011152	C59H94O6	TG 56:10	TG 16:1_18:3_22:6	898.71	899.713	921.69	953.68	985.674	1017.66

LMGL03012689	C59H94O6	TG 56:10	TG 12:0_22:5_22:5	898.71	899.713	921.69	953.68	985.674	1017.66
LMGL03013053	C59H94O6	TG 56:10	TG 18:3_18:3_20:4	898.71	899.713	921.69	953.68	985.674	1017.66
LMGL03013091	C59H94O6	TG 56:10	TG 18:4_18:4_20:2	898.71	899.713	921.69	953.68	985.674	1017.66
LMGL03013670	C59H94O6	TG 56:10	TG 12:0_22:4_22:6	898.71	899.713	921.69	953.68	985.674	1017.66
LMGL03014535	C59H94O6	TG 56:10	TG 14:0_20:4_22:6	898.71	899.713	921.69	953.68	985.674	1017.66
LMGL03014542	C59H94O6	TG 56:10	TG 14:0_20:5_22:5	898.71	899.713	921.69	953.68	985.674	1017.66
LMGL03014932	C59H94O6	TG 56:10	TG 14:1_20:3_22:6	898.71	899.713	921.69	953.68	985.674	1017.66
LMGL03014940	C59H94O6	TG 56:10	TG 14:1_20:4_22:5	898.71	899.713	921.69	953.68	985.674	1017.66
LMGL03014947	C59H94O6	TG 56:10	TG 14:1_20:5_22:4	898.71	899.713	921.69	953.68	985.674	1017.66
LMGL03015771	C59H94O6	TG 56:10	TG 16:0_18:4_22:6	898.71	899.713	921.69	953.68	985.674	1017.66
LMGL03015843	C59H94O6	TG 56:10	TG 16:1_18:3_22:6	898.71	899.713	921.69	953.68	985.674	1017.66
LMGL03015861	C59H94O6	TG 56:10	TG 16:1_18:4_22:5	898.71	899.713	921.69	953.68	985.674	1017.66

LMGL03016249	C59H94O6	TG 56:10	TG 18:1_18:4_20:5	898.71	899.713	921.69	953.68	985.674	1017.66
LMGL03016362	C59H94O6	TG 56:10	TG 18:3_18:3_20:4	898.71	899.713	921.69	953.68	985.674	1017.66
LMGL03016377	C59H94O6	TG 56:10	TG 18:3_18:4_20:3	898.71	899.713	921.69	953.68	985.674	1017.66
LMGL03016513	C59H94O6	TG 56:10	TG 18:3_18:4_20:3	898.71	899.713	921.69	953.68	985.674	1017.66
		TG 56:10	TG 18:2_18:4_20:4				31.99	63.9796	95.9694
		TG 56:10	TG 18:1_18:4_20:5				31.99	63.9796	95.9694
		TG 56:10	TG 18:2_18:4_20:4				31.99	63.9796	95.9694
		TG 56:10	TG 18:2_18:5_20:3				31.99	63.9796	95.9694
		TG 56:10	TG 18:2_18:4_20:4				31.99	63.9796	95.9694
**LMGL0301D7GQ**	-	**TG(56:10); TG(18:2_18:4_20:4)**	-	-	-	-	######	#VALUE!	#VALUE!
TG 58:7							31.99	63.9796	95.9694
LMGL03011298	C61H104O6	TG 58:7	TG 18:1_18:2_22:4	932.78	933.791	955.77	987.76	1019.75	1051.74
LMGL03011304	C61H104O6	TG 58:7	TG 18:0_18:2_22:5	932.78	933.791	955.77	987.76	1019.75	1051.74
TG 60:11							31.99	63.9796	95.9694
LMGL03011769	C63H100O6	TG 60:11	TG 20:3_20:4_20:4	952.75	953.76	975.74	1007.7	1039.72	1071.71
LMGL03011770	C63H100O6	TG 60:11	TG 20:1_20:5_20:5	952.75	953.76	975.74	1007.7	1039.72	1071.71

LMGL03011772	C63H100O6	TG 60:11	TG 20:3_20:3_20:5	952.75	953.76	975.74	1007.7	1039.72	1071.71
LMGL03011771	C63H100O6	TG 60:11	TG 20:2_20:4_20:5	952.75	953.76	975.74	1007.7	1039.72	1071.71
LMGL03011863	C63H100O6	TG 60:11	TG 18:3_20:5_22:3	952.75	953.76	975.74	1007.7	1039.72	1071.71
LMGL03011949	C63H100O6	TG 60:11	TG 18:3_20:4_22:4	952.75	953.76	975.74	1007.7	1039.72	1071.71
LMGL03011959	C63H100O6	TG 60:11	TG 18:1_20:5_22:5	952.75	953.76	975.74	1007.7	1039.72	1071.71
LMGL03011961	C63H100O6	TG 60:11	TG 18:3_20:3_22:5	952.75	953.76	975.74	1007.7	1039.72	1071.71
LMGL03011969	C63H100O6	TG 60:11	TG 18:0_20:5_22:6	952.75	953.76	975.74	1007.7	1039.72	1071.71
LMGL03011970	C63H100O6	TG 60:11	TG 18:1_20:4_22:6	952.75	953.76	975.74	1007.7	1039.72	1071.71
LMGL03011972	C63H100O6	TG 60:11	TG 18:3_20:2_22:6	952.75	953.76	975.74	1007.7	1039.72	1071.71
LMGL03012075	C63H100O6	TG 60:11	TG 16:1_22:5_22:5	952.75	953.76	975.74	1007.7	1039.72	1071.71
LMGL03012086	C63H100O6	TG 60:11	TG 16:0_22:5_22:6	952.75	953.76	975.74	1007.7	1039.72	1071.71
LMGL03012087	C63H100O6	TG 60:11	TG 16:1_22:4_22:6	952.75	953.76	975.74	1007.7	1039.72	1071.71

LMGL03016452	C63H100O6	TG 60:11	TG 18:3_20:2_22:6	952.75	953.76	975.74	1007.7	1039.72	1071.71
LMGL03016461	C63H100O6	TG 60:11	TG 18:3_20:3_22:5	952.75	953.76	975.74	1007.7	1039.72	1071.71
LMGL03016469	C63H100O6	TG 60:11	TG 18:3_20:4_22:4	952.75	953.76	975.74	1007.7	1039.72	1071.71
LMGL03016476	C63H100O6	TG 60:11	TG 18:3_20:5_22:3	952.75	953.76	975.74	1007.7	1039.72	1071.71
LMGL03016626	C63H100O6	TG 60:11	TG 18:4_20:3_22:4	952.75	953.76	975.74	1007.7	1039.72	1071.71
LMGL03016617	C63H100O6	TG 60:11	TG 18:4_20:2_22:5	952.75	953.76	975.74	1007.7	1039.72	1071.71
LMGL03016607	C63H100O6	TG 60:11	TG 18:4_20:1_22:6	952.75	953.76	975.74	1007.7	1039.72	1071.71
LMGL03016634	C63H100O6	TG 60:11	TG 18:4_20:4_22:3	952.75	953.76	975.74	1007.7	1039.72	1071.71
LMGL03016641	C63H100O6	TG 60:11	TG 18:4_20:5_22:2	952.75	953.76	975.74	1007.7	1039.72	1071.71
TAG 60:11							31.99	63.9796	95.9694
		TG 60:11	TG 18:2_20:5_22:4				31.99	63.9796	95.9694
		TG 60:11	TG 18:2_20:4_22:5	LMISSD: Exact Structure Results for TG 18:2_20:4_22:5			31.99	63.9796	95.9694
		TG 60:11	TG 18:2_20:3_22:6	LMISSD: Exact Structure Results for TG 18:2_20:3_22:6			31.99	63.9796	95.9694
		TG 60:11	TG 18:2_20:3_22:6	LMISSD: Exact Structure Results for TG 18:2_20:3_22:6			31.99	63.9796	95.9694
							31.99	63.9796	95.9694

Table SI-2. 5-(LA) Common Name of Lipid species TAG from LA condition with their number of isomeres (Lipid maps)

LM ID	Common Name
TGOOH	
TAG 54:9	
**LMGL03014806**	TG(14:1(9Z)/18:2(9Z,12Z)/22:6(4Z,7Z,10Z,13Z,16Z,19Z))[iso6]
**LMGL03016289**	TG(18:2(9Z,12Z)/18:3(6Z,9Z,12Z)/18:4(6Z,9Z,12Z,15Z))[iso6]
**LMGL03016306**	TG(18:2(9Z,12Z)/18:3(9Z,12Z,15Z)/18:4(6Z,9Z,12Z,15Z))[iso6]
TAG 56:7	
**LMGL03010734**	TG(18:2(9Z,12Z)/18:2(9Z,12Z)/20:3(8Z,11Z,14Z))[iso3]
**LMGL03010741**	TG(18:1(9Z)/18:2(9Z,12Z)/20:4(5Z,8Z,11Z,14Z))[iso6]
**LMGL03010747**	TG(18:0/18:2(9Z,12Z)/20:5(5Z,8Z,11Z,14Z,17Z))[iso6]
**LMGL03010917**	TG(16:1(9Z)/18:2(9Z,12Z)/22:4(7Z,10Z,13Z,16Z))[iso6]
**LMGL03010921**	TG(16:0/18:2(9Z,12Z)/22:5(7Z,10Z,13Z,16Z,19Z))[iso6]
**LMGL03016294**	TG(18:2(9Z,12Z)/18:3(6Z,9Z,12Z)/20:2(11Z,14Z))[iso6]
**LMGL03016312**	TG(18:2(9Z,12Z)/18:4(6Z,9Z,12Z,15Z)/20:1(11Z))[iso6]
TAG 58:10	
**LMGL03011316**	TG(18:2(9Z,12Z)/20:4(5Z,8Z,11Z,14Z)/20:4(5Z,8Z,11Z,14Z))[iso3]
**LMGL03011322**	TG(18:2(9Z,12Z)/20:3(8Z,11Z,14Z)/20:5(5Z,8Z,11Z,14Z,17Z))[iso6]
**LMGL03011536**	TG(18:2(9Z,12Z)/18:3(9Z,12Z,15Z)/22:5(7Z,10Z,13Z,16Z,19Z))[iso6]
**LMGL03011544**	TG(18:2(9Z,12Z)/18:2(9Z,12Z)/22:6(4Z,7Z,10Z,13Z,16Z,19Z))[iso3]
**LMGL03016304**	TG(18:2(9Z,12Z)/18:3(6Z,9Z,12Z)/22:5(7Z,10Z,13Z,16Z,19Z))[iso6]
**LMGL03016322**	TG(18:2(9Z,12Z)/18:4(6Z,9Z,12Z,15Z)/22:4(7Z,10Z,13Z,16Z))[iso6]
TAG 58:11	
LMGL03011398	TG(18:2(9Z,12Z)/20:4(5Z,8Z,11Z,14Z)/20:5(5Z,8Z,11Z,14Z,17Z))[iso6]

LMGL03011620	TG(18:2(9Z,12Z)/18:3(9Z,12Z,15Z)/22:6(4Z,7Z,10Z,13Z,16Z,19Z))[iso6]
LMGL03016305	TG(18:2(9Z,12Z)/18:3(6Z,9Z,12Z)/22:6(4Z,7Z,10Z,13Z,16Z,19Z))[iso6]
LMGL03016323	TG(18:2(9Z,12Z)/18:4(6Z,9Z,12Z,15Z)/22:5(7Z,10Z,13Z,16Z,19Z))[iso6]

LM ID	Common Name
TG(OOH)2	
TG 54:3	
LMGL03010252	TG(18:0/18:1(9Z)/18:2(9Z,12Z))[iso6]
LMGL03010263	TG(16:1(9Z)/18:2(9Z,12Z)/20:0)[iso6]
LMGL03010267	TG(16:0/18:2(9Z,12Z)/20:1(11Z))[iso6]
LMGL03010294	TG(17:1(9Z)/18:2(9Z,12Z)/19:0)[iso6]
LMGL03014395	TG(14:0/18:2(9Z,12Z)/22:1(11Z))[iso6]
LMGL03014800	TG(14:1(9Z)/18:2(9Z,12Z)/22:0)[iso6]
LMGL03015528	TG(15:1(9Z)/18:2(9Z,12Z)/21:0)[iso6]
LMGL03015911	TG(17:0/18:2(9Z,12Z)/19:1(9Z))[iso6]
TG 62:13	
LMGL03012263	TG(20:5(5Z,8Z,11Z,14Z,17Z)/20:5(5Z,8Z,11Z,14Z,17Z)/22:3(10Z,13Z,16Z))[iso3]
LMGL03012318	TG(20:4(5Z,8Z,11Z,14Z)/20:5(5Z,8Z,11Z,14Z,17Z)/22:4(7Z,10Z,13Z,16Z))[iso6]
LMGL03012326	TG(20:3(8Z,11Z,14Z)/20:5(5Z,8Z,11Z,14Z,17Z)/22:5(7Z,10Z,13Z,16Z,19Z))[iso6]
LMGL03012327	TG(20:4(5Z,8Z,11Z,14Z)/20:4(5Z,8Z,11Z,14Z)/22:5(7Z,10Z,13Z,16Z,19Z))[iso3]
LMGL03012336	TG(20:2(11Z,14Z)/20:5(5Z,8Z,11Z,14Z,17Z)/22:6(4Z,7Z,10Z,13Z,16Z,19Z))[iso6]
LMGL03012337	TG(20:3(8Z,11Z,14Z)/20:4(5Z,8Z,11Z,14Z)/22:6(4Z,7Z,10Z,13Z,16Z,19Z))[iso6]
LMGL03012391	TG(18:3(9Z,12Z,15Z)/22:5(7Z,10Z,13Z,16Z,19Z)/22:5(7Z,10Z,13Z,16Z,19Z))[iso3]
LMGL03012401	TG(18:3(9Z,12Z,15Z)/22:4(7Z,10Z,13Z,16Z)/22:6(4Z,7Z,10Z,13Z,16Z,19Z))[iso6]
LMGL03012399	TG(18:1(9Z)/22:6(4Z,7Z,10Z,13Z,16Z,19Z)/22:6(4Z,7Z,10Z,13Z,16Z,19Z))[iso3]
LMGL03013079	TG(18:3(6Z,9Z,12Z)/22:5(7Z,10Z,13Z,16Z,19Z)/22:5(7Z,10Z,13Z,16Z,19Z))[iso3]
LMGL03016506	TG(18:3(6Z,9Z,12Z)/22:4(7Z,10Z,13Z,16Z)/22:6(4Z,7Z,10Z,13Z,16Z,19Z))[iso6]
LMGL03016670	TG(18:4(6Z,9Z,12Z,15Z)/22:3(10Z,13Z,16Z)/22:6(4Z,7Z,10Z,13Z,16Z,19Z))[iso6]

LMGL03016671	TG(18:4(6Z,9Z,12Z,15Z)/22:4(7Z,10Z,13Z,16Z)/22:5(7Z,10Z,13Z,16Z,19Z))[iso6]
**LM ID**	**Common Name**
TG(OOH)3	
TAG 50:3	
LMGL03010053	TG(16:0/16:1(9Z)/18:2(9Z,12Z))[iso6]
LMGL03013488	TG(12:0/18:2(9Z,12Z)/20:1(11Z))[iso6]
LMGL03013951	TG(13:0/18:2(9Z,12Z)/19:1(9Z))[iso6]
LMGL03014362	TG(14:0/18:1(9Z)/18:2(9Z,12Z))[iso6]
LMGL03014748	TG(14:1(9Z)/18:0/18:2(9Z,12Z))[iso6]
LMGL03015083	TG(15:0/17:1(9Z)/18:2(9Z,12Z))[iso6]
LMGL03015411	TG(15:1(9Z)/17:0/18:2(9Z,12Z))[iso6]
TG 52:5	
LMGL03010144	TG(17:1(9Z)/17:2(9Z,12Z)/18:2(9Z,12Z))[iso6]
LMGL03010163	TG(16:1(9Z)/18:2(9Z,12Z)/18:2(9Z,12Z))[iso3]
LMGL03010167	TG(16:0/18:2(9Z,12Z)/18:3(9Z,12Z,15Z))[iso6]
LMGL03013497	TG(12:0/18:2(9Z,12Z)/22:3(10Z,13Z,16Z))[iso6]
LMGL03014390	TG(14:0/18:2(9Z,12Z)/20:3(8Z,11Z,14Z))[iso6]
LMGL03014795	TG(14:1(9Z)/18:2(9Z,12Z)/20:2(11Z,14Z))[iso6]
LMGL03015731	TG(16:0/18:2(9Z,12Z)/18:3(6Z,9Z,12Z))[iso6]
TG 54:10	
LMGL03012804	TG(14:0/20:5(5Z,8Z,11Z,14Z,17Z)/20:5(5Z,8Z,11Z,14Z,17Z))[iso3]
LMGL03013046	TG(18:3(6Z,9Z,12Z)/18:3(6Z,9Z,12Z)/18:4(6Z,9Z,12Z,15Z))[iso3]
LMGL03013081	TG(18:3(9Z,12Z,15Z)/18:3(9Z,12Z,15Z)/18:4(6Z,9Z,12Z,15Z))[iso3]
LMGL03013635	TG(12:0/20:4(5Z,8Z,11Z,14Z)/22:6(4Z,7Z,10Z,13Z,16Z,19Z))[iso6]
LMGL03013642	TG(12:0/20:5(5Z,8Z,11Z,14Z,17Z)/22:5(7Z,10Z,13Z,16Z,19Z))[iso6]
LMGL03014451	TG(14:0/18:4(6Z,9Z,12Z,15Z)/22:6(4Z,7Z,10Z,13Z,16Z,19Z))[iso6]
LMGL03014824	TG(14:1(9Z)/18:3(6Z,9Z,12Z)/22:6(4Z,7Z,10Z,13Z,16Z,19Z))[iso6]
LMGL03014841	TG(14:1(9Z)/18:3(9Z,12Z,15Z)/22:6(4Z,7Z,10Z,13Z,16Z,19Z))[iso6]

LMGL03014856	TG(14:1(9Z)/18:4(6Z,9Z,12Z,15Z)/22:5(7Z,10Z,13Z,16Z,19Z))[iso6]
LMGL03014933	TG(14:1(9Z)/20:4(5Z,8Z,11Z,14Z)/20:5(5Z,8Z,11Z,14Z,17Z))[iso6]
LMGL03015854	TG(16:1(9Z)/18:4(6Z,9Z,12Z,15Z)/20:5(5Z,8Z,11Z,14Z,17Z))[iso6]
LMGL03016355	TG(18:3(6Z,9Z,12Z)/18:3(9Z,12Z,15Z)/18:4(6Z,9Z,12Z,15Z))[iso6]
TG 58:7	
LMGL03011298	TG(18:1(9Z)/18:2(9Z,12Z)/22:4(7Z,10Z,13Z,16Z))[iso6]
LMGL03011304	TG(18:0/18:2(9Z,12Z)/22:5(7Z,10Z,13Z,16Z,19Z))[iso6]
TG 60:11	
LMGL03011769	TG(20:3(8Z,11Z,14Z)/20:4(5Z,8Z,11Z,14Z)/20:4(5Z,8Z,11Z,14Z))[iso3]
LMGL03011770	TG(20:1(11Z)/20:5(5Z,8Z,11Z,14Z,17Z)/20:5(5Z,8Z,11Z,14Z,17Z))[iso3]
LMGL03011772	TG(20:3(8Z,11Z,14Z)/20:3(8Z,11Z,14Z)/20:5(5Z,8Z,11Z,14Z,17Z))[iso3]
LMGL03011771	TG(20:2(11Z,14Z)/20:4(5Z,8Z,11Z,14Z)/20:5(5Z,8Z,11Z,14Z,17Z))[iso6]
LMGL03011863	TG(18:3(9Z,12Z,15Z)/20:5(5Z,8Z,11Z,14Z,17Z)/22:3(10Z,13Z,16Z))[iso6]
LMGL03011949	TG(18:3(9Z,12Z,15Z)/20:4(5Z,8Z,11Z,14Z)/22:4(7Z,10Z,13Z,16Z))[iso6]
LMGL03011959	TG(18:1(9Z)/20:5(5Z,8Z,11Z,14Z,17Z)/22:5(7Z,10Z,13Z,16Z,19Z))[iso6]
LMGL03011961	TG(18:3(9Z,12Z,15Z)/20:3(8Z,11Z,14Z)/22:5(7Z,10Z,13Z,16Z,19Z))[iso6]
LMGL03011969	TG(18:0/20:5(5Z,8Z,11Z,14Z,17Z)/22:6(4Z,7Z,10Z,13Z,16Z,19Z))[iso6]
LMGL03011970	TG(18:1(9Z)/20:4(5Z,8Z,11Z,14Z)/22:6(4Z,7Z,10Z,13Z,16Z,19Z))[iso6]
LMGL03011972	TG(18:3(9Z,12Z,15Z)/20:2(11Z,14Z)/22:6(4Z,7Z,10Z,13Z,16Z,19Z))[iso6]
LMGL03012075	TG(16:1(9Z)/22:5(7Z,10Z,13Z,16Z,19Z)/22:5(7Z,10Z,13Z,16Z,19Z))[iso3]
LMGL03012086	TG(16:0/22:5(7Z,10Z,13Z,16Z,19Z)/22:6(4Z,7Z,10Z,13Z,16Z,19Z))[iso6]
LMGL03012087	TG(16:1(9Z)/22:4(7Z,10Z,13Z,16Z)/22:6(4Z,7Z,10Z,13Z,16Z,19Z))[iso6]
LMGL03016452	TG(18:3(6Z,9Z,12Z)/20:2(11Z,14Z)/22:6(4Z,7Z,10Z,13Z,16Z,19Z))[iso6]
LMGL03016461	TG(18:3(6Z,9Z,12Z)/20:3(8Z,11Z,14Z)/22:5(7Z,10Z,13Z,16Z,19Z))[iso6]
LMGL03016469	TG(18:3(6Z,9Z,12Z)/20:4(5Z,8Z,11Z,14Z)/22:4(7Z,10Z,13Z,16Z))[iso6]
LMGL03016476	TG(18:3(6Z,9Z,12Z)/20:5(5Z,8Z,11Z,14Z,17Z)/22:3(10Z,13Z,16Z))[iso6]
LMGL03016626	TG(18:4(6Z,9Z,12Z,15Z)/20:3(8Z,11Z,14Z)/22:4(7Z,10Z,13Z,16Z))[iso6]
LMGL03016617	TG(18:4(6Z,9Z,12Z,15Z)/20:2(11Z,14Z)/22:5(7Z,10Z,13Z,16Z,19Z))[iso6]

LMGL03016607	TG(18:4(6Z,9Z,12Z,15Z)/20:1(11Z)/22:6(4Z,7Z,10Z,13Z,16Z,19Z))[iso6]
LMGL03016634	TG(18:4(6Z,9Z,12Z,15Z)/20:4(5Z,8Z,11Z,14Z)/22:3(10Z,13Z,16Z))[iso6]
LMGL03016641	TG(18:4(6Z,9Z,12Z,15Z)/20:5(5Z,8Z,11Z,14Z,17Z)/22:2(13Z,16Z))[iso6]

Table SI-2. 6-(OA) Smile of Lipid species TAG from LA condition (Lipid maps)

LM ID	SMILES
TGOOH	
TAG 54:9	
**LMGL03014806**	C(OC(=O)CC/C=C\C/C=C\C/C=C\C/C=C\C/C=C\C/C=C\CC)[C@]([H])(OC(CCCCCCC/C=C\C/C=C\CCCCC)=O)COC(CCCCCCC/C=C\CCCC)=O
**LMGL03016289**	C(OC(=O)CCCC/C=C\C/C=C\C/C=C\C/C=C\CC)[C@]([H])(OC(CCCC/C=C\C/C=C\C/C=C\CCCCC)=O)COC(CCCCCCC/C=C\C/C=C\CCCCC)=O
**LMGL03016306**	C(OC(=O)CCCC/C=C\C/C=C\C/C=C\C/C=C\CC)[C@]([H])(OC(CCCCCCC/C=C\C/C=C\C/C=C\CC)=O)COC(CCCCCCC/C=C\C/C=C\CCCCC)=O
TAG 56:7	
**LMGL03010734**	C(OC(=O)CCCCCC/C=C\C/C=C\C/C=C\CCCCC)[C@]([H])(OC(CCCCCCC/C=C\C/C=C\CCCCC)=O)COC(CCCCCCC/C=C\C/C=C\CCCCC)=O
**LMGL03010741**	C(OC(=O)CCC/C=C\C/C=C\C/C=C\C/C=C\CCCCC)[C@]([H])(OC(CCCCCCC/C=C\C/C=C\CCCCC)=O)COC(CCCCCCC/C=C\CCCCCCCC)=O
**LMGL03010747**	C(OC(=O)CCC/C=C\C/C=C\C/C=C\C/C=C\C/C=C\CC)[C@]([H])(OC(CCCCCCC/C=C\C/C=C\CCCCC)=O)COC(CCCCCCCCCCCCCCCCC)=O
**LMGL03010917**	C(OC(=O)CCCCC/C=C\C/C=C\C/C=C\C/C=C\CCCCC)[C@]([H])(OC(CCCCCCC/C=C\C/C=C\CCCCC)=O)COC(CCCCCCC/C=C\CCCCCC)=O
**LMGL03010921**	C(OC(=O)CCCCC/C=C\C/C=C\C/C=C\C/C=C\C/C=C\CC)[C@]([H])(OC(CCCCCCC/C=C\C/C=C\CCCCC)=O)COC(CCCCCCCCCCCCCCC)=O
**LMGL03016294**	C(OC(=O)CCCCCCCCC/C=C\C/C=C\CCCCC)[C@]([H])(OC(CCCC/C=C\C/C=C\C/C=C\CCCCC)=O)COC(CCCCCCC/C=C\C/C=C\CCCCC)=O
**LMGL03016312**	C(OC(=O)CCCCCCCCC/C=C\CCCCCCCC)[C@]([H])(OC(CCCC/C=C\C/C=C\C/C=C\C/C=C\CC)=O)COC(CCCCCCC/C=C\C/C=C\CCCCC)=O
TAG 58:10	
**LMGL03011316**	C(OC(=O)CCC/C=C\C/C=C\C/C=C\C/C=C\CCCCC)[C@]([H])(OC(CCC/C=C\C/C=C\C/C=C\C/C=C\CCCCC)=O)COC(CCCCCCC/C=C\C/C=C\CCCCC)=O
**LMGL03011322**	C(OC(=O)CCC/C=C\C/C=C\C/C=C\C/C=C\C/C=C\CC)[C@]([H])(OC(CCCCCC/C=C\C/C=C\C/C=C\CCCCC)=O)COC(CCCCCCC/C=C\C/C=C\CCCCC)=O
**LMGL03011536**	C(OC(=O)CCCCC/C=C\C/C=C\C/C=C\C/C=C\C/C=C\CC)[C@]([H])(OC(CCCCCCC/C=C\C/C=C\C/C=C\CC)=O)COC(CCCCCCC/C=C\C/C=C\CCCCC)=O
**LMGL03011544**	C(OC(=O)CC/C=C\C/C=C\C/C=C\C/C=C\C/C=C\C/C=C\CC)[C@]([H])(OC(CCCCCCC/C=C\C/C=C\CCCCC)=O)COC(CCCCCCC/C=C\C/C=C\CCCCC)=O
**LMGL03016304**	C(OC(=O)CCCCC/C=C\C/C=C\C/C=C\C/C=C\C/C=C\CC)[C@]([H])(OC(CCCC/C=C\C/C=C\C/C=C\CCCCC)=O)COC(CCCCCCC/C=C\C/C=C\CCCCC)=O
**LMGL03016322**	C(OC(=O)CCCCC/C=C\C/C=C\C/C=C\C/C=C\CCCCC)[C@]([H])(OC(CCCC/C=C\C/C=C\C/C=C\C/C=C\CC)=O)COC(CCCCCCC/C=C\C/C=C\CCCCC)=O
TAG 58:11	
LMGL03011398	C(OC(=O)CCC/C=C\C/C=C\C/C=C\C/C=C\C/C=C\CC)[C@]([H])(OC(CCC/C=C\C/C=C\C/C=C\C/C=C\CCCCC)=O)COC(CCCCCCC/C=C\C/C=C\CCCCC)=O
LMGL03011620	C(OC(=O)CC/C=C\C/C=C\C/C=C\C/C=C\C/C=C\C/C=C\CC)[C@]([H])(OC(CCCCCCC/C=C\C/C=C\C/C=C\CC)=O)COC(CCCCCCC/C=C\C/C=C\CCCCC)=O
LMGL03016305	C(OC(=O)CC/C=C\C/C=C\C/C=C\C/C=C\C/C=C\C/C=C\CC)[C@]([H])(OC(CCCC/C=C\C/C=C\C/C=C\CCCCC)=O)COC(CCCCCCC/C=C\C/C=C\CCCCC)=O
LMGL03016323	C(OC(=O)CCCCC/C=C\C/C=C\C/C=C\C/C=C\C/C=C\CC)[C@]([H])(OC(CCCC/C=C\C/C=C\C/C=C\C/C=C\CC)=O)COC(CCCCCCC/C=C\C/C=C\CCCCC)=O

LM ID	SMILES
TAG(OOH)2	
TG 54:3	
LMGL03010252	C(OC(=O)CCCCCCC/C=C\C/C=C\CCCCC)[C@]([H])(OC(CCCCCCC/C=C\CCCCCCCC)=O)COC(CCCCCCCCCCCCCCCCC)=O
LMGL03010263	C(OC(=O)CCCCCCCCCCCCCCCCCCC)[C@]([H])(OC(CCCCCCC/C=C\C/C=C\CCCCC)=O)COC(CCCCCCC/C=C\CCCCCC)=O
LMGL03010267	C(OC(=O)CCCCCCCCC/C=C\CCCCCCCC)[C@]([H])(OC(CCCCCCC/C=C\C/C=C\CCCCC)=O)COC(CCCCCCCCCCCCCCC)=O
LMGL03010294	C(OC(=O)CCCCCCCCCCCCCCCCCC)[C@]([H])(OC(CCCCCCC/C=C\C/C=C\CCCCC)=O)COC(CCCCCCC/C=C\CCCCCCC)=O
LMGL03014395	C(OC(=O)CCCCCCCCC/C=C\CCCCCCCCCC)[C@]([H])(OC(CCCCCCC/C=C\C/C=C\CCCCC)=O)COC(CCCCCCCCCCCCC)=O
LMGL03014800	C(OC(=O)CCCCCCCCCCCCCCCCCCCCC)[C@]([H])(OC(CCCCCCC/C=C\C/C=C\CCCCC)=O)COC(CCCCCCC/C=C\CCCC)=O
LMGL03015528	C(OC(=O)CCCCCCCCCCCCCCCCCCCC)[C@]([H])(OC(CCCCCCC/C=C\C/C=C\CCCCC)=O)COC(CCCCCCC/C=C\CCCCC)=O
LMGL03015911	C(OC(=O)CCCCCCC/C=C\CCCCCCCCC)[C@]([H])(OC(CCCCCCC/C=C\C/C=C\CCCCC)=O)COC(CCCCCCCCCCCCCCCC)=O
TAG 62:13	
LMGL03012263	C(OC(=O)CCCCCCCC/C=C\C/C=C\C/C=C\CCCCC)[C@]([H])(OC(CCC/C=C\C/C=C\C/C=C\C/C=C\C/C=C\CC)=O)COC(CCC/C=C\C/C=C\C/C=C\C/C=C\C/C=C\CC)=O
LMGL03012318	C(OC(=O)CCCCC/C=C\C/C=C\C/C=C\C/C=C\CCCCC)[C@]([H])(OC(CCC/C=C\C/C=C\C/C=C\C/C=C\C/C=C\CC)=O)COC(CCC/C=C\C/C=C\C/C=C\C/C=C\CCCCC)=O
LMGL03012326	C(OC(=O)CCCCC/C=C\C/C=C\C/C=C\C/C=C\C/C=C\CC)[C@]([H])(OC(CCC/C=C\C/C=C\C/C=C\C/C=C\C/C=C\CC)=O)COC(CCCCCC/C=C\C/C=C\C/C=C\CCCCC)=O
LMGL03012327	C(OC(=O)CCCCC/C=C\C/C=C\C/C=C\C/C=C\C/C=C\CC)[C@]([H])(OC(CCC/C=C\C/C=C\C/C=C\C/C=C\CCCCC)=O)COC(CCC/C=C\C/C=C\C/C=C\C/C=C\CCCCC)=O
LMGL03012336	C(OC(=O)CC/C=C\C/C=C\C/C=C\C/C=C\C/C=C\C/C=C\CC)[C@]([H])(OC(CCC/C=C\C/C=C\C/C=C\C/C=C\C/C=C\CC)=O)COC(CCCCCCCCC/C=C\C/C=C\CCCCC)=O
LMGL03012337	C(OC(=O)CC/C=C\C/C=C\C/C=C\C/C=C\C/C=C\C/C=C\CC)[C@]([H])(OC(CCC/C=C\C/C=C\C/C=C\C/C=C\CCCCC)=O)COC(CCCCCC/C=C\C/C=C\C/C=C\CCCCC)=O
LMGL03012391	C(OC(=O)CCCCC/C=C\C/C=C\C/C=C\C/C=C\C/C=C\CC)[C@]([H])(OC(CCCCC/C=C\C/C=C\C/C=C\C/C=C\C/C=C\CC)=O)COC(CCCCCCC/C=C\C/C=C\C/C=C\CC)=O
LMGL03012401	C(OC(=O)CC/C=C\C/C=C\C/C=C\C/C=C\C/C=C\C/C=C\CC)[C@]([H])(OC(CCCCC/C=C\C/C=C\C/C=C\C/C=C\CCCCC)=O)COC(CCCCCCC/C=C\C/C=C\C/C=C\CC)=O
LMGL03012399	C(OC(=O)CC/C=C\C/C=C\C/C=C\C/C=C\C/C=C\C/C=C\CC)[C@]([H])(OC(CC/C=C\C/C=C\C/C=C\C/C=C\C/C=C\C/C=C\CC)=O)COC(CCCCCCC/C=C\CCCCCCCC)=O
LMGL03013079	C(OC(=O)CCCCC/C=C\C/C=C\C/C=C\C/C=C\C/C=C\CC)[C@]([H])(OC(CCCCC/C=C\C/C=C\C/C=C\C/C=C\C/C=C\CC)=O)COC(CCCC/C=C\C/C=C\C/C=C\CCCCC)=O
LMGL03016506	C(OC(=O)CC/C=C\C/C=C\C/C=C\C/C=C\C/C=C\C/C=C\CC)[C@]([H])(OC(CCCCC/C=C\C/C=C\C/C=C\C/C=C\CCCCC)=O)COC(CCCC/C=C\C/C=C\C/C=C\CCCCC)=O
LMGL03016670	C(OC(=O)CC/C=C\C/C=C\C/C=C\C/C=C\C/C=C\C/C=C\CC)[C@]([H])(OC(CCCCCCCC/C=C\C/C=C\C/C=C\CCCCC)=O)COC(CCCC/C=C\C/C=C\C/C=C\C/C=C\CC)=O
LMGL03016671	C(OC(=O)CCCCC/C=C\C/C=C\C/C=C\C/C=C\C/C=C\CC)[C@]([H])(OC(CCCCC/C=C\C/C=C\C/C=C\C/C=C\CCCCC)=O)COC(CCCC/C=C\C/C=C\C/C=C\C/C=C\CC)=O

LM ID	SMILES
TAG(OOH)3	
TAG 50:3	
LMGL03010053	C(OC(=O)CCCCCCC/C=C\C/C=C\CCCCC)[C@]([H])(OC(CCCCCCC/C=C\CCCCCC)=O)COC(CCCCCCCCCCCCCCC)=O
LMGL03013488	C(OC(=O)CCCCCCCCC/C=C\CCCCCCCC)[C@]([H])(OC(CCCCCCC/C=C\C/C=C\CCCCC)=O)COC(CCCCCCCCCCC)=O
LMGL03013951	C(OC(=O)CCCCCCC/C=C\CCCCCCCCC)[C@]([H])(OC(CCCCCCC/C=C\C/C=C\CCCCC)=O)COC(CCCCCCCCCCCCC)=O
LMGL03014362	C(OC(=O)CCCCCCC/C=C\C/C=C\CCCCC)[C@]([H])(OC(CCCCCCC/C=C\CCCCCCCC)=O)COC(CCCCCCCCCCCCC)=O
LMGL03014748	C(OC(=O)CCCCCCC/C=C\C/C=C\CCCCC)[C@]([H])(OC(CCCCCCCCCCCCCCCCC)=O)COC(CCCCCCC/C=C\CCCC)=O
LMGL03015083	C(OC(=O)CCCCCCC/C=C\C/C=C\CCCCC)[C@]([H])(OC(CCCCCCC/C=C\CCCCCCC)=O)COC(CCCCCCCCCCCCCCC)=O
LMGL03015411	C(OC(=O)CCCCCCC/C=C\C/C=C\CCCCC)[C@]([H])(OC(CCCCCCCCCCCCCCCCC)=O)COC(CCCCCCC/C=C\CCCCC)=O
TAG 52:5	
LMGL03010144	C(OC(=O)CCCCCCC/C=C\C/C=C\CCCCC)[C@]([H])(OC(CCCCCCC/C=C\C/C=C\CCCC)=O)COC(CCCCCCC/C=C\CCCCCCC)=O
LMGL03010163	C(OC(=O)CCCCCCC/C=C\C/C=C\CCCCC)[C@]([H])(OC(CCCCCCC/C=C\C/C=C\CCCCC)=O)COC(CCCCCCC/C=C\CCCCCC)=O
LMGL03010167	C(OC(=O)CCCCCCC/C=C\C/C=C\C/C=C\CC)[C@]([H])(OC(CCCCCCC/C=C\C/C=C\CCCCC)=O)COC(CCCCCCCCCCCCCCC)=O
LMGL03013497	C(OC(=O)CCCCCCCC/C=C\C/C=C\C/C=C\CCCCC)[C@]([H])(OC(CCCCCCC/C=C\C/C=C\CCCCC)=O)COC(CCCCCCCCCCC)=O
LMGL03014390	C(OC(=O)CCCCCC/C=C\C/C=C\C/C=C\CCCCC)[C@]([H])(OC(CCCCCCC/C=C\C/C=C\CCCCC)=O)COC(CCCCCCCCCCCCC)=O
LMGL03014795	C(OC(=O)CCCCCCCCC/C=C\C/C=C\CCCCC)[C@]([H])(OC(CCCCCCC/C=C\C/C=C\CCCCC)=O)COC(CCCCCCC/C=C\CCCC)=O
LMGL03015731	C(OC(=O)CCCC/C=C\C/C=C\C/C=C\CCCCC)[C@]([H])(OC(CCCCCCC/C=C\C/C=C\CCCCC)=O)COC(CCCCCCCCCCCCCCC)=O
TAG 54:10	
LMGL03012804	C(OC(=O)CCC/C=C\C/C=C\C/C=C\C/C=C\C/C=C\CC)[C@]([H])(OC(CCC/C=C\C/C=C\C/C=C\C/C=C\C/C=C\CC)=O)COC(CCCCCCCCCCCCC)=O
LMGL03013046	C(OC(=O)CCCC/C=C\C/C=C\C/C=C\C/C=C\CC)[C@]([H])(OC(CCCC/C=C\C/C=C\C/C=C\CCCCC)=O)COC(CCCC/C=C\C/C=C\C/C=C\CCCCC)=O
LMGL03013081	C(OC(=O)CCCC/C=C\C/C=C\C/C=C\C/C=C\CC)[C@]([H])(OC(CCCCCCC/C=C\C/C=C\C/C=C\CC)=O)COC(CCCCCCC/C=C\C/C=C\C/C=C\CC)=O
LMGL03013635	C(OC(=O)CC/C=C\C/C=C\C/C=C\C/C=C\C/C=C\C/C=C\CC)[C@]([H])(OC(CCC/C=C\C/C=C\C/C=C\C/C=C\CCCCC)=O)COC(CCCCCCCCCCC)=O
LMGL03013642	C(OC(=O)CCCCC/C=C\C/C=C\C/C=C\C/C=C\C/C=C\CC)[C@]([H])(OC(CCC/C=C\C/C=C\C/C=C\C/C=C\C/C=C\CC)=O)COC(CCCCCCCCCCC)=O
LMGL03014451	C(OC(=O)CC/C=C\C/C=C\C/C=C\C/C=C\C/C=C\C/C=C\CC)[C@]([H])(OC(CCCC/C=C\C/C=C\C/C=C\C/C=C\CC)=O)COC(CCCCCCCCCCCCC)=O
LMGL03014824	C(OC(=O)CC/C=C\C/C=C\C/C=C\C/C=C\C/C=C\C/C=C\CC)[C@]([H])(OC(CCCC/C=C\C/C=C\C/C=C\CCCCC)=O)COC(CCCCCCC/C=C\CCCC)=O
LMGL03014841	C(OC(=O)CC/C=C\C/C=C\C/C=C\C/C=C\C/C=C\C/C=C\CC)[C@]([H])(OC(CCCCCCC/C=C\C/C=C\C/C=C\CC)=O)COC(CCCCCCC/C=C\CCCC)=O
LMGL03014856	C(OC(=O)CCCCC/C=C\C/C=C\C/C=C\C/C=C\C/C=C\CC)[C@]([H])(OC(CCCC/C=C\C/C=C\C/C=C\C/C=C\CC)=O)COC(CCCCCCC/C=C\CCCC)=O

LMGL03014933	C(OC(=O)CCC/C=C\C/C=C\C/C=C\C/C=C\C/C=C\CC)[C@]([H])(OC(CCC/C=C\C/C=C\C/C=C\C/C=C\CCCCC)=O)COC(CCCCCCC/C=C\CCCC)=O
LMGL03015854	C(OC(=O)CCC/C=C\C/C=C\C/C=C\C/C=C\C/C=C\CC)[C@]([H])(OC(CCCC/C=C\C/C=C\C/C=C\C/C=C\CC)=O)COC(CCCCCCC/C=C\CCCCCC)=O
LMGL03016355	C(OC(=O)CCCC/C=C\C/C=C\C/C=C\C/C=C\CC)[C@]([H])(OC(CCCCCCC/C=C\C/C=C\C/C=C\CC)=O)COC(CCCC/C=C\C/C=C\C/C=C\CCCCC)=O
TAG 54:4	
LMGL03010287	C(OC(=O)CCCCCCC/C=C\C/C=C\CCCCC)[C@]([H])(OC(CCCCCCC/C=C\C/C=C\CCCCC)=O)COC(CCCCCCCCCCCCCCCCC)=O
LMGL03010288	C(OC(=O)CCCCCCC/C=C\C/C=C\CCCCC)[C@]([H])(OC(CCCCCCC/C=C\CCCCCCCC)=O)COC(CCCCCCC/C=C\CCCCCCCC)=O
LMGL03010303	C(OC(=O)CCCCCCCCC/C=C\CCCCCCCC)[C@]([H])(OC(CCCCCCC/C=C\C/C=C\CCCCC)=O)COC(CCCCCCC/C=C\CCCCCC)=O
LMGL03010307	C(OC(=O)CCCCCCCCC/C=C\C/C=C\CCCCC)[C@]([H])(OC(CCCCCCC/C=C\C/C=C\CCCCC)=O)COC(CCCCCCCCCCCCCCC)=O
LMGL03010333	C(OC(=O)CCCCCCCCCCCCCCCCC)[C@]([H])(OC(CCCCCCC/C=C\C/C=C\CCCCC)=O)COC(CCCCCCC/C=C\C/C=C\CCCC)=O
LMGL03014396	C(OC(=O)CCCCCCCCCCC/C=C\C/C=C\CCCCC)[C@]([H])(OC(CCCCCCC/C=C\C/C=C\CCCCC)=O)COC(CCCCCCCCCCCCC)=O
LMGL03014801	C(OC(=O)CCCCCCCCC/C=C\CCCCCCCCCC)[C@]([H])(OC(CCCCCCC/C=C\C/C=C\CCCCC)=O)COC(CCCCCCC/C=C\CCCC)=O
LMGL03015994	C(OC(=O)CCCCCCC/C=C\CCCCCCCCC)[C@]([H])(OC(CCCCCCC/C=C\C/C=C\CCCCC)=O)COC(CCCCCCC/C=C\CCCCCCC)=O
LMGL03010291	C(OC(=O)CCCCCCC/C=C\C/C=C\C/C=C\CC)[C@]([H])(OC(CCCCCCC/C=C\CCCCCCCC)=O)COC(CCCCCCCCCCCCCCCCC)=O
LMGL03013022	C(OC(=O)CCCC/C=C\C/C=C\C/C=C\C/C=C\CC)[C@]([H])(OC(CCCCCCCCCCCCCCCCC)=O)COC(CCCCCCCCCCCCCCCCC)=O
LMGL03016142	C(OC(=O)CCCC/C=C\C/C=C\C/C=C\CCCCC)[C@]([H])(OC(CCCCCCC/C=C\CCCCCCCC)=O)COC(CCCCCCCCCCCCCCCCC)=O
TG 56:10	
LMGL03010946	C(OC(=O)CCC/C=C\C/C=C\C/C=C\C/C=C\CCCCC)[C@]([H])(OC(CCCCCCC/C=C\C/C=C\C/C=C\CC)=O)COC(CCCCCCC/C=C\C/C=C\C/C=C\CC)=O
LMGL03010947	C(OC(=O)CCC/C=C\C/C=C\C/C=C\C/C=C\C/C=C\CC)[C@]([H])(OC(CCC/C=C\C/C=C\C/C=C\C/C=C\C/C=C\CC)=O)COC(CCCCCCCCCCCCCCC)=O
LMGL03010948	C(OC(=O)CCC/C=C\C/C=C\C/C=C\C/C=C\C/C=C\CC)[C@]([H])(OC(CCC/C=C\C/C=C\C/C=C\C/C=C\CCCCC)=O)COC(CCCCCCC/C=C\CCCCCC)=O
LMGL03011078	C(OC(=O)CC/C=C\C/C=C\C/C=C\C/C=C\C/C=C\C/C=C\CC)[C@]([H])(OC(CCCCCCC/C=C\C/C=C\CCCC)=O)COC(CCCCCCC/C=C\C/C=C\CCCC)=O
LMGL03011152	C(OC(=O)CC/C=C\C/C=C\C/C=C\C/C=C\C/C=C\C/C=C\CC)[C@]([H])(OC(CCCCCCC/C=C\C/C=C\C/C=C\CC)=O)COC(CCCCCCC/C=C\CCCCCC)=O
LMGL03012689	C(OC(=O)CCCCC/C=C\C/C=C\C/C=C\C/C=C\C/C=C\CC)[C@]([H])(OC(CCCCC/C=C\C/C=C\C/C=C\C/C=C\C/C=C\CC)=O)COC(CCCCCCCCCCC)=O
LMGL03013053	C(OC(=O)CCC/C=C\C/C=C\C/C=C\C/C=C\CCCCC)[C@]([H])(OC(CCCC/C=C\C/C=C\C/C=C\CCCCC)=O)COC(CCCC/C=C\C/C=C\C/C=C\CCCCC)=O
LMGL03013091	C(OC(=O)CCCCCCCCC/C=C\C/C=C\CCCCC)[C@]([H])(OC(CCCC/C=C\C/C=C\C/C=C\C/C=C\CC)=O)COC(CCCC/C=C\C/C=C\C/C=C\C/C=C\CC)=O
LMGL03013670	C(OC(=O)CC/C=C\C/C=C\C/C=C\C/C=C\C/C=C\C/C=C\CC)[C@]([H])(OC(CCCCC/C=C\C/C=C\C/C=C\C/C=C\CCCCC)=O)COC(CCCCCCCCCCC)=O

LMGL03014535	C(OC(=O)CC/C=C\C/C=C\C/C=C\C/C=C\C/C=C\C/C=C\CC)[C@]([H])(OC(CCC/C=C\C/C=C\C/C=C\C/C=C\CCCCC)=O)COC(CCCCCCCCCCCCC)=O
LMGL03014542	C(OC(=O)CCCCC/C=C\C/C=C\C/C=C\C/C=C\C/C=C\CC)[C@]([H])(OC(CCC/C=C\C/C=C\C/C=C\C/C=C\C/C=C\CC)=O)COC(CCCCCCCCCCCCC)=O
LMGL03014932	C(OC(=O)CC/C=C\C/C=C\C/C=C\C/C=C\C/C=C\C/C=C\CC)[C@]([H])(OC(CCCCCC/C=C\C/C=C\C/C=C\CCCCC)=O)COC(CCCCCCC/C=C\CCCC)=O
LMGL03014940	C(OC(=O)CCCCC/C=C\C/C=C\C/C=C\C/C=C\C/C=C\CC)[C@]([H])(OC(CCC/C=C\C/C=C\C/C=C\C/C=C\CCCCC)=O)COC(CCCCCCC/C=C\CCCC)=O
LMGL03014947	C(OC(=O)CCCCC/C=C\C/C=C\C/C=C\C/C=C\CCCCC)[C@]([H])(OC(CCC/C=C\C/C=C\C/C=C\C/C=C\C/C=C\CC)=O)COC(CCCCCCC/C=C\CCCC)=O
LMGL03015771	C(OC(=O)CC/C=C\C/C=C\C/C=C\C/C=C\C/C=C\C/C=C\CC)[C@]([H])(OC(CCCC/C=C\C/C=C\C/C=C\C/C=C\CC)=O)COC(CCCCCCCCCCCCCCC)=O
LMGL03015843	C(OC(=O)CC/C=C\C/C=C\C/C=C\C/C=C\C/C=C\C/C=C\CC)[C@]([H])(OC(CCCC/C=C\C/C=C\C/C=C\CCCCC)=O)COC(CCCCCCC/C=C\CCCCCC)=O
LMGL03015861	C(OC(=O)CCCCC/C=C\C/C=C\C/C=C\C/C=C\C/C=C\CC)[C@]([H])(OC(CCCC/C=C\C/C=C\C/C=C\C/C=C\CC)=O)COC(CCCCCCC/C=C\CCCCCC)=O
LMGL03016249	C(OC(=O)CCC/C=C\C/C=C\C/C=C\C/C=C\C/C=C\CC)[C@]([H])(OC(CCCC/C=C\C/C=C\C/C=C\C/C=C\CC)=O)COC(CCCCCCC/C=C\CCCCCCCC)=O
LMGL03016362	C(OC(=O)CCC/C=C\C/C=C\C/C=C\C/C=C\CCCCC)[C@]([H])(OC(CCCCCCC/C=C\C/C=C\C/C=C\CC)=O)COC(CCCC/C=C\C/C=C\C/C=C\CCCCC)=O
LMGL03016377	C(OC(=O)CCCCCC/C=C\C/C=C\C/C=C\CCCCC)[C@]([H])(OC(CCCC/C=C\C/C=C\C/C=C\C/C=C\CC)=O)COC(CCCC/C=C\C/C=C\C/C=C\CCCCC)=O
LMGL03016513	C(OC(=O)CCCCCC/C=C\C/C=C\C/C=C\CCCCC)[C@]([H])(OC(CCCC/C=C\C/C=C\C/C=C\C/C=C\CC)=O)COC(CCCCCCC/C=C\C/C=C\C/C=C\CC)=O

**LMGL0301D7GQ**	Triacylglycerols [GL0301]
TAG 58:7	
LMGL03011298	C(OC(=O)CCCCC/C=C\C/C=C\C/C=C\C/C=C\CCCCC)[C@]([H])(OC(CCCCCCC/C=C\C/C=C\CCCCC)=O)COC(CCCCCCC/C=C\CCCCCCCC)=O
LMGL03011304	C(OC(=O)CCCCC/C=C\C/C=C\C/C=C\C/C=C\C/C=C\CC)[C@]([H])(OC(CCCCCCC/C=C\C/C=C\CCCCC)=O)COC(CCCCCCCCCCCCCCCCC)=O
TAG 60:11	
LMGL03011769	C(OC(=O)CCC/C=C\C/C=C\C/C=C\C/C=C\CCCCC)[C@]([H])(OC(CCC/C=C\C/C=C\C/C=C\C/C=C\CCCCC)=O)COC(CCCCCC/C=C\C/C=C\C/C=C\CCCCC)=O
LMGL03011770	C(OC(=O)CCC/C=C\C/C=C\C/C=C\C/C=C\C/C=C\CC)[C@]([H])(OC(CCC/C=C\C/C=C\C/C=C\C/C=C\C/C=C\CC)=O)COC(CCCCCCCCC/C=C\CCCCCCCC)=O
LMGL03011772	C(OC(=O)CCC/C=C\C/C=C\C/C=C\C/C=C\C/C=C\CC)[C@]([H])(OC(CCCCCC/C=C\C/C=C\C/C=C\CCCCC)=O)COC(CCCCCC/C=C\C/C=C\C/C=C\CCCCC)=O
LMGL03011771	C(OC(=O)CCC/C=C\C/C=C\C/C=C\C/C=C\C/C=C\CC)[C@]([H])(OC(CCC/C=C\C/C=C\C/C=C\C/C=C\CCCCC)=O)COC(CCCCCCCCC/C=C\C/C=C\CCCCC)=O
LMGL03011863	C(OC(=O)CCCCCCCC/C=C\C/C=C\C/C=C\CCCCC)[C@]([H])(OC(CCC/C=C\C/C=C\C/C=C\C/C=C\C/C=C\CC)=O)COC(CCCCCCC/C=C\C/C=C\C/C=C\CC)=O
LMGL03011949	C(OC(=O)CCCCC/C=C\C/C=C\C/C=C\C/C=C\CCCCC)[C@]([H])(OC(CCC/C=C\C/C=C\C/C=C\C/C=C\CCCCC)=O)COC(CCCCCCC/C=C\C/C=C\C/C=C\CC)=O
LMGL03011959	C(OC(=O)CCCCC/C=C\C/C=C\C/C=C\C/C=C\C/C=C\CC)[C@]([H])(OC(CCC/C=C\C/C=C\C/C=C\C/C=C\C/C=C\CC)=O)COC(CCCCCCC/C=C\CCCCCCCC)=O
LMGL03011961	C(OC(=O)CCCCC/C=C\C/C=C\C/C=C\C/C=C\C/C=C\CC)[C@]([H])(OC(CCCCCC/C=C\C/C=C\C/C=C\CCCCC)=O)COC(CCCCCCC/C=C\C/C=C\C/C=C\CC)=O
LMGL03011969	C(OC(=O)CC/C=C\C/C=C\C/C=C\C/C=C\C/C=C\C/C=C\CC)[C@]([H])(OC(CCC/C=C\C/C=C\C/C=C\C/C=C\C/C=C\CC)=O)COC(CCCCCCCCCCCCCCCCC)=O
LMGL03011970	C(OC(=O)CC/C=C\C/C=C\C/C=C\C/C=C\C/C=C\C/C=C\CC)[C@]([H])(OC(CCC/C=C\C/C=C\C/C=C\C/C=C\CCCCC)=O)COC(CCCCCCC/C=C\CCCCCCCC)=O
LMGL03011972	C(OC(=O)CC/C=C\C/C=C\C/C=C\C/C=C\C/C=C\C/C=C\CC)[C@]([H])(OC(CCCCCCCCC/C=C\C/C=C\CCCCC)=O)COC(CCCCCCC/C=C\C/C=C\C/C=C\CC)=O

LMGL03012075	C(OC(=O)CCCCC/C=C\C/C=C\C/C=C\C/C=C\C/C=C\CC)[C@]([H])(OC(CCCCC/C=C\C/C=C\C/C=C\C/C=C\C/C=C\CC)=O)COC(CCCCCCC/C=C\CCCCCC)=O
LMGL03012086	C(OC(=O)CC/C=C\C/C=C\C/C=C\C/C=C\C/C=C\C/C=C\CC)[C@]([H])(OC(CCCCC/C=C\C/C=C\C/C=C\C/C=C\C/C=C\CC)=O)COC(CCCCCCCCCCCCCCC)=O
LMGL03012087	C(OC(=O)CC/C=C\C/C=C\C/C=C\C/C=C\C/C=C\C/C=C\CC)[C@]([H])(OC(CCCCC/C=C\C/C=C\C/C=C\C/C=C\CCCCC)=O)COC(CCCCCCC/C=C\CCCCCC)=O
LMGL03016452	C(OC(=O)CC/C=C\C/C=C\C/C=C\C/C=C\C/C=C\C/C=C\CC)[C@]([H])(OC(CCCCCCCCC/C=C\C/C=C\CCCCC)=O)COC(CCCC/C=C\C/C=C\C/C=C\CCCCC)=O
LMGL03016461	C(OC(=O)CCCCC/C=C\C/C=C\C/C=C\C/C=C\C/C=C\CC)[C@]([H])(OC(CCCCCC/C=C\C/C=C\C/C=C\CCCCC)=O)COC(CCCC/C=C\C/C=C\C/C=C\CCCCC)=O
LMGL03016469	C(OC(=O)CCCCC/C=C\C/C=C\C/C=C\C/C=C\CCCCC)[C@]([H])(OC(CCC/C=C\C/C=C\C/C=C\C/C=C\CCCCC)=O)COC(CCCC/C=C\C/C=C\C/C=C\CCCCC)=O
LMGL03016476	C(OC(=O)CCCCCCCC/C=C\C/C=C\C/C=C\CCCCC)[C@]([H])(OC(CCC/C=C\C/C=C\C/C=C\C/C=C\C/C=C\CC)=O)COC(CCCC/C=C\C/C=C\C/C=C\CCCCC)=O
LMGL03016626	C(OC(=O)CCCCC/C=C\C/C=C\C/C=C\C/C=C\CCCCC)[C@]([H])(OC(CCCCCC/C=C\C/C=C\C/C=C\CCCCC)=O)COC(CCCC/C=C\C/C=C\C/C=C\C/C=C\CC)=O
LMGL03016617	C(OC(=O)CCCCC/C=C\C/C=C\C/C=C\C/C=C\C/C=C\CC)[C@]([H])(OC(CCCCCCCCC/C=C\C/C=C\CCCCC)=O)COC(CCCC/C=C\C/C=C\C/C=C\C/C=C\CC)=O
LMGL03016607	C(OC(=O)CC/C=C\C/C=C\C/C=C\C/C=C\C/C=C\C/C=C\CC)[C@]([H])(OC(CCCCCCCCC/C=C\CCCCCCCC)=O)COC(CCCC/C=C\C/C=C\C/C=C\C/C=C\CC)=O
LMGL03016634	C(OC(=O)CCCCCCCC/C=C\C/C=C\C/C=C\CCCCC)[C@]([H])(OC(CCC/C=C\C/C=C\C/C=C\C/C=C\CCCCC)=O)COC(CCCC/C=C\C/C=C\C/C=C\C/C=C\CC)=O
LMGL03016641	C(OC(=O)CCCCCCCCCCC/C=C\C/C=C\CCCCC)[C@]([H])(OC(CCC/C=C\C/C=C\C/C=C\C/C=C\C/C=C\CC)=O)COC(CCCC/C=C\C/C=C\C/C=C\C/C=C\CC)=O
